# Rocks and clay: Potters’ technological choices within the cultural dynamics of Bronze Age Kazakhstan

**DOI:** 10.1371/journal.pone.0320140

**Published:** 2025-04-23

**Authors:** Paula N. Doumani Dupuy, Silvia Amicone, Marcel Frenken, Jack Berner, Taylor Hermes, Michael D. Frachetti, Galymzhan Kiyasbek

**Affiliations:** 1 Sociology and Anthropology Department, Nazarbayev University, Astana, Akmola Oblast, Kazakhstan; 2 Archaeometry Research Group, Eberhard-Karls-Universität Tübingen, Tübingen, Germany; 3 Institute of Archaeology, University College London, London, United Kingdom; 4 Science and Technology in Archaeology and Culture Research Center, The Cyprus Institute, Nicosia, Cyprus; 5 Anthropology Department, Washington University in St. Louis, St. Louis, Missouri, United States of America; 6 Department of Anthropology, University of Arkansas, Fayetteville, Arkansas, United States of America; 7 Margulan Institute of Archaeology, Almaty, Almaty Oblast, Kazakhstan; Istanbul University: Istanbul Universitesi, TÜRKIYE

## Abstract

Through regular interactions with their neighbors, diverse groups inhabiting areas along the Inner Asian Mountain Corridor during the Bronze Age formed dynamic interregional networks that saw the proliferation and persistence of shared material cultures over vast geographic areas. In this paper we advocate for ceramics analyses that combine both macro- and micro-scale technological studies alongside those of style, in order not to lose sight of the actual people who drove defining transformations in the Bronze Age. We present a petrographic study of pottery from the Zhetysu region, southeastern Kazakhstan, to examine diachronic technological traditions with a special focus on routines of selection and raw material processing. Our results demonstrate site-specific potting technologies as well as traits that transcend both time and space across episodes of high genetic turnover in the human population.

## Introduction

### Pottery and agropastoralists in Bronze Age Eurasia

Bronze Age populations of the Inner Asian Mountain Corridor (IAMC) lived throughout an epoch of growth in regional cohesion involving historically transformative demographic shifts and the proliferation of collective socio-economic and ritual behaviors [1]. References to the mountain foothills as an essential geographic feature appear throughout scholarship of central Eurasia to highlight the palimpsest of ancient social networks that took place prior to the flourishing of the historic Silk Roads [2,3]. While a broader appreciation of the IAMC has been increasingly met with studies conducted at the site scale, a cursory search of the literature will reveal preferential attention to subsistence and crop dispersals as opposed to material culture [4–10]. This research imbalance is surprising considering that ceramic and metal object assemblages, for example, provide the major bodies of evidence for pinpointing the timing and geographic scope of Bronze Age transformations in this region [11–16]. As a complete body of work, the literature to date undertheorizes the potential of pottery to disclose the materiality of small scale and more far-reaching community dynamics that contributed a lasting impact on Bronze Age social transformations.

**Fig 1:**
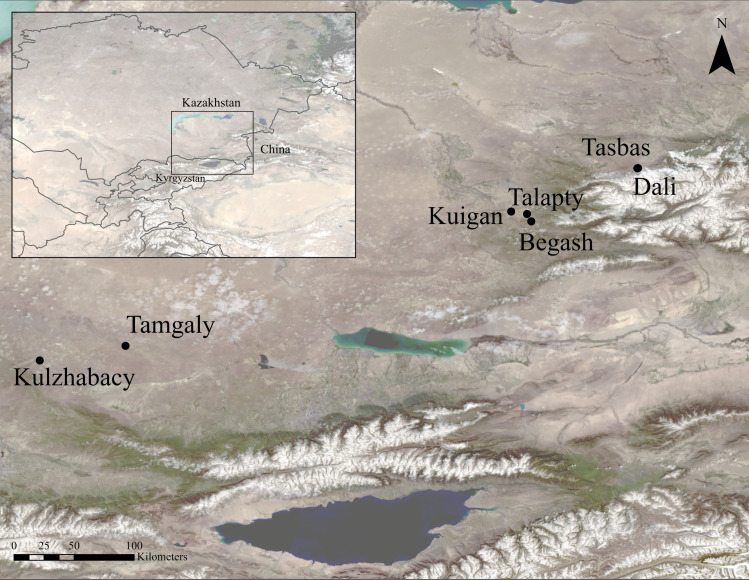
Map of study region showing sites* mentioned in the text. *Tasbas and Dali are indicated with a single marker due to their proximity. Landsat-9 imagery and NASA Shuttle Radar Topography Mission digital elevation model courtesy of the U.S. Geological Survey.

We build on those studies where the utilization of Bronze Age pottery assemblages has engaged with themes of materiality, ecology, and regional interactions through the mountains and linked steppe territories of Inner Asia [e.g., 12, 17–23]. Specifically, we interrogate the ceramic industries of transhumant mountain agropastoralists of Zhetysu (*Semirech’ye)* in southeastern Kazakhstan situated at a main crossroads of Eurasian interaction and exchange. Through detailed site-level data, we address open questions on inter-site and long-term mobility of people, objects, and knowledge frameworks through a multi-site study of ceramic technology across all Bronze Age periods. Our investigation applies petrographic analysis to ceramics recovered from both highland and lowland settlements to contribute a site-specific social-technological point of view of potters that is currently missing from the regional literature (Fig 1). The analysis provides the first published ceramic petrographic study that has been attempted for Zhetysu and for this portion of the IAMC and therefore contributes critical archaeological data for future studies of this kind (see [21] for a related study from the Kyrgyz IAMC pottery).

The investigated ceramics belong to a number of changing traditions that were present throughout the IAMC and linked Eurasian steppe between ca. 2700–800 cal BC. Within central Eurasia, as in other parts of the world, a long history of scholarship grounded in the culture-historical approach has relied on ceramic style in efforts to locate the territorial-geographic boundaries and overlap between archaeological cultures [13,24,25]. Bronze Age ceramic complexes of Zhetysu are today thus grouped and described according to pre-established regional classifications, although this literature reasonably targets assemblages dating from the mid-2nd millennium BC [26] because earlier Bronze Age occupations were unequivocally recognized only recently [17,27,28]. Ceramics from the earlier periods are as yet uncategorized into the culture-history, and here we provisionally group them as *‘Zhetysu’* (not to be mistaken for the *Semirech’ye Culture* pottery of the Late Bronze Age). Still to be investigated for all Bronze Age ceramics in Zhetysu is whether technologies of ceramic making were generally adopted by mountain inhabitants, or if locally confined traditions persisted within a regionally varied system. An investigation of this kind contributes knowledge on how human societies at the local scale respond to and continue to operate throughout and across periods of broadly sweeping social change and interaction. We consider ecological and social theoretical approaches while tracking back and forth between regional and site-level datasets to access the social-technological point of view of mountain potters from prehistory.

### Changing the narrative: A technological point of view

Visual culture including pottery containers can be a means for signaling connections as well as divisions in identity among residential and regional groups [29,30]. For societies engaged in residential mobility, seasonal movements and social interactions of various kinds can further influence all stages of the manufacture and life cycle of ceramics [31–35]. Pottery culture histories for Inner Asia outline considerable similarity within assemblages spanning the Eurasian steppes and mountainous regions from the mid-2nd millennium BC onward. Linked explanations for why the pottery is so similar across this region rest on the argument that pastoralists migrated and settled into all corners of central Eurasia and reproduced their traditions in the lands they newly settled [11,26]. More recently conducted paleogenetic studies of human samples taken from dozens of sites across Eurasia contribute a supportive line of evidence to the migration story and further highlight the input of genes from antecedent groups to later populations [36–39]. However, outside of pottery containers, the archaeological record of the steppe and IAMC show the two regions were quite different, each having their own distinct trajectories of change that unfolded as populations mixed across and between them [1]. Pottery culture histories for regions along the northern IAMC convey a scenario of active, outward, group affiliation achieved through high visibility attributes of pottery style – as opposed to divisions – but they cannot in turn uncover if other aspects of ceramic industries were more enduring. On the other hand, observing those less visible technological choices of potters [40] can help fine-tune explanations of long-term human-environment interactions, community structure and identity, and mobility among craftspeople. We consider these themes from a combined ceramic ecology and social-technological frameworks perspective to obtain a technological point of view of the Bronze Age potters of Zhetysu located along the northeastern stretch of the IAMC.

Several works have emphasized the due diligence of studying ancient artifacts given that they offer a window into traditions that are both socially constructed and in continuous evolution [e.g., 41–47]. The technological point of view while considering the role of choice in shaping pottery systems [48], additionally encompasses the perspective of potters, how they work and manipulate the landscape and its materials, and what traditions and knowledge they incorporate [49–51]. In moving the analysis beyond a principle focus on ceramic style, this approach explores the broader implications and influences that shape socio-economic behaviors and human-environment interactions [31,52–54]. To examine traditions of paste preparation in the manufacture of settlement pottery, we place a special focus on decision making in relation to routines of raw material selection and processing. The techniques used in ceramics manufacture include several linked steps starting from the initial phase of raw material procurement and ending with firing or post-firing surface treatment [55: Fig 4.3]. We target the material procurement and preparation phases as they are among the clearest reflections of discursive and non-discursive technical knowhow [56]. The skills needed for paste preparation are learned via hands-on instruction and training in motor skills and through access to environmental knowledge of where preferred raw materials and resources are found and how to manage them [55:111]. Here our focus on material procurement and preparation includes the steps of temper and clay collection, and the subsequent crushing, sorting, cleaning and paste mixing. This outlined emphasis makes it possible to investigate: 1) potential discontinuities in form and decoration that coincide with changes in local production practice, and 2) the nature of the relationship among mobility, tradition, and cultural choice in synchronic and diachronic perspective. Knowledge in this area will provide social context for the people and their ceramic industries in question lacking in the literature to offer a robust description of ancient pottery and technological practice among Bronze Age pastoralists of the IAMC.

## Materials and methods

An uncounted but great many Bronze Age habitation sites and cemeteries are present throughout Zhetysu that together show widespread exploitation of different elevational zones, seasonal pastures, and microenvironments [57–59]. Related ethnographic and archaeological documentation of these areas outline a system of flexible subsistence strategies that entailed vertical transhumance between summer and winter habitations, as well as the additional likelihood for periods of prolonged settlement in particular locales [19,60–63]. Seasonal movements of this type would have been an important factor underlying social-political alliances and shared community membership beyond any one site.

Ceramics sampled for the petrographic study originate from seven published settlements where the team has conducted excavations throughout the 2000s [19,27,28,61,64]. All necessary permits were obtained for the described study, which complied with all relevant regulations. The archaeological pottery samples excavated and archived during the 2000s were done so under projects operated by The Ministry of Culture, Information and Sports of the Republic of Kazakhstan and The Margulan Institute of Archaeology of the Republic of Kazakhstan (Permit No. 13003395). Petrographic analysis was performed under an agreement established on 29/12/2018 (No. 090118FD5330) between The Autonomous Organization of Education “Nazarbayev University” and Tubingen University, Germany within the framework of the project Trans-Eurasian Exchanges: Contemporary Dialogues and Archaeological Enquiry. Ceramic thin-section specimens reported in this study are housed with the Archaeometry Research Group, University of Tübingen and in the Anthropology Laboratory at Nazarbayev University in Astana.

The studied settlements are nestled along the southern side of mountain or foothill terraces and contain the remains of a few or a single domestic structure. Hence activities centered around craft production were small scale and presumably conducted at the household level. Ceramic wares do not vary between sites of the same time period, but techno-styles are noticeably different across the EBA, MBA and LBA within the study region. The sampling strategy therefore considered the macroscopic variability present among the pottery from each settlement and Bronze Age period and so is representative of the wares present in the study zone. A greater number of sherds were sampled from sites where there was more material available or with multi-period occupations, and for other sites where the collections were small or of a single occupation only, we sampled between five and ten sherds (Table 1, S1 Table and S1 Fig).

**Table 1:** Sites and samples present in the study. Counts of ceramic thin-section samples included in the analysis and corresponding Bronze Age phase, associated ceramic culture, and radiocarbon dates.

Settlement	Bronze Age Period	Regional Ceramic Culture	No. Sherds included in study	Cal. date BC, 95.4% (2σ)	Reference (for radiocarbon dates and site description)
Dali	Early	Zhetysu	9	2845–2545	[19]
	Middle	Andronovo	26	1840–1490	
	Late	Semirech’ye	4	1470–1150	
Tasbas	Late	Semirech’ye	35	1436–806	[27]
Begash	Early	Zhetysu	4	2470–1950	[28]
	Middle	Zhetysu, Andronovo	5	1950–1690	
	Late	Andronovo, Semirech’ye	29	1625–1000	
Kuigan 2	Early	Zhetysu	5	2468–2153	[64]
Kuigan 1	Late	Andronovo	5	None	
Talapty I	Early-Middle	Zhetysu	5	2122–1830	
Tamgaly I	Late	Semirech’ye	10	1250–950	[61]
Kulzhabacy V	Late	Semirech’ye	4	none	[61]
**Total Count**			**141**		

The parent settlement locations chosen for this study occupy a spectrum of microenvironments found in Zhetysu with radiocarbon and relative chronologies spanning the entire Bronze Age (Table 1), hence contributing a representative sampling of ceramics from domestic contexts. Specifically, we report on material from Dali and Tasbas from the highland meadows of the Bayan Zhurek valley, from Begash, Talapty-I and Kuigan (1 and 2) in the mid-elevation Koksu River valley and Eshkiolmes range, and from Tamgaly-I and Kulzhabacy-V located within the Chu-Ili, an extension of the Tian Shan arid steppe-foothills (Fig 1). The Chu-Ili Mountains form a system of low mountains oriented northwest with various geological layers formed by sedimentary and tectonic activity over the past two million years [65]. Kulzhabacy sits among mostly quaternary and sedimentary formations, whereas Tamgaly is situated by igneous formations as well. The mountain range unites several large hills (the highest point at 2200 m asl) under a common title that descend into foothill valleys surrounded by dry steppe and desert terrain. The axial part of the mountains supplies the upper watersheds of the Chu and Ili Rivers that empty into Lake Balkhash and, along with other major rivers of the Zhetysu (seven rivers) region (including the Koksu, Karatal, Byan, Aksu and Lepsi), defined a prehistoric domain of considerable contact and resource exchange between settlements throughout the Dzhungar and northern Tien Shan piedmont zones.

The geomorphological makeup of the Koksu River watershed can be divided between the upper and lower extents of the river, where the upper terraces and riparian zone are defined by rocky colluvium incised by high energy hydrological dynamics. Mixed metamorphic and sedimentary strata displaced by tectonic activity define the geology of the upper valley, whereas the lower valley is characterized by rolling loess deposits and abundant aeolian sedimentation.

By contrast, the Byan Zhurek piedmont zone reflects a glacially formed depression that is further eroded by high-energy fluvial cuts flowing northward and eastward from the Dzhungar Mountains. Large granite eratics, moraine deposits, and hydrological deposition shape the surface of the region, with sandy and loose gravel soils in abundance. The piedmont spur of the Byan Zhurek hills reflect two main geological areas, the eastern zone defined by exposed and heavily eroded granite bedrock and the western half defined by basalt cliffs, which made ideal surfaces for prehistoric rock art. Artesian springs are abundant especially in the eastern sector, and overall, the entire region is rich in active streams fed by glacial runoff and spring-water.

### Overview of the study sites

The earliest known Bronze Age sites in Zhetysu, such as Dali and Begash, were occupied at the start of the Early Bronze Age (EBA) during the 3^rd^ millennium BC by societies descended from earlier local hunter-gatherers and who engaged in small-scale metallurgy, multi-species animal husbandry and cultivation of millet for both ruminant and human consumption [19,58,66]. Paleo-environmental reconstructions [60] and stable isotope analyses [6] of fauna from those settlements indicate some amount of mobility during this early period wherein groups performed seasonal rounds of vertical transhumance; how this process impacted localized intra-site interactions is still unclear due to the paucity of additional EBA sites in Zhetysu. By the Middle Bronze Age (MBA) during the 2^nd^ millennium BC, altogether new regions became settled due to what scholars understand as the result of pastoral migrations and widespread demographic shifts occurring throughout Kazakhstan [58,67]. Some of the settlements in our study (i.e., Dali, Begash, Talapty, Kuigan, Tasbas) were occupied prior to, during, and after MBA demographic changes making it possible to investigate the potential impact that social transformations over generations had on pottery traditions. By the Late Bronze Age (LBA; mid/late 2nd millennium BC) there was a clear uptake of multi-cropping and local cultivation at sites such as Tasbas [68] although this practice is not uniformly documented at present throughout Zhetysu. Concomitantly new forms of architecture, such as the use of mudbrick for cooking installations, household floors, and ritual architecture appear alongside the continued emphasis on mixed economies and transhumance settlement patterns. Throughout the Bronze Age, metallurgy was increasingly incorporated by societies in Zhetysu, wherein household and semi-specialized smithing activities produced a wide array of body jewelry, working utensils, and weapons harboring techno-stylistic ties to the Eurasian steppe and other stretches of the IAMC [58,69]. Even more than for its metal inventory, ceramics from the 3rd and 2nd millennium BC have historically and continue to provide the major reference point for observing the internal divisions and population dynamics within Bronze Age central Eurasia.

### Pottery cultures of Bronze Age Zhetysu

A notable characteristic of Bronze Age ceramics from Zhetysu is their general likeness to ceramics of the same time period that are found throughout the Eurasian steppe and northeastern reaches of the IAMC [13,70]. These ceramics spanning the Early, Middle and Late Bronze Age have been arranged into regional culture histories wherein the literature is framed around broader discussions of how to trace and identify episodic migrations throughout large parts of central Eurasia [25,26,67]. We utilize the structuring contributions of the derived culture histories to be able to delve into the ceramic materiality of localized, transhumance movements, and to consider how such mobility may have played a role in manufacture and crafting practices over the long term. We address this topic through assessment of ceramic assemblages from sites with occupation histories that together offer representative insight into the changing ceramic traditions of the early through to the late Bronze Age.

**Fig 2:**
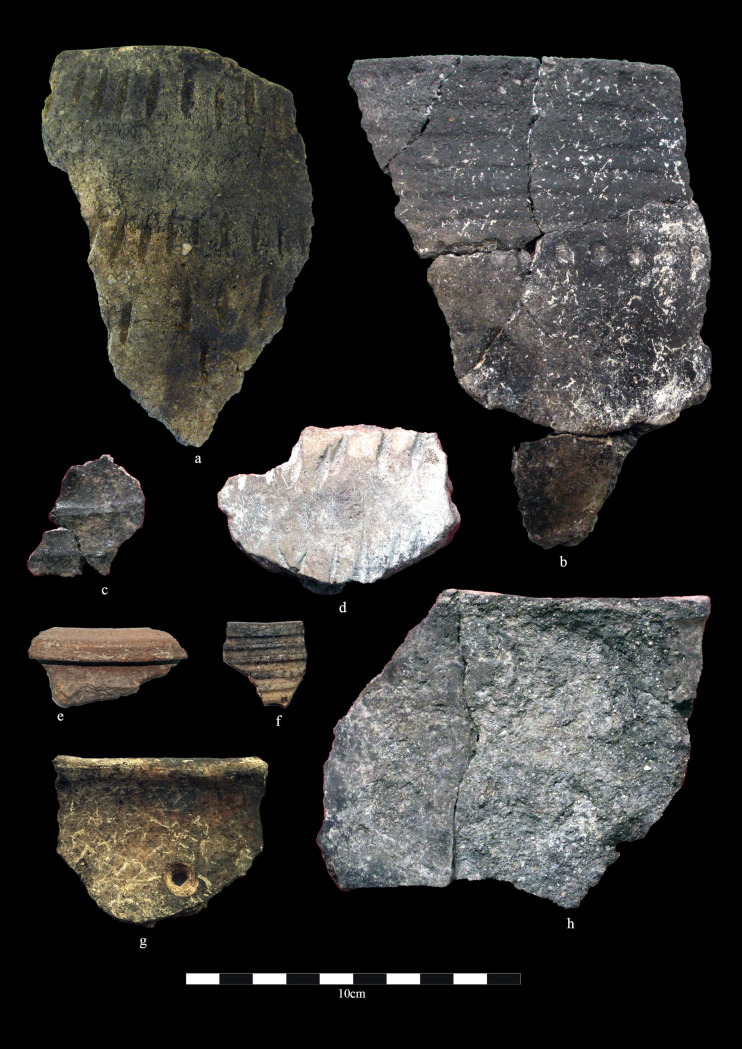
Early Bronze Age ceramics from Zhetysu settlements. Dali. a) DL-1458; b) DL-1009; Talapty. c) TL-4; d) TL-5; Begash. e) BG-1052; f) BG-1067; g) BG-0786; Kuigan 2. h) KG-5.

Ceramic classifications of EBA Zhetysu derive from materials recovered from a small handful of settlements with 3^rd^ millennium BC occupation layers. This study includes four of them: Dali and the slightly later settlements of Begash, Talapty and Kuigan 2 (Fig 2). The early pottery at Dali includes egg-shaped pots with thin walls, miniature bowls and pinch pots, and simple jars with thick walls and moderate volumetric dimensions. Complete vessels have not been recovered but based on the sherd assemblage we estimate that around half of the containers at Dali had decorations applied to the body, with heavy ornamentation sometimes covering the entire vessel surface. Frequently observed are punctate, speckled, incised lines, or comb-stamped geometric symbols that suggest an array of implements within potters’ toolkits. Containers were manufactured using a range of hand-building techniques (e.g., pinching and coiling) before being smoothed to a dull finish and then low-temperature fired to produce evenly colored black, dark brown, or pale gray finishes. Later on, as shown by the pottery obtained from Begash, Talapty and Kiugan 2, jars with thick walls, unmodified rims and heavy bases prevail [64: Fig 8 and 9]. Plain ware ceramics predominate at those three sites, but when vessels are decorated they show the use of almond-shaped and comb stamps, incising, or the creation of cordoned rims. Aside from the preponderance for undecorated containers, some formal design elements of the early 3^rd^ millennium BC Zhetysu ceramics are comparable to what has been documented for the Qiemu’erqieke (Chemurchek) and Okunev cultures in the Altai Mountains and eastern Kazakhstan, and the Elunino culture in the Kundula steppes of the Altai Krai, all regions located to the northeast of our study area.

**Fig 3:**
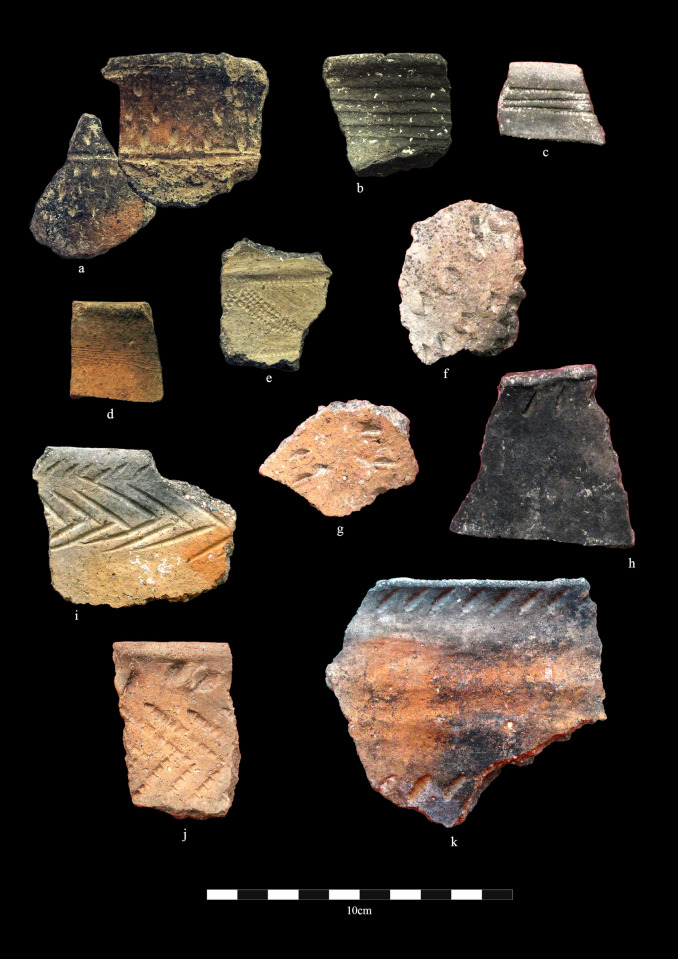
Middle Bronze Age ceramics from Zhetysu settlements. Begash. a) BG-0021; b) BG-0711; Kuigan. c) KG-8; Begash. d) BG-0018; e) BG-0666; LBA Kuigan. f) and g) KG-9, h) KG-10; Dali. i) DL-1017; j) DL-0449; k) DL-0774.

By the early 2^nd^ millennium BC, during the MBA, societies settled into all areas of Zhetysu and neighboring mountain regions and for this period there are a great many sites. In some cases, some sites, such as Dali, see a several hundred-year break in occupation between the EBA-MBA, whereas other sites, such as Begash and Talapty, reveal no such hiatus. The rise in settlement numbers is accompanied by the appearance of new styles of settlement pottery linked to the Andronovo Culture tradition. Assemblages for this period include thick-walled coarsewares and thin-walled finely manufactured vessels (Fig 3). Large open jar forms with high rounded shoulders or straight sides replace the egg-shaped pots of the EBA. Surface decorations of MBA ceramics show some continuity from the preceding phase by keeping to the upper zone of vessels. Overall, however, the array of designs increases into various iterations of comb-stamped, punctate, or incised combinations of geometric patterns (e.g., triangles, herringbones, linear bands, zigzags, and cross-hatching) along with the utilization of various implements for stamping, cutting, smoothing, and burnishing containers. Manufacturing techniques also diversify to include a range of hand-building approaches that combine slab-building (e.g., for pot bases) and coiling (e.g., for pot bodies and rims). Containers were low-fired resulting in mottled surfaces and core colors of black, red, or tan. MBA ceramics of Zhetysu share several stylistic and technological (e.g., vessel building methods) features with Alakul and eastern Fedorovo (Andronovo) culture ceramics of the eastern steppe zone and IAMC.

**Fig 4:**
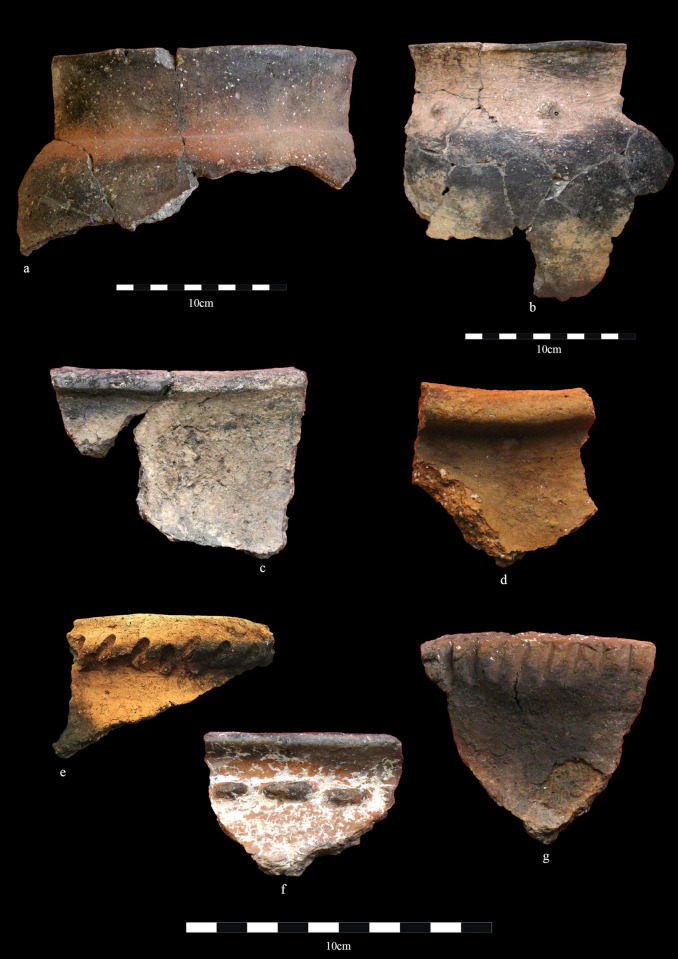
Late Bronze Age ceramics from Zhetysu settlements. Tamgaly. a) TM-10; b) TM-5; Kulzhabacy. c) KJ-4; Tasbas. d) TB-0136; Dali. e) DL-4-01; Tasbas. f) TB-0110; g) TB-0038.

The LBA of Zhetysu indicates yet another turnover in traditions of settlement structure, mortuary practice, material culture and subsistence [27]. During this period, the dominant vessel form includes goblet-shaped pots with high rounded shoulders and narrow bases, which are documented at most of the sites in this study (Fig 4). Surface designs are minimally placed at the lip, neck, or shoulder as clay appendages or cordons, along with simple stamped or incised patterns. Such designs appear on both thick-walled, heavy containers, and thin-walled burnished and slipped vessels. Vessels continue to be hand-built using coiling and slab-building methods as in the earlier periods alongside the introduction of a newtextile-lined molding technique for shaping round-bottom containers (e.g., at Tasbas, Begash, and Tamgaly). Another new forming technique of the period includes the final addition of a collared rim by attaching a ‘ribbon’ shaped coil to the inside wall of the body. Firing techniques show no notable change. The techno-stylistic features of LBA pottery repeat a well-cataloged tradition that appears throughout the eastern Eurasian steppe. In Zhetysu this period pottery in the culture histories is termed as the ‘Semirech’ye type’ and is considered a mixed tradition that combined features of Sargary-Alekseevka, Kulsai, Begazy Dandibay, Chust, and Dongal archaeological cultures [70].

### Petrographic analysis and mapping proximity to geological regions

A shared feature of all of the Bronze Age period ceramics examined is a coarse fabric texture consisting of visible and large amounts of mineral and rock inclusions. Ceramic wares do not vary between sites of the same time period, but techno-styles are noticeably different across the EBA, MBA and LBA as outlined above. Based on the high frequency of rock fragments in the ceramics, the use of petrography brings an analytical advantage for investigating technological choices in paste preparation and raw material selection over other analytical techniques. Toward this end, petrographic analysis of ceramics from the seven Bronze Age settlements was conducted to examine the petrographic and textural characteristics of ceramics under a polarized light microscope to identify raw materials and their modification such as adding tempers or sieving and cleaning the clay as well as comparing the technological variety within assemblages [71].

**Fig 5:**
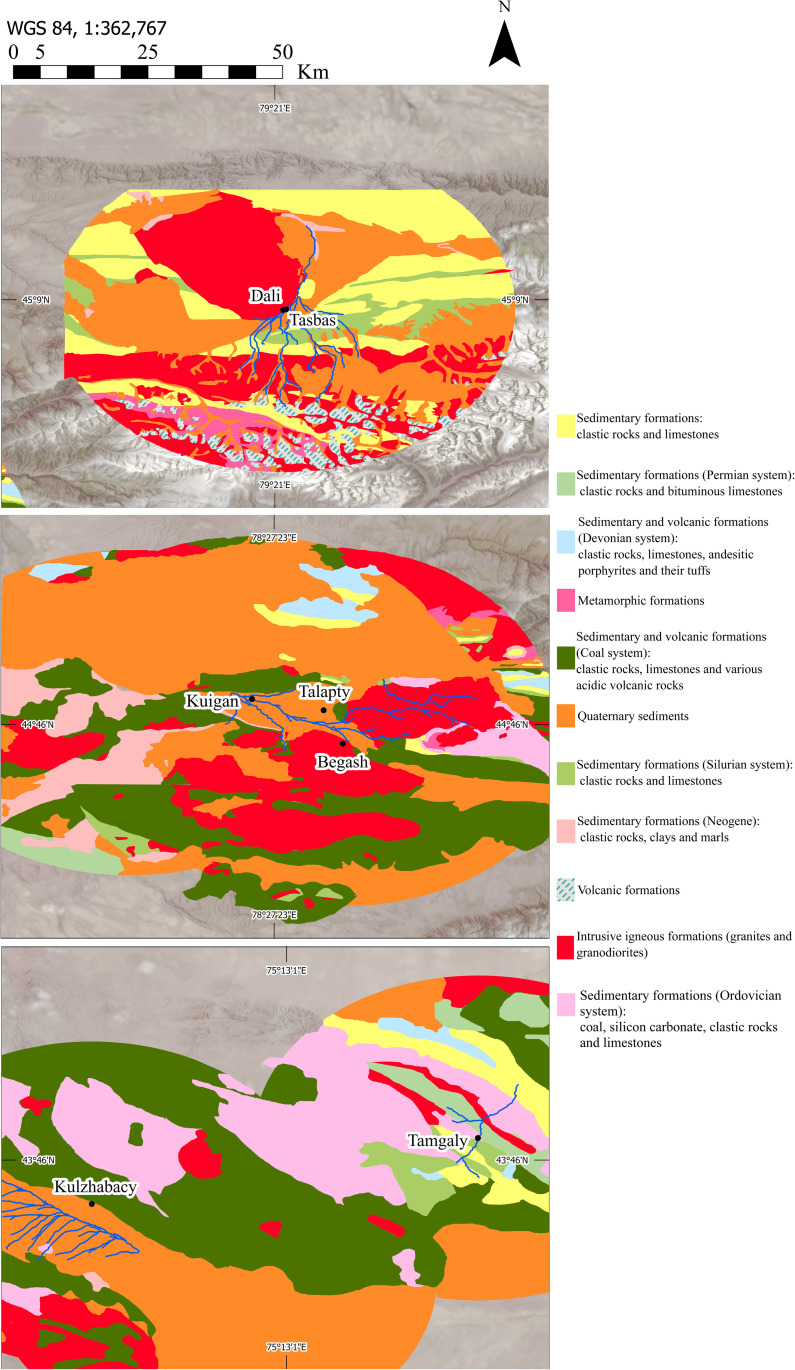
Geological map of Zhetysu, southeastern Kazakhstan with a 30 km buffer around study sites. Landsat-9 imagery and SRTM digital elevation model courtesy of the U.S. Geological Survey. Stream data courtesy of NGA TDX-Hydro dataset (https://earth-info.nga.mil/php/download.php?file=tdx-hydro-license).

Importantly, we consider clay selection including its treatment and sourcing. In order to determine the proximity of our analyzed sites to various geological deposits we also conducted informal pedestrian surveys of the valleys surrounding the study sites. We traced a vectorized map of all geological regions within a 30 km buffer of each site, using data from georeferenced Soviet geological maps of the Dzhungar, Tian Shan, and Chu-Ili Mountains with a forward and inverse RMS error of about 70 meters (Fig 5). From these digitized maps, points were generated in 50-meter intervals along the borders of geological areas of interest. Using the least-cost-paths plugin in a QGIS environment [72], we generated least cost paths between each archaeological site and these generated points. The cost surface was produced using a re-classified slope raster generated from a 1 arc-second DEM [73], such that areas of significant slope (>70 degrees) are twice as costly as other areas. Therefore, LCPs are typically straight lines with only minor adjustments around sheer topography. To account for the potential movement of geological resources along streams and watersheds, we overlay streams from the TDX-Hydro dataset (© 2023 NGA) on a geological map and measure upstream distances from each site to various rock substrates.

This spatial analysis of the geological landscape was used as a supplement to the thin section ceramic petrography. As an established analytical technique enabling one to characterize ceramic technology as well as provenance, the thin sections were analyzed with a petrographic microscope (Leica DM 2500P) equipped with a digital camera for the acquisition of photomicrographs. Given the visible differences among the samples analyzed in terms of inclusion type, size and distribution, a qualitative approach was adopted. Samples were grouped first in petrographic fabrics or petrofabrics and then described following the criteria established by Quinn [71]. All the technological interpretations, such as clay cleaning, clay mixing, tempering, and related processes, are based on criteria that are well established in the field of ceramic petrography. These criteria are extensively detailed in Quinn’s [71] seminal works, which provide comprehensive frameworks for identifying and interpreting the evidence for these practices. By adhering to these established standards, we ensure the reliability and consistency of our analyses.

## Results

**Fig 6:**
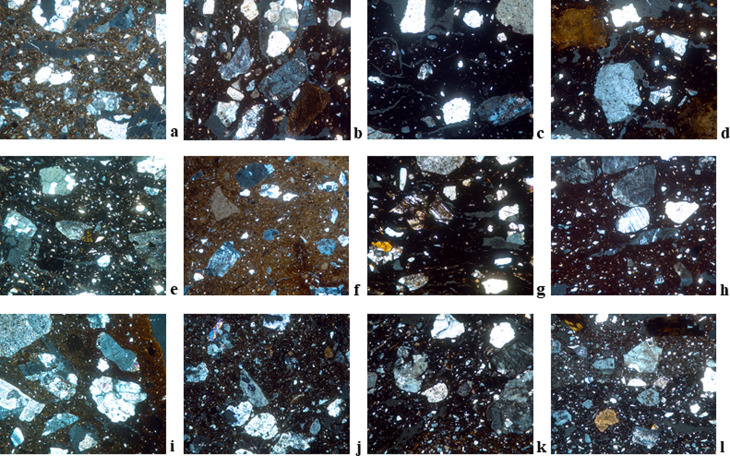
Thin section micrographs of selected Bronze Age ceramic samples from Zhetysu tempered with granite. Images were all taken with a field of view 3mm under XP. a) DL-1041, Fabric A1-DL (EBA); b) DL-0593, Fabric A2-DL (MBA); c) DL-4-05, Fabric A3-DL (LBA); d) DL-1446, Fabric B2-DL (EBA); e) TB-02C16-n/a1, Fabric A1-TB; f) BG-0544, Fabric A-BG; g) BG-0711, Fabric B2-BG; h) BG-0018, Fabric E-BG; i) KG-4, Fabric A1-KG; j) TL-5, Fabric A-TL; k) TM-4, Fabric A1-TM; l) KL-2, Fabric A1-KL.

**Fig 7:**
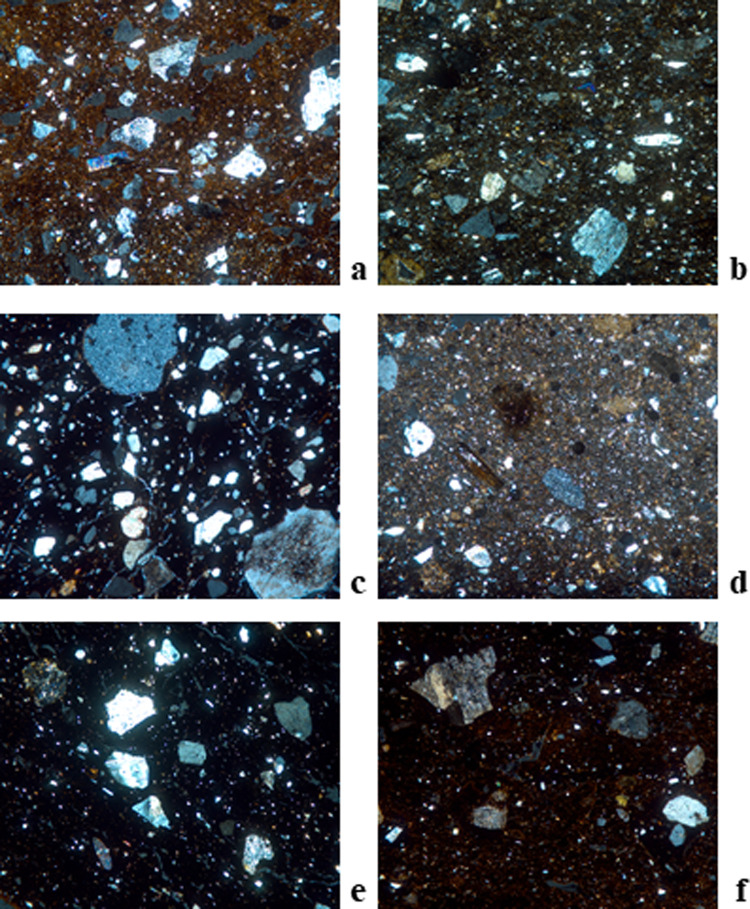
Thin section micrographs of selected Bronze Age ceramic samples from Zhetysu with polymodal distribution. Images were all taken with a field of view 3mm under XP: a) DL-4-29, Fabric C-DL; b) TB-0214, Fabric C-TB; c) BG-0769, Fabric C-BG; d) BG-0708, Fabric D-BG, e) KG-6, Fabric C-KG; f) TM-1, Fabric D-TM.

**Fig 8:**
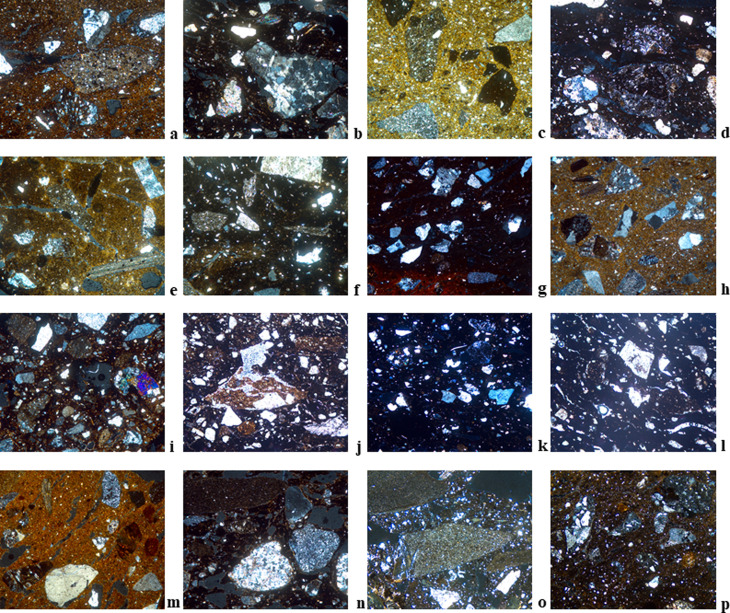
Thin section micrographs of selected Bronze Age ceramic samples from Zhetysu marked by different tempering agents. Images were all taken with a field of view 3mm under XP unless otherwise indicated. a) DL-1238, Fabric D-DL (Tempered with granite and sedimentary rock fragments); b) DL-895, Fabric E-DL (Tempered with granite and metamorphic rock fragments); c) TB-17A13-na3, Fabric B1-TB (Grog tempered); d) TB-0025, Fabric D-TB (Tempered with volcanic rock fragments); e) TB-0060-Fabric E-TB (Tempered with polymict sand); f) TB-0148, Fabric F-TB (Tempered with metamorphic rock fragments); g) BG-0990-Fabric F-BG (Tempered with sand containing granite and metamorphic rock fragments); h) BG-0067, Fabric G-BG (Tempered with sand containing metamorphic and volcanic rock fragments); i) BG-1049, Fabric H-BG (Tempered with flint and metamorphic rock fragments); j) BG-3001, Fabric I-BG (Tempered with grog) 3mm PPL; k) KG-8-Fabric B-KG (With polymict sand) Is tempered?; l) TL-2, Fabric B-TL (Grog tempered) 3mm PPL; m) TL-3, Fabric C-TL (Tempered with polymict sand); n) TM-9, Fabric B3-TM (Tempered with metamorphic rock fragments and flint); o) TM-7, Fabric C-TM (Tempered with flit) 6 mm XP; p) TM-5, Fabric E-TM (Tempered with intermediate igneous volcanic rock fragments).

A total of 141 sherds were analyzed with polarizing light microscopy (Table 1). Below we offer a summary on the main petrographic groups identified based on compositional and textural characteristics (Tables 2 and 3), while a full detailed description can be found in the supplementary materials (S1 File). The majority of the resulting fabric groups mirror the local geology, with an abundance of acidic plutonic rock fragment inclusions and deriving sediments. Alongside a preponderance for granite and other mineral-based tempers, several ‘loners’ were observed (indicated by n = 1) at all sites and with different raw materials being selected for. As a body of material, the ceramic thin-sections document bimodal fabrics with granite temper (Fig 6), polymodal fabrics with a pre-existing granite component (Fig 7), and then several smaller groups or outliers containing different raw materials or means of preparing the paste (Fig 8). Approaches to paste preparation include widespread approaches to raw material modification, such as crushing of tempers, sieving, and clay cleaning. While several of the noted trends can be observed over millennia, each site examined is recognizable for some distinctive traits, suggesting communities of practice took different scales that bridged both regional and site level arenas.

**Table 2:** Summary of the main petrographic groups identified based on compositional and textural characteristics.

Petrographic groups and subfabrics identified for each settlement in this study	Count
**Dali**	
Petrographic fabric A-DL: Granite tempered	28
• Subfabric A1-DL: Weakly bimodal-to-polymodal	(15)
• Subfabric A2-DL: Strongly bimodal with less abundant fine fraction	(11)
• Subfabric A3-DL: Strongly bimodal with well sorted fine fraction	(2)
Petrographic fabric B: Granite tempered with abundant calcite	2
• Subfabric B1-DL: Carbonate rock fragments	(1)
• Subfabric B2-DL: Abundant micrite	(1)
Petrographic fabric C-DL: Polymodal with granite	4
Petrographic fabric D-DL: Tempered with granite and sedimentary rock fragments	4
Petrographic fabric E-DL: Tempered with granite and metamorphic rock fragments	1
**Tasbas**	
Petrographic fabric A-TB: Granite tempered	21
• Subfabric A1-TB: Strongly bimodal with abundant fine fraction	(8)
• Subfabric A2-TB: Strongly bimodal with moderately abundant fine fraction	(4)
• Subfabric A3-TB: Bimodal with scarce fine fraction	(4)
• Subfabric A4-TB: Bimodal with very scarce fine fraction	(4)
• Subfabric A5-TB: Abundant biotite	(1)
Petrographic fabric B-TB: Grog tempered	7
• Subfabric B1-TB: With light matrix tempered only with grog	(2)
• Subfabric B2-TB: Granite tempered, with well sorted fine fraction	(1)
• Subfabric B3-TB: Granite tempered with moderately sorted fine fraction	(3)
• Subfabric B4-TB With fragments of metamorphic rocks	(1)
Petrographic fabric C-TB: Polymodal fabric	3
Petrographic fabric D-TB: Tempered with volcanic rock fragments	2
Petrographic fabric E-TB: Tempered with polymict sand	1
Petrographic fabric F-TB: Tempered with metamorphic rocks fragments	1
**Begash**	
Petrographic fabric A-BG: Granite tempered.	9
Petrographic fabric B-BG: Tempered with acidic-intermediate plutonic rock fragments	18
• Subfabric B1-BG: Well sorted fine fraction	(7)
• Subfabric B2-BG: Poorly sorted fine fraction.	(11)
Petrographic fabric C-BG: Dark matrix and weakly bimodal.	3
Petrographic fabric D-BG: Very weakly bimodal-to-polymodal fabric.	3
Petrographic fabric E: Well-sorted granite tempered	1
Petrographic fabric F: Tempered with sand containing granite and metamorphic rock fragments	1
Petrographic fabric G: Tempered with sand containing metamorphic and volcanic rock fragments	1
Petrographic fabric H: Tempered with flint and metamorphic rock fragments	1
Petrographic fabric I: Tempered with grog	1
**Kuigan**	
Petrographic fabric A-KG: Granite tempered	8
• Subfabric A1-KG: Bimodal	(7)
• Subfabric A2-KG: Strongly bimodal	(1)
Petrographic fabric B-KG: With polymict sand	1
Petrographic fabric C-KG: Polymodal fabric	1
**Talapty**	
Petrographic fabric A-TL: Granite tempered	3
Petrographic fabric B-TL: Grog tempered	1
Petrographic fabric C-TL: Tempered with polymict sand	1
**Tamgaly**	
Petrographic fabric A-TM: Granite tempered	2
• Subfabric A1-TM: Weakly bimodal	(1)
• Subfabric A2-TM: Strongly bimodal	(1)
Petrographic fabric B-TM: Tempered with metamorphic rock fragments and flint	5
Petrographic fabric C-TM: Tempered with flint	1
Petrographic fabric D-TM: Polymodal with calcite	1
Petrographic fabric E-TM: Tempered with intermediate igneous volcanic rock fragments	1
**Kulzhabacy**	
Petrographic fabric A-KL: Granite tempered	4
• Subfabric A1-KL: Weakly bimodal	(2)
• Subfabric A2-KL: Strongly bimodal	(2)

**Table 3:** Comparison of the petrofabric groups in diachronic and synchronic perspective.

Mountain Valley	Site	Bronze Age Phase	Granite tempered	Granite tempered w/ abundant calcite	Polymodal w/ granite	Tempered w/ granite & sedimentary rock	Tempered w/ granite & metamorphic rock	Tempered w/ acidic-intermediate plutonic rock	Grog tempered	Tempered w/ volcanic rock	Tempered w/ polymict sand	Polymodal w/ polymict sand	Tempered w/ metamorphic rocks	Tempered w/ sand containing granite & metamorphic rock	Tempered w/ sand containing metamorphic & volcanic rock	Tempered w/ flint & metamorphic rock	Tempered w/ flint	Polymodal w/ calcite	Tempered w/ intermediate igneous volcanic rock
Bayan Zhurek	Dali	Early	✓	✓	✓														
		Middle	✓	✓	✓	✓	✓												
		Late	✓		✓														
	Tasbas	Late	✓		✓				✓	✓	✓		✓						
Koksu	Begash	Early	✓					✓											
		Middle	✓					✓							✓	✓			
		Late	✓					✓	✓					✓					
	Talapty	Early/Middle	✓						✓		✓								
	Kuigan	Early	✓																
		Late	✓		✓							✓							
Chu-Ili	Tamgaly	Late	✓													✓	✓	✓	✓
	Kulzhabacy	Late	✓																

Legend. Check marks indicate the presence of a fabric group.

### Dali

#### Petrographic fabric A-DL (Fig 6a, b, c): Granite tempered (n = 28).

This fabric exhibits bimodal to weakly bimodal grain size distribution with a relatively well-sorted coarse fraction that consists primarily of rock fragments composed of quartz and feldspars (granite). Differences in the sorting of the coarse and fine fraction might reflect different ways in which the clay has been processed through more or less accurate cleaning as well as tempering with well sorted or less well sorted inclusions.

#### Petrographic fabric B-DL (Fig 6d): Granite tempered with abundant calcite (n = 2).

Like fabric group A-DL, this bimodal fabric is marked by tempering with coarse fragments of an acidic plutonic rock (granite), but it also contains a calcite in the form of micrite and carbonate rock fragments respectively.

#### Petrographic fabric C-DL (Fig 7a): Polymodal with granite (n = 4).

This fabric is characterized by a polymodal grain size distribution of minerals derived from weathering of acidic to intermediate plutonic rocks. The polymodality suggests that the inclusions might be naturally occurring.

#### Petrographic fabric D-DL (Fig 8a): Tempered with granite and sedimentary rock fragments (n = 4).

This fabric contains large fragments of clastic sedimentary rocks of diverse nature, not well sorted and often composed of quartz (arenite), together with fragments of acidic plutonic rocks (granite). The poor sorting of both the coarse and fine fraction that marks this fabric suggest that the base clay was not accurately cleaned, and the tempering agents were not well sorted.

### Dali loners

#### Petrographic fabric E-DL (Fig 8b): Tempered with granite and metamorphic rock fragments (n = 1).

This bimodal fabric represents a not well-processed clay with temper consisting of some fragments of granite and metamorphic rock fragments.

### Tasbas

#### Petrographic fabric A-TB (Fig 6e): Granite tempered (n = 21).

This fabric exhibits bimodal to weakly bimodal grain size distribution with a relatively well-sorted coarse fraction that consists primarily of rock fragments composed of quartz and feldspars (granite). Differences in the sorting of the coarse and fine fraction might reflect different ways in which the clay has been processed through more or less thorough cleaning as well as tempering with well-sorted or less well-sorted inclusions.

#### Petrographic fabric B-TB (Fig 8c): Grog tempered (n = 7).

This petrofabric group is marked by the addition of grog and is characterized by considerable compositional diversity because along with grog other tempering agents can be found such as granite (B2-TB, B3-TB), or metamorphic rock fragments (B4-TB). Differences in the sorting of the coarse and fine fraction might reflect different ways in which the clay has been processed through more or less accurate cleaning as well as tempering with well sorted or less well sorted inclusions.

#### Petrographic fabric C-TB (Fig 7b): Polymodal fabric (n = 3).

The polymodal grain size distribution of inclusions deriving from acidic plutonic rocks (granite) is the main characteristic of this fabric in which inclusions are naturally occurring.

#### Petrographic fabric D-TB (Fig 8d): Tempered with volcanic rock fragments (n = 2).

This fabric is weakly bimodal characterized by acidic volcanic inclusions added as a temper into a moderately well-cleaned clay.

## Tasbas loners

### Petrographic fabric E-TB (Fig 8e): Tempered with polymict sand (n = 1).

This loner petrofabric (TB-060) is strongly bimodal and marked by the presence of polymictic, well rounded inclusions of different types of sedimentary rocks and large feldspars and sparite. This sample might have been produced using a fluvial sediment as temper.

### Petrographic fabric F-TB (Fig 8f): Tempered with metamorphic rocks fragments (n = 1).

This fabric is bimodal and characterized by the presence of poorly sorted fragments of metamorphic rocks used as temper. The fine fraction is moderately well sorted indicating that the clay could have been cleaned prior tempering.

### Begash

#### Petrographic fabric A-BG (Fig 6f): Granite tempered (n = 9).

This fabric exhibits strong bimodal grain size distribution with a relatively well-sorted coarse fraction that consists primarily of rocks fragments composed of quartz and feldspars (granite) that display significant weathering into sericite. The bimodality and the high degree of angularity of the inclusions might indicate tempering using crushed granite rock fragments.

#### Petrographic fabric B-BG (Fig 6g): Tempered with acidic-intermediate plutonic rock fragments (n = 18).

This fabric shows strongly bimodal to weakly bimodal grain size distribution with a relatively well-sorted coarse fraction that consists primarily of rocks fragments composed of quartz, feldspars, a minor quantity of biotite, and amphiboles. Differences in the sorting of both coarse and fine fractions might reflect different ways in which the base clay has been processed through more or less accurate cleaning as well as tempering with well-sorted or less well-sorted inclusions.

#### Petrographic fabric C-BG (Fig 7c): Dark matrix and weakly bimodal (n = 3).

This fabric is compositionally similar to fabric A-BG but stands out by a weaker bimodal grain size distribution and very dark matrix. Moreover, in this fabric we cannot observe the occurrence of plutonic rock fragments (granite), but only minerals that might derive from granite weathering. In addition, rare foliated metamorphic rock fragments composed of quartz have been observed. The coarse fraction is finer in comparison to the coarse fraction in fabric A-BG and B-BG and less well sorted. The finer fraction is more abundant and coarser.

#### Petrographic fabric D-BG (Fig 7d): Very weakly bimodal-to-polymodal fabric (n = 3).

The intermediate to acidic plutonic rock fragments and derived minerals appear also in this fabric group. It is yet again set apart by the grain size distribution, which in this case is polymodal, suggesting that the inclusions are naturally occurring.

## Begash loners

### Petrographic fabric E-BG (Fig 6h): Well-sorted granite temper (n = 1).

This longer petrofabric (BG-018) displays bimodal grain size distribution and it is compositionally very similar to fabric BG-A, but the coarse fraction is better sorted, and is significantly smaller and more rounded in comparison to fabric BG-A. This might suggest a different source for the tempering material.

### Petrographic fabric F-BG (Fig 8g): Tempered with sand containing granite and metamorphic rock fragments (n = 1).

This fabric (BG-990) shows a strong bimodal grain size distribution and exhibits a very fine and well-sorted fine fraction, that could indicate the use of a very fine clay or cleaning of clay via levigation. The coarse fraction is well-sorted and consists of rounded acidic plutonic (granite) and metamorphic rock fragments that could have been added as temper. The roundness of the inclusions together with their polymict nature of the coarse fraction might suggest the use of a fluvial sediment for tempering.

### Petrographic fabric G-BG (Fig 8h): Tempered with sand containing metamorphic and volcanic rock fragments (n = 1).

This longer fabric (BG-067) is strongly bimodal with a very well-sorted fine and coarse fraction. This implies the use of a very fine clay or an accurate cleaning of clay. The presence of metamorphic and volcanic rock fragments makes this fabric different in comparison to other ceramics from Begash. The roundness of the inclusions together with the polymict nature of the coarse fraction might suggest the use of a fluvial sediment for tempering.

### Petrographic fabric H-BG (Fig 8i): Tempered with flint and metamorphic rock fragments (n = 1).

This loner fabric (BG-1049) is weakly bimodal and marked by big and not well sorted fragments of metamorphic rocks alongside flint and few fragments of acidic plutonic rocks (granite). The poor sorting and weak bimodality that mark this fabric suggest that the base clay was not well-cleaned and the tempering agented were not well-sorted.

### Petrographic fabric I-BG (Fig 8j): Tempered with grog (n = 1).

The presence of grog inclusions in this fabric (BG 3001) separates it from other fabrics from Begash. The polymodal grain size distribution points towards the addition of grog to an uncleaned clay.

### Kuigan

#### Petrographic fabric A-KG (Fig 6i): Granite tempered (n = 8).

This fabric includes bimodal to weakly bimodal samples marked by the presence of acidic plutonic rock fragments (granite) used as temper. Differences in the sorting of the coarse and fine fraction might reflect different ways in which the clay has been processed through more or less accurate cleaning as well as tempering with well-sorted or less well-sorted inclusions.

### Kuigan loners

#### Petrographic fabric B-KG (Fig 8k): With polymict sand (n = 1).

This loner (KG-8) is marked by rounded inclusions of diverse nature including plutonic, volcanic, and metamorphic rock fragments. The polymodality and diverse nature of the inclusions with rounded grains could suggest the use of a fluvial clay as raw material to produce the ceramic.

#### Petrographic fabric C-KG (Fig 7e): Polymodal fabric (n = 1).

This loner (KG-6) is marked by the presence of feldspars and quartz. Rarely acidic plutonic rock fragments (granite) can be observed. The polymodal grain size distribution indicates the inclusions are naturally occurring.

### Talapty

#### Petrographic fabric A-TL (Fig 6j): Granite tempered (n = 3).

This fabric is characterized by sparse acidic plutonic rock (granite) fragments composed of quartz and feldspars. The bimodal grain size distribution indicates that granite rock fragments might have been used as a tempering agent. The poor sorting of both the coarse and fine fraction that marks this fabric suggest that the base clay was not well-cleaned, and the tempering agents were not well-sorted.

### Talapty Loners

#### Petrographic fabric B-TL (Fig 8l): Grog tempered (n = 1).

This loner fabric (TL-2) is compositionally similar to fabric group A-TL, but the distinguishing factor is the addition of grog. The poor sorting of the inclusions might indicate that the base clay was not well-cleaned before the grog was added.

#### Petrographic fabric C-TL (Fig 8m): Tempered with polymict sand (n = 1).

This loner fabric (TL-3) is strongly bimodal and characterized by well-rounded inclusions of metamorphic, plutonic and volcanic rocks. The strong bimodality as well as the roundness and polymictic nature of the coarse fraction might suggest that fluvial clay as a tempering agent was added to a well cleaned clay.

### Tamgaly

#### Petrographic fabric A-TM (Fig 6k): Granite tempered (n = 2).

This fabric includes bimodal to weakly bimodal samples marked by the presence of acidic plutonic rock fragments (granite) as temper. Differences in the sorting of the coarse and fine fraction might reflect different ways in which the clay has been processed through more or less accurate cleaning as well as tempering with well-sorted or less well-sorted inclusions.

#### Petrographic fabric B-TM (Fig 8n): Tempered with metamorphic rock fragments and flint (n = 5).

This strongly bimodal fabric is marked by foliated metamorphic rock fragments containing quartz as well as muscovite and flint that might have been added as temper to a relatively well-cleaned clay.

### Tamgaly Loners

#### Petrographic fabric C-TM: Tempered with flint (Fig 8o) (n = 1).

This loner fabric (TM-7) is bimodal and marked by the presence of frequent flint inclusions, very few rock fragments of foliated metamorphic rocks as well as rare basic porphyritic volcanic rock fragments were observed in this fabric. This heterogenous sand might have been added as temper to a not well-cleaned clay as indicated by the poor sorting of the latter.

#### Petrographic fabric D-TM (Fig 7f): Polymodal with calcite (n = 1).

This loner fabric (TM-1) is polymodal and characterized by the presence of inclusion that might derive from the weathering of acidic plutonic rocks (granite) and calcite. The polymodality might indicate that the inclusions are naturally occurring.

#### Petrographic fabric E-TM (Fig 8p): Tempered with intermediate igneous volcanic rocks fragments (n = 1).

This loner fabric is strongly bimodal and marked by intermediate igneous volcanic rock fragments that have been added as temper to a moderately well-cleaned clay.

### Kulzhabacy

#### Petrographic fabric A-KL (Fig 6l): Granite tempered (n = 4).

This fabric includes bimodal to weakly bimodal samples marked by the presence of acidic plutonic rock fragments (granite) used as temper. Differences in the sorting of the coarse and fine fraction might reflect different ways in which the clay has been processed through more or less accurate cleaning as well as tempering with well-sorted or less well-sorted inclusions.

## Discussion: Space-time dynamics of Bronze Age potting

The ceramic petrographic study focused on materials from seven Bronze Age agropastoral settlements in the Zhetysu region of southeastern Kazakhstan with occupation histories spanning ca. 2700–800 cal BC. Petrographic and textural characteristics of ceramics were examined under a polarized light microscope to identify long-term approaches to clay and temper collection, and subsequent crushing, sorting, cleaning and paste mixing in highland, midland and arid foothill zones of Zhetysu. Our results show that core craft technology traditions persisted in the region across and throughout episodic stylistic changes and across periods of widespread material-social transitions in Eurasia coinciding with substantial shifts in human biological admixture. We further recognize that within a local ceramic industry, some vessels or materials may have circulated starting during the 2nd millennium BC. Finally, we note a long-term preference for granite as a tempering material when alternative choices were closer or would have worked just as well, thus showing granite to be a geological landmark for the region’s pottery. The three main patterns we observe together highlight the combined contribution of mountain transhumance, enduring tradition, and cultural choice to the materiality of Bronze Age agropastoralists.

### Breaking and building traditions: Pottery technology and style

From the 39 thin-sections analyzed from the Bayan Zhurek valley settlement of Dali we detect just five petrofabric groups across the early to late Bronze Age. Overall, we note considerable long-term homogeneity in the Dali ceramics; >70% of samples belong to a single petrofabric group, and then among all groups, granite inclusions are present usually in the form of temper (Fig 6: a-d), and infrequently as naturally occurring mineral inclusions (Fig 7: a). When tempered, rocks were crushed and then uniformly or moderately sorted for size and added to fine or cleaned clays. A technological preference for granite and similar ways of processing it appear in every occupation period at Dali. Notwithstanding, we observe at MBA Dali a greater diversity of choices in the technical repertoire for paste mixing and recipes as reflected in the presence of sedimentary and metamorphic rocks in ~25% of the sample (Fig 8: a, b). Subsequently, LBA pottery from Tasbas shows even greater technological variability. Its 35 thin-sections divide into six petrofabric groups. While the majority are tempered solely with granite, we classify 40% of the bulk sample as outliers because they show an entirely different approach to paste preparation (Fig 8: c-f). Ultimately, the overriding diagnostic feature of the Tasbas pottery is raw material variability: tempering agents include sand, granite, volcanic, metamorphic and sedimentary rocks, as well as grog. The introduction of grog temper here marks an active departure in the core operational steps of paste preparation used earlier on at its neighbor Dali. The Bayan Zhurek valley ceramics represent the earliest known traditions of paste preparation and mixing in the study zone, and those traditions are shown to endure for nearly 2000 years alongside the incorporation of new methods that produced greater technological variability through time.

Ceramics from the Koksu Valley share some features observed in ceramics from the Bayan Zhurek valley settlements to their north. The 38 analyzed thin-sections from Begash are classified into four petrographic groups that account for >85% of its analyzed assemblage spanning the early to late Bronze Age. Those four groups all contain granite temper, but minor variations in texture and grain size distribution possibly indicate clay cleaning, size sorting, and temper crushing were approached in different ways during the site’s history or at the community level (Fig 6f-h and 7c,d). By the LBA, some level of technological variation was certainly in place at Begash as shown by five outlying samples containing metamorphic and volcanic rocks, flint, sand and/or grog (Fig 8g-j). At the other analyzed Koksu valley sites of Kuigan 1 and 2 (LBA and EBA) and Talapty (MBA), granite temper also predominates where it appears in 80% of the ceramics examined. At Kuigan granite is unanimously present (Fig 6i), but at Talapty we also detect the use of grog and polymict sand with metamorphic, plutonic and volcanic rock fragments (Fig 8l, m). Collectively the thin-section data from the Koksu demonstrates an overriding preference for granite tempered pottery. Long-term retention in technological traditions is accompanied by new approaches over time, such as for instance the addition of grog to ceramic pastes. However, pottery thin-sections for the Koksu Valley sites reveal a different array of raw materials that suggest localized use of the geological landscape.

Lastly, in the Chu-Ili Valley situated in the southwestern corner of Zhetysu, vessels that are outwardly similar and from the LBA reveal multiple distinct petrofabrics. The most notable feature is that within the Tamgaly sample (n = 10) granite tempered pottery makes up just 20%, whereas the other 80% of the sample contains tempers from flint, volcanic and/or metamorphic rock (Fig 8n-p). In comparison to the heterogeneity observed in the Tamgaly ceramics, at Kulzhabacy all observed thin-sections (n = 4) are granite tempered (Fig 6). The Chu-Ili pottery shows a wider exploitation of the natural environment whereby materials were gathered from a broader variety of mineral resources than observed for the Koksu and Bayan Zhurek Valleys to the north. Such a focus on raw material procurement and ceramic paste preparation in synchronic and diachronic perspectives bring into view the technological choices of potters across an array of mountain environments.

**Fig 9:**
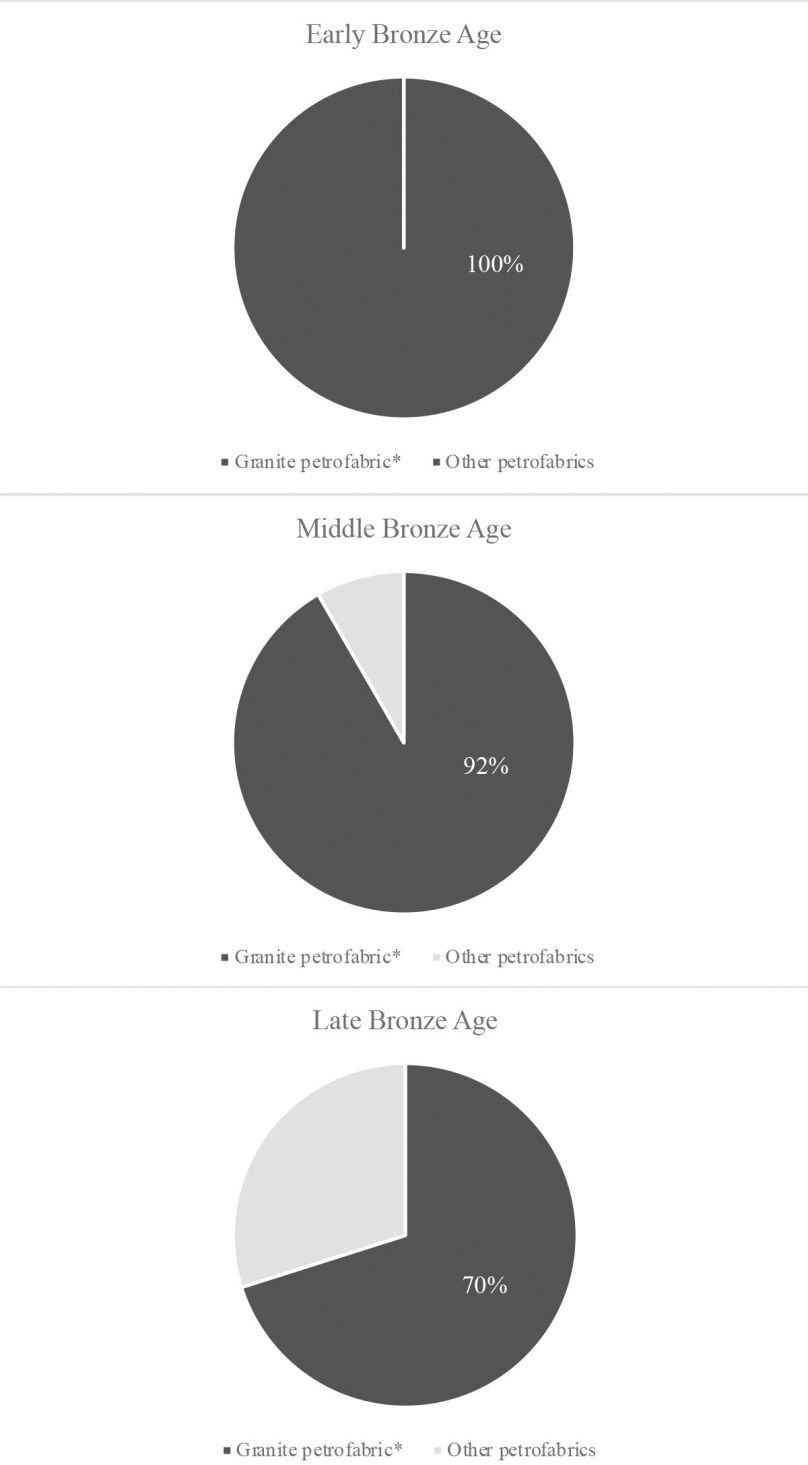
Presence of granite petrofabrics versus non-granite petrofabrics for all sites for the EBA, MBA and LBA.

Certainly, Zhetysu ceramics reflect remarkably long-lasting technological traditions that are learned through observation and practice and hence dependent on high levels of social integration and knowledge transmission across space and time. Ceramics assigned to the EBA from Dali, Begash and Kuigan derive from similar approaches to paste preparation that continue into later periods; their potteries are all granite tempered (e.g., Fig 6) or else show untempered fabrics in which granite fragments are naturally occurring (Fig 7). As the 2^nd^ millennium BC unfolds, new types of pastes appear as shown by the greater number of petrofabric groups documented at Dali and Talapty (e.g., Fig 8). Andronovo Culture movements during the MBA might account for the observed technological diversification. At the LBA, a period of additional population interaction, even more paste mixing techniques and tempering materials become part of the potter’s repertoire, which we plainly see at Tamgaly and Tasbas (e.g., Fig 8). From a diachronic perspective, we observe a trend in Zhetysu whereby antecedent knowledge of communities of practice survive alongside growing diversity in potting technologies (Fig 9). In parallel to the technological changes observed, those highly visible attributes of ceramic style that can signal group identity, change with each new Bronze Age epoch; egg-shaped pots of the EBA contrast with the various iterations of MBA open jars hosting geometric patterns, and then again with the LBA round-bellied pots and their minimal ornamentation. These stylistic transitions chart some amount of demographic change including new people - and potting communities - moving into the area. Nevertheless, results of the thin section study demonstrate that the foundational steps of potting already present in the region were retained in the memory and practices of later populations. Ultimately, the core approach to paste preparation survives across and throughout several generations and across periods of material-social transition. As such, an environmental perspective cannot account for all the patterns we uncovered.

### Granite: An ecological landmark of Zhetysu

The use of granite temper or residual clays where granite fragments are naturally occurring account for the majority or a proportion of the ceramics at every settlement included in this study. The prevalence for granite in the Zhetysu ceramics underscores the impact of tradition and cultural choice in the materiality of Bronze Age transhumance. From a landscape perspective, we are working within limitations; the granite dominant geology of the study region means that we cannot pinpoint a specific source location without detailed geological surveys. Notwithstanding, the ubiquity of igneous formations throughout Zhetysu direct us to the minimal effort principle, which considers the amount of energy people generally invest to obtain raw materials [52]. This economic perspective may help explain why granite tempering remained so prevalent for nearly 2000 years across cumulative social change and interaction. Our Least Cost Path Analysis (LCPA) of published geological maps yield the proximity of each analyzed site to its nearest intrusive igneous source (Table 4, Fig 5). Dali, Tasbas and Begash are located within granitic zones, and the remaining range up to approximately 12 kilometers away. In the Bayan Zhurek Valley, granite outcrops surround the sites of Dali and Tasbas where ceramics were found to be mostly granite tempered. The geological setting of the Bayan Zhurek valley may account for why granite predominates in the ceramics of Dali and Tasbas, and therefore supports an explanation grounded in cultural ecology. However, the geological setting of the Koksu Valley, where the sites of Begash, Kuigan and Talapty are located, is quite dissimilar and predominated by sedimentary and volcanic formations, and quaternary sediments. Begash sits at the junction of those formations and an intrusive igneous (granite) formation. Potters at all three Koksu Valley sites still select for granite despite their location in a mixed geological landscape that offered several different options of raw material. The minimal effort principle [52] does not explain the identified pattern for the Koksu valley but rather strengthens the likelihood of their being social-cultural motivations to exploit granite sources. For the Chu-Ili settlements of Kulzhabacy and Tamgaly, we encounter other challenges to overriding economic interpretations. Kulzhabacy sits among mostly sedimentary formations, whereas all of its analyzed ceramics are granite tempered with the closest granite outcrops located well over 10 km away. In contrast, granite sources were within walking distance of the settlement of Tamgaly, but the majority of its pottery shows volcanic and metamorphic rock temper. The Chu-Ili ceramics give some indication for ceramic circulation, craftspeople mobility, as well as potters exploiting numerous geological landscapes. The noted technological variability in the Tamgaly ceramics may be associated with the site’s ritual importance wherein different groups were likely drawn there at different times [61]. Returning to our focus on raw material choice that often included granite, it is clear that there was a social component to the patterns observed. The Koksu and Chu-Ili data demonstrate that communities maintained an overriding preference for particular, and more varied, raw materials even when others were more proximal that may have worked just as well. People traversed multiple kilometers through rugged mountain terrain and appear to have exploited certain culturally preferred environments while bypassing others. While possible that granite-tempered ceramics of Zhetysu created a functionally superior product in some way, the repeated and long-term choices being made throughout this region starting from the EBA are likely also the materialization of a tradition transmitted from generation to generation that developed through a constant dialogue between people and their landscape, even as new communities settled in the region. A combined environmental and social perspective can similarly account for the combination of local and potential geographically circulated wares we observe in Zhetysu.

**Table 4:** Least Cost Path Analysis between granite sources and study sites.

Site	Distance to intrusive igneous source (km)	Petrofabric groups containing granite (temper or naturally occurring granite) among all petrofabric groups	Petrofabric groups with other mineral (or grog) tempers
**Dali**	0	5 out of 5	1 out of 5
**Tasbas**	0	3 out of 5	4 out of 5
**Begash**	0	7 out of 9	5 out of 9
**Kuigan**	6.89	2 out of 3	1 out of 3
**Talapty**	3.30	1 out of 3	2 out of 3
**Tamgaly**	2.54	2 out of 5	3 out of 5
**Kulzhabacy**	14.13	1 out of 1	0 out of 5

Legend. Distance in kilometers between study sites and igneous sources as obtained from LCPA; frequency of granite and/or other raw materials out the total number of ceramic petrofabrics identified for each site.

### Archaeology of transhumance: Material culture mobility

A small number of wheel-made ‘imports’ with techno-styles linking them to later Bactria-Margiana societies of southern Central Asia are a regular component of pottery assemblages in the IAMC and Eurasian steppe [18]. In Zhetysu, examples are found at Begash and Tamgaly, among other sites as judged based on formal assessments of their shape and texture. Fineware ‘imports’ are visually distinct from the handmade coarsewares typical of agropastoral settlements in Zhetysu. Beyond this discrete body of ceramics, we are limited in our means to comment on the extent to which the coarseware pottery that is examined here was circulated or part of exchange and trade within Zhetysu

For the EBA, the thin-section data does not offer conclusive evidence for the geological origins of tempers/clays because all the ceramics contain materials found within reasonable walking distance as well as several tens of kilometers away from their parent settlement - hence casting quite a large net of possibility. By contrast, for the 2nd millennium BC, we observe greater diversity in raw materials that could indicate ceramic mobility or possibly different uses of the landscape. The LCPA conducted on the geological maps reveal that extensive distances divide the settlements from raw material sources identified among some of our MBA-LBA samples (S1 Table). The LCPA measures distances to some of the identified raw material sources to be 5–25 km away from some of our sites (Fig 5). For each site we also measured distances along rivers to upstream rock substrates and took account of which rock types are absent from these hydrological basins (TDX-Hydro dataset). From this, we conclude that raw materials do not appear to be washing downstream where potters could have collected them from nearby riverbeds. Employing pack animals would certainly allow people to cover more ground, but our calculations still fall on the conservative side given the mountainous terrain. Moreover, domesticated horses were not yet available in this region until about 2000 BCE, as known from comprehensive paleogenomic research [74], in addition to zooarchaeological research [75]. It is possible that groups may have on occasion moved with their pottery and/or obtained raw materials from regions distant to their home range. In the future, more environmental studies of the region that include detailed geological surveys [76,77] will help to confirm one way or the other the nature and prevalence of ceramic mobility throughout Zhetysu’s Bronze Age.

At present we draw attention to the observation outlined in the culture-histories that potters of the different periods were making containers that fit more or less the same visual template over large geographic areas. If ceramics were occasionally circulated to source their internal contents or simply the container, we find it unlikely that they were the target commodity as was the case for ores and metal objects [e.g., 78,79]. Comparable pottery studies conducted outside of Zhetysu offer grounds for potential explanations. Womack [54] employing the concept of *translocality* argues that ceramic mobility aided the maintenance of kinship ties and group identity across extended social landscapes in Bronze age China. In a related vein, Kuzmina [70: 48] in her early work on Zhetysu presented a hypothesis that has been largely overlooked in later scholarship wherein she proposed that MBA (Andronovo) ceramics could be understood within the framework of social/marriage alliances between incoming and pre-settled groups. Consideration of the broader social landscape of the Bronze Age takes us part of the way in being able to bring into view the people behind the pottery.

Accordingly, Santacreu [80:134] in his interrogation of environmentally-centered explanations for raw material exploitation and ceramic mobility emphasizes the broader range of activities that might account for the distances people cover and their overall movements through any given landscape. This observation highlights that raw material exploitation and whereabouts do not solely direct ceramic technology. In southeastern Kazakhstan’s Zhetysu, mountains formed the *taskscape* [81] for a range of mobile activities and beliefs systems including seasonal transhumance, animal management, dwelling construction, ritual and social meeting places, and to mark out cemeteries for the deceased [60,82–84:7–9]. This taskscape encapsulated distances anywhere from just a few kilometers to 50km, or greater. How did such a system affect ceramic traditions over the long term? The results of our pottery study suggest a process whereby societies interacted and exploited the same landscapes toward multiple ends. Such a process opened the way for a diversity of raw material deposits within a given catchment zone to enter into ceramics manufacture, possibly for containers to also be circulated, and for a wide array of materials and knowledge to be built into the potter’s tool kit and be transmitted to the next generations of potters. Models currently used in discussions of ceramic technology and mobility among mobile hunter-gatherers [34,35], or small-scale agricultural groups [52] cannot be applied to the Bronze Age potters of Zhetysu. Rather, the technological and stylistic choices of potters uncovered for Bronze Age Zhetysu reflect the flexibility and adaptability of Eurasian transhumance in agropastoral systems.

## Conclusion

This study contributes the first published research on ceramic petrography for Bronze Age southeastern Kazakhstan, and we hope that other scholars will build on our initial findings in the future. Several questions emerge from the preliminary findings, such as, from where and when did the initial knowledge for pottery making within the studied mountains arrive? Why were traditions retained as new ideas were incorporated? What factors account for the differences we see across the multiple sites examined? For the moment, we offer preliminary answers to these questions that also invite future comparative studies. For Zhetysu the identified patterns from the ceramic petrography reflect relatively tight learning networks and high levels of social integration among communities where ‘function’ or ‘proximity’ may not be the ultimate deciding factors underlying potters’ behaviors. The retention of earlier ways were not necessarily conscious choices, but they do offer an indication that regional interactions during its Bronze Age did not create turnovers in the population to the extent that the materiality of community dynamics were interrupted or lost. In southeastern Kazakhstan’s Zhetysu, pottery cultural histories connect changes in ceramic form and decoration at the MBA and then again, at the LBA to the influx of new populations who mixed with pre-settled inhabitants. These ideas have undergone considerable interrogation and critique since they were initially proposed, but today scholars agree that these periods in central Eurasia were dynamic, often populations mixed and shared genes [37,39], and at other times groups remained genetically isolated amidst adoption of new cultural practices [85,86]. It appears for Zhetysu both inward and outward dynamics may have contributed to the material landscape observed through our analysis.

Bronze Age ceramics of Zhetysu demonstrate an economic and technological network embedded in an economic system of transhumance that operated on the two major principles. The first being enduring environmental knowledge that implies quite a high level of long-term social integration among agropastoralists of this area. The second being cultural choice, further underscores the socially dynamic nature of this portion of the IAMC, including the potential for ceramic circulation. The pottery tradition of Zhetysu, for now at least, can be located to start at Dali in the early 3^rd^ millennium BC. While Dali’s pottery is by no means ‘early’ compared to surrounding areas, currently the earliest pottery in Zhetysu is from the Dali settlement thus leaving open the question of technological antecedents. At present we can point out that the granite fabric tradition emerged with Dali and communities continued to use it. In fact, throughout the Bronze Age potteries underwent minimal technological change, and we observed no indication for extensive experimentation with other ceramic technologies until the mid-2^nd^ millennium BC. Even so, during later periods there is insufficient observable variation in paste recipes across the multi-settlement dataset that would indicate social divisions had developed throughout this mountain landscape. Our study revealing unity and diversity in crafting practices demonstrates that material technological research is essential for uncovering deep-histories of human-environment interactions at dynamic periods of prehistory. Our results illuminate how and to what degree technological adaptations connected people in the Bronze Age beyond social signals as seen from stylistic-typological studies.

## Supporting information

S1 Table Sample list and descriptions of ceramics included in petrographic study on Bronze Age Zhetysu. (XLSX)

S1 Fig Photographs and illustrations of ceramics included in petrographic study on Bronze Age Zhetysu. (PDF)

S1 File Results of petrographic analysis of Zhetysu Bronze Age pottery, southeast Kazakhstan. (DOCX)
