# Supplementary material for: Rocks and clay: Potters’ technological choices within the cultural dynamics of Bronze Age Kazakhstan
Source: PLoS One. 2025 Apr 23;20(4):e0320140. doi: 10.1371/journal.pone.0320140 (PMC12017490; doi:10.1371/journal.pone.0320140)

Supplementary File: S1 Figures.

Photographs and illustrations of ceramics included in petrographic study

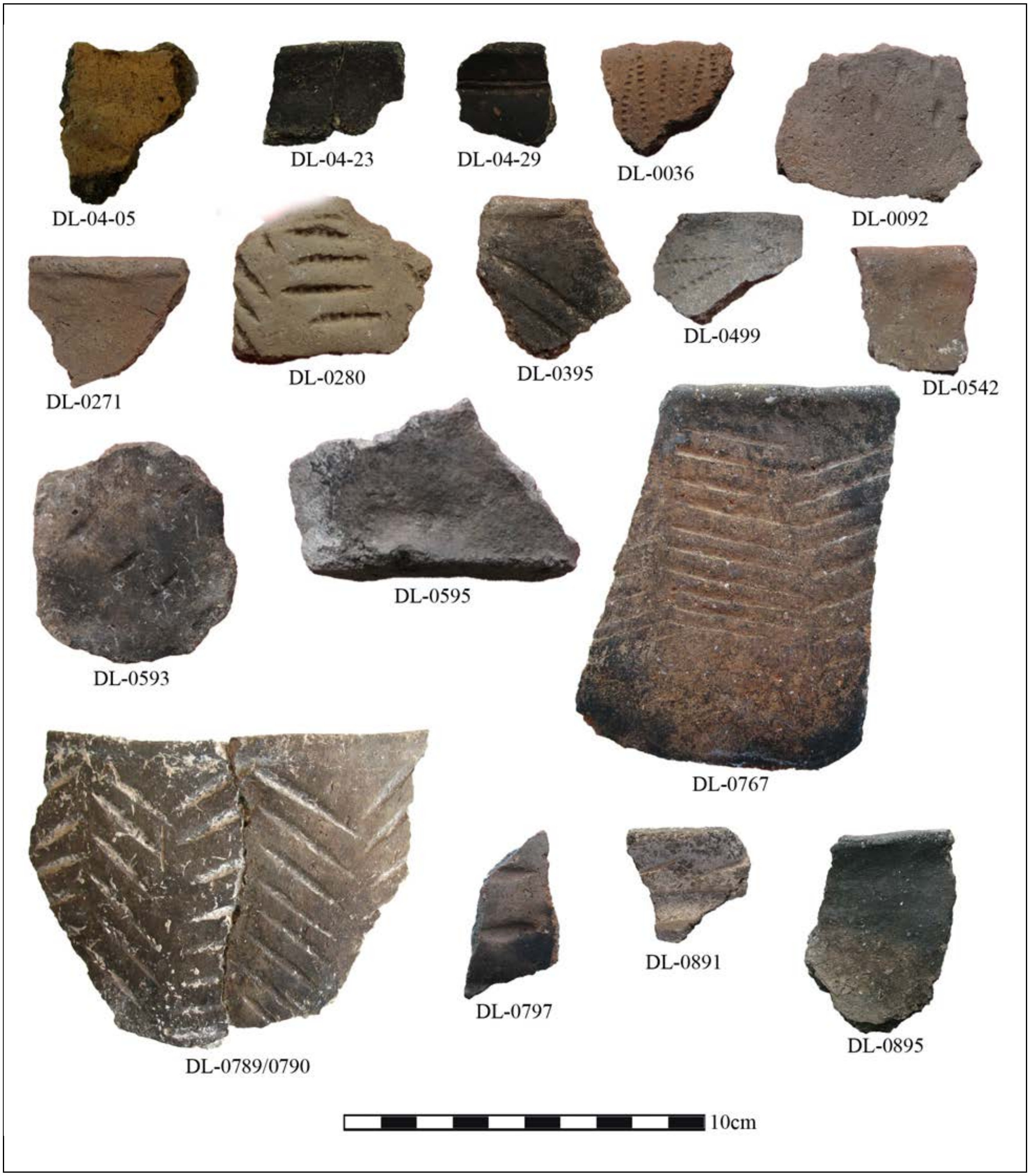

S1 Fig 1. Ceramic samples from Dali that are included in the petrographic study. Labels refer to sample IDs that are also listed in S1 Table. Any ceramics shown in the main text Fig 6-8 are not duplicated here.

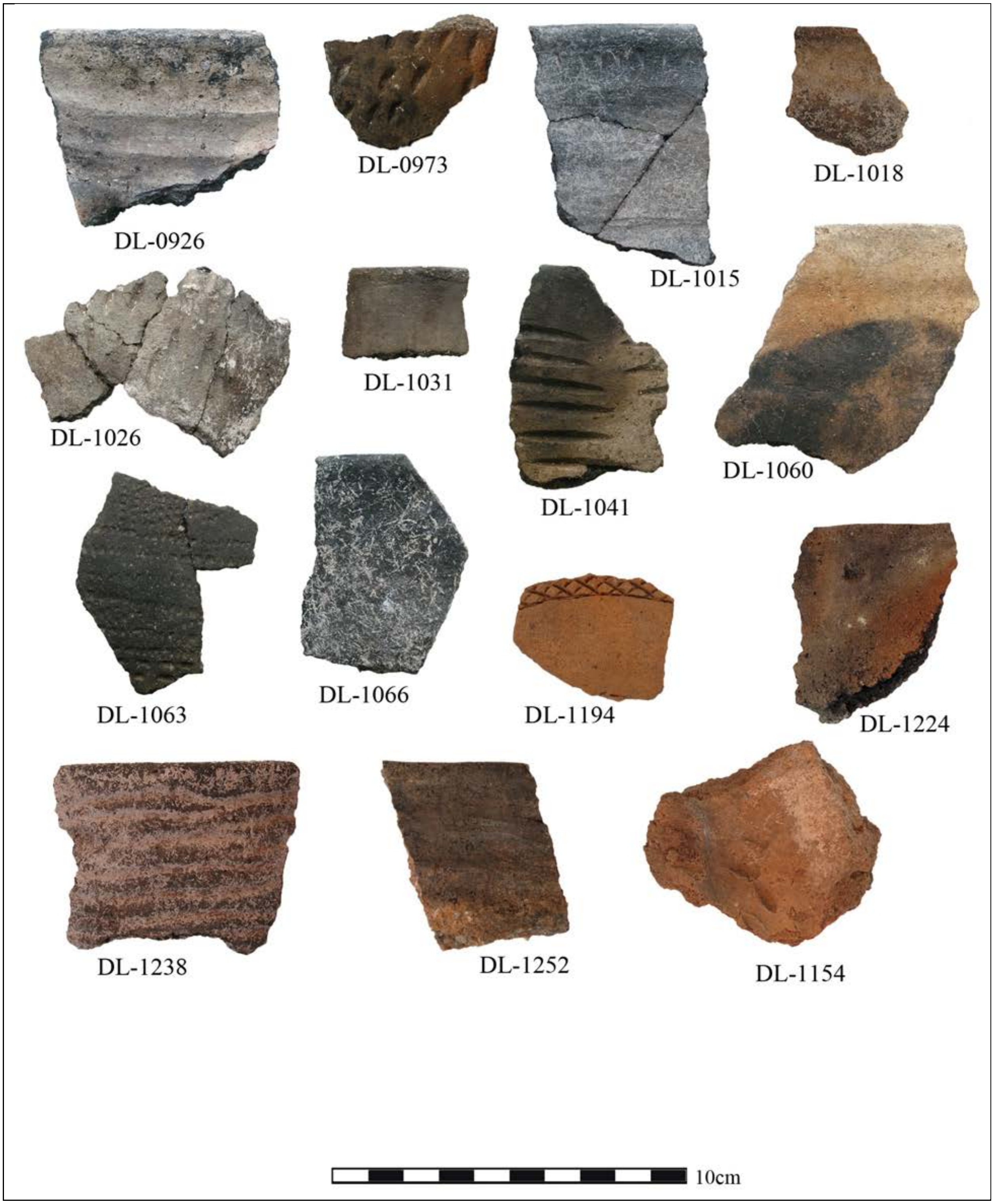

S1 Fig 2. Ceramic samples from Dali that are included in the petrographic study. Labels refer to sample IDs that are also listed in S1 Table. Any ceramics shown in the main text Fig 6-8 are not duplicated here.

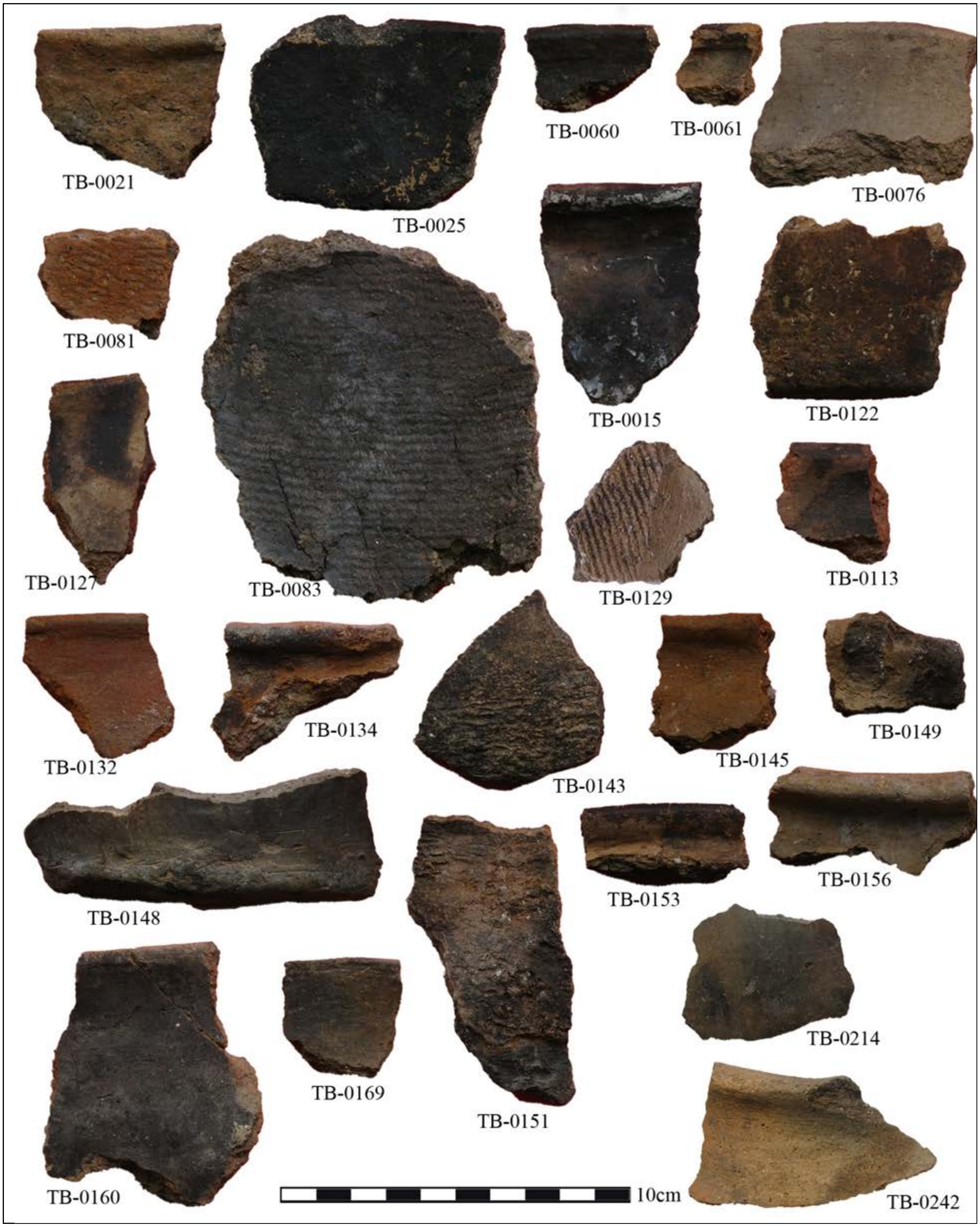

S1 Fig 3. Ceramic samples from Tasbas that are included in the petrographic study. Labels refer to sample IDs that are also listed in S1 Table. Any ceramics shown in the main text Fig 6-8 are not duplicated here.

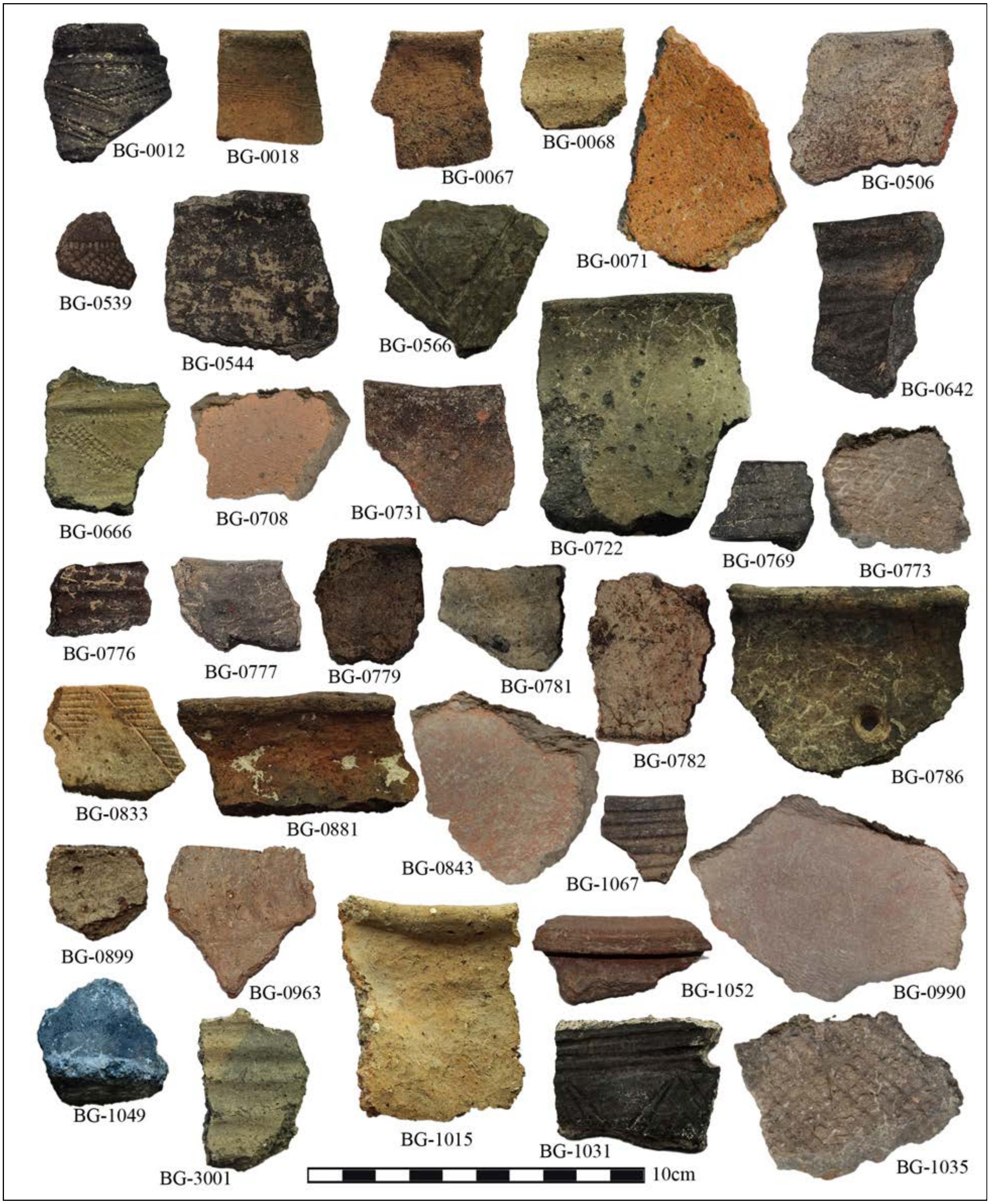

S1 Fig 4. Ceramic samples from Begash that are included in the petrographic study. Labels refer to sample IDs that are also listed in S1 Table. Any ceramics shown in the main text Fig 6-8 are not duplicated here.

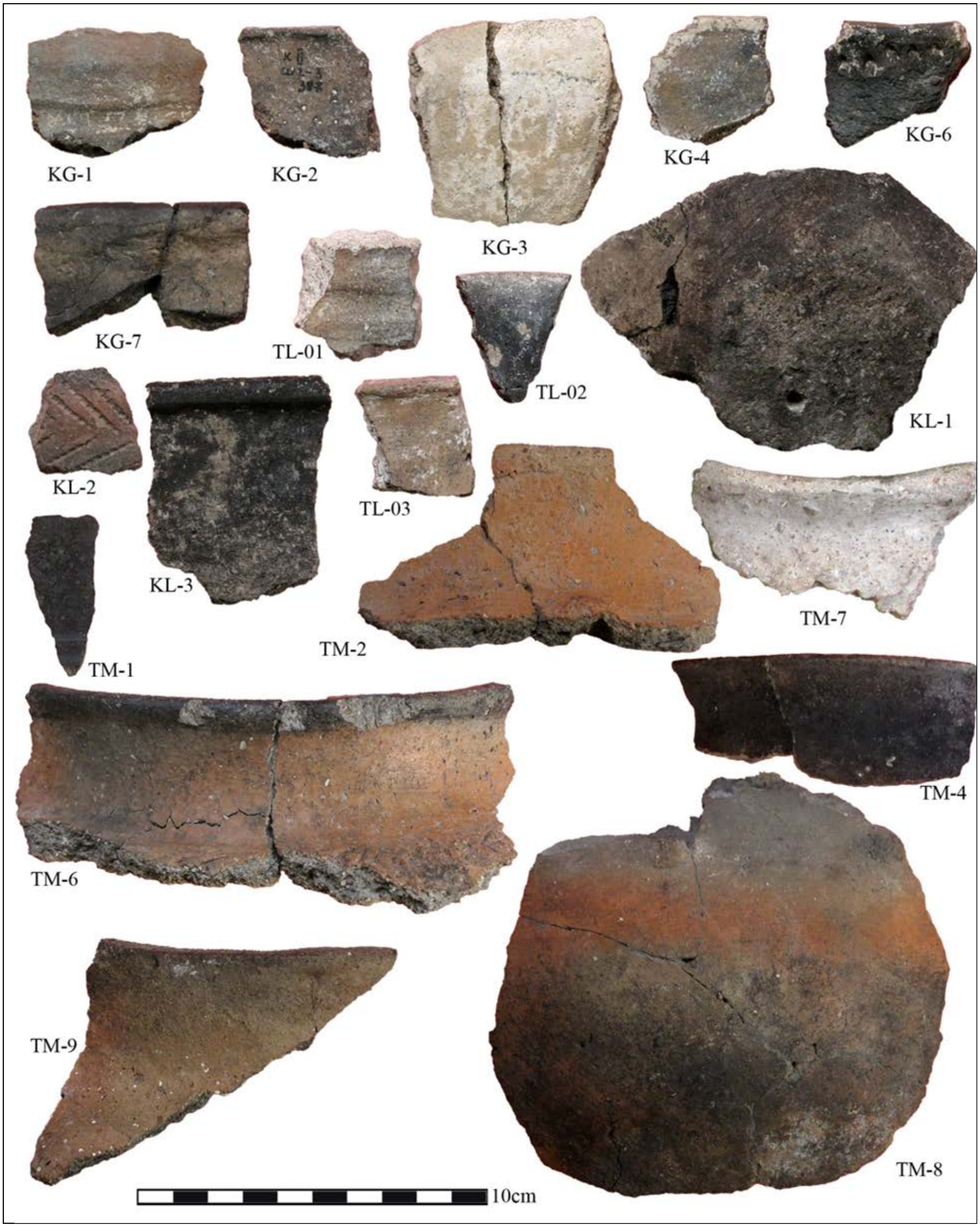

S1 Fig 5. Ceramic samples from Kuigan, Talapty, Tamgaly and Kuljabacy that are included in the petrographic study. Labels refer to sample IDs that are also listed in S1 Table. Any ceramics shown in the main text Fig 6-8 are not duplicated here.

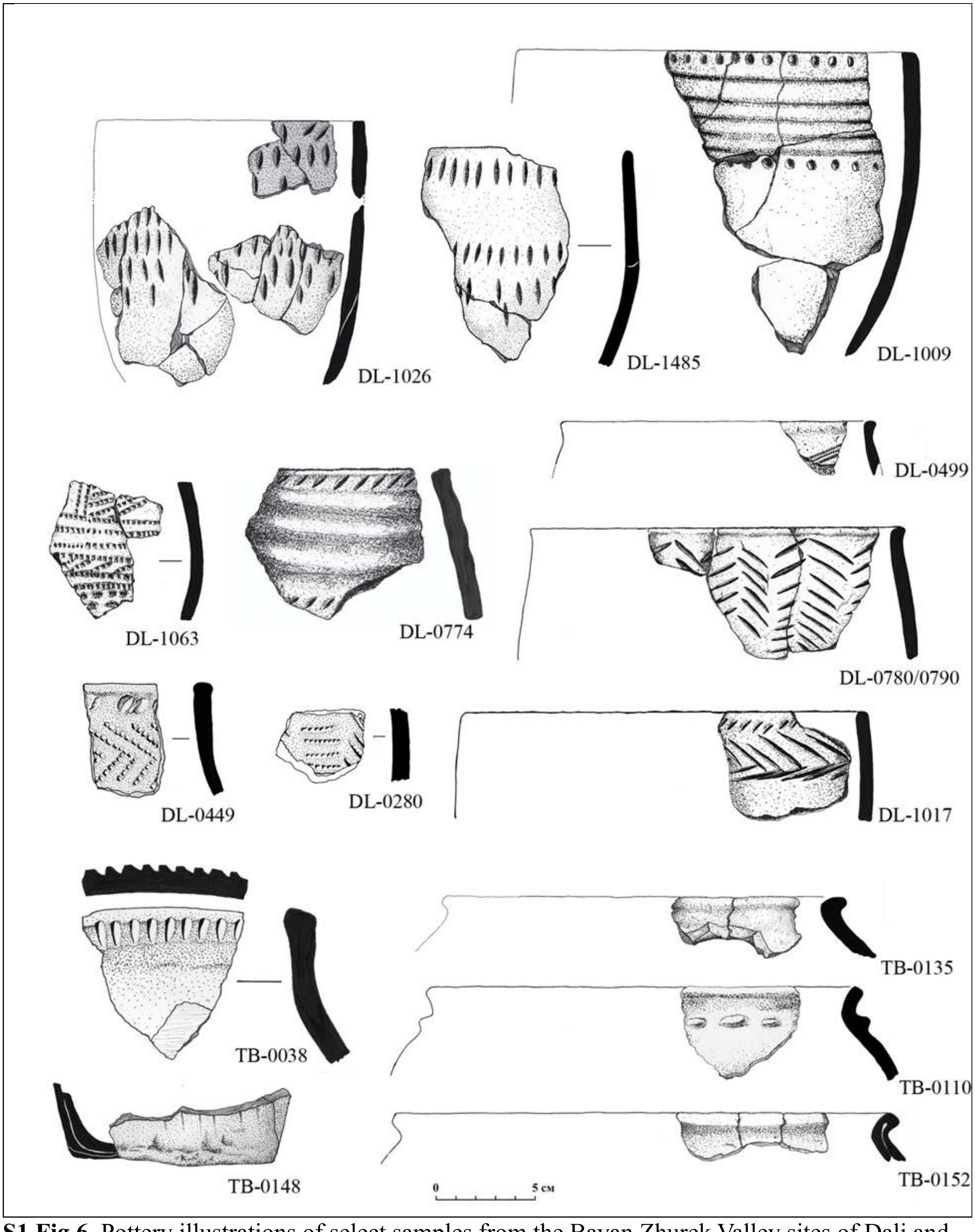

S1 Fig 6. Pottery illustrations of select samples from the Bayan Zhurek Valley sites of Dali and Tasbas in southeastern Kazakhstan. Labels refer to sample IDs that are also listed in S1 Table.

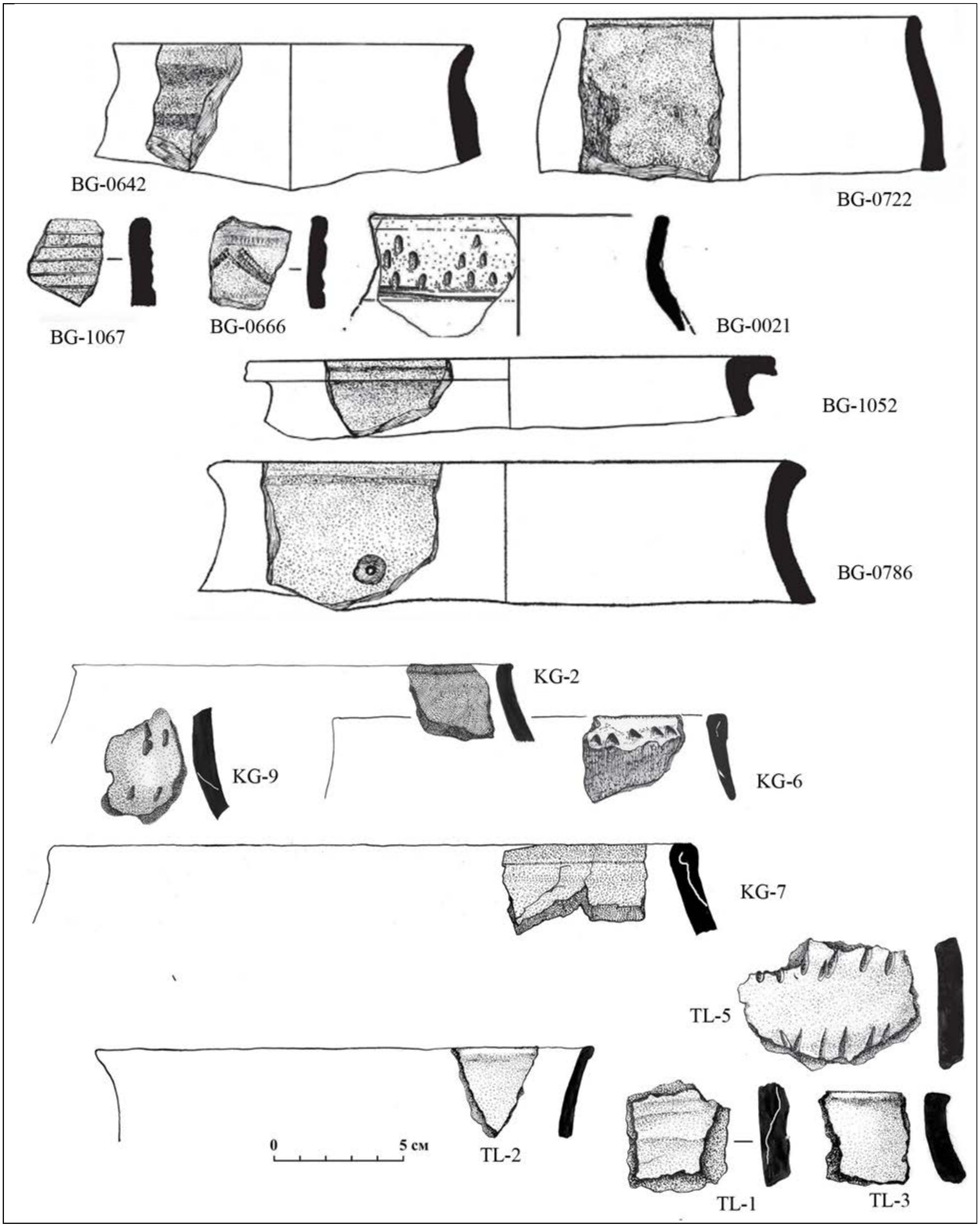

S1 Fig 7. Pottery illustrations of select samples from the Koksu Valley sites of Begash, Talapty and Kuigan in southeastern Kazakhstan. Labels refer to sample IDs that are also listed in S1 Table.

S1 Fig 8. Pottery illustrations of select samples from the Chu-Ili Valley sites of Tamgaly and Kuljabacy in southeastern Kazakhstan. Labels refer to sample IDs that are also listed in S1 Table.

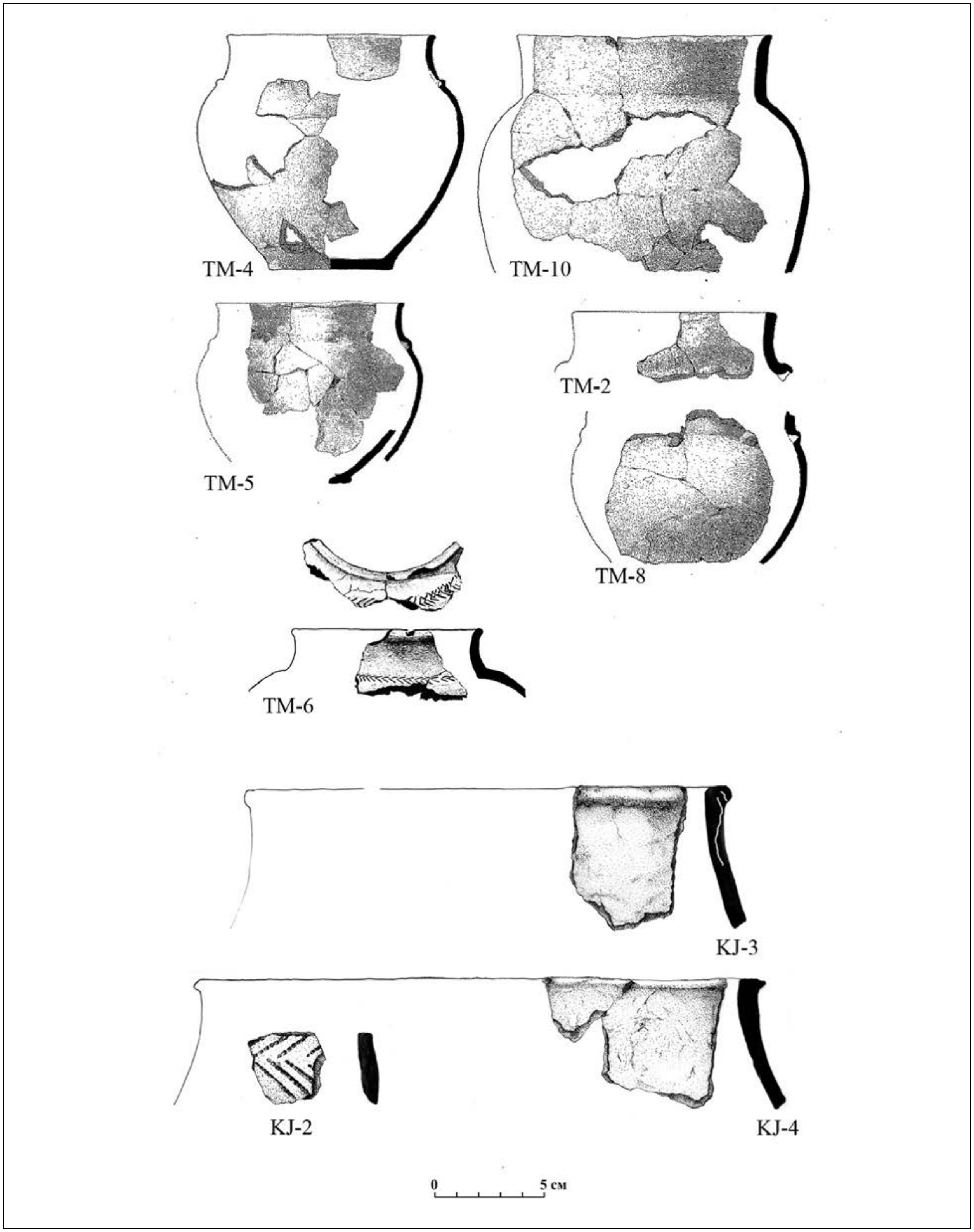

Supplement: S1 Fig — (PDF) [file pone.0320140.s002.pdf]
