# Supplementary material for: Rocks and clay: Potters’ technological choices within the cultural dynamics of Bronze Age Kazakhstan
Source: PLoS One. 2025 Apr 23;20(4):e0320140. doi: 10.1371/journal.pone.0320140 (PMC12017490; doi:10.1371/journal.pone.0320140)
Supplement: S1 File — (DOCX) [file pone.0320140.s003.docx]

Supplementary File: S1 File

Results of Petrographic Analysis of Zhetysu Bronze Age pottery, SE Kazakhstan

# Dali

## Petrographic Fabric A-DL: Granite tempered.

DL-4-01, DL-0036, DL-0092, DL-0767, DL-0395, DL-0499, DL-0774, DL-1224, DL-1018, DL-1031, DL-1066, DL-1015, DL-1041, DL-1063, DL-1458, DL-0271, DL-0449, DL-0593, DL-0595, DL-0789/0790, DL-0926, DL-1154, DL-1252, DL-1026, DL-0973, DL-1009, DL-4-05, DL-0542

**Comments**

This fabric represents ceramic sherds that were tempered with coarse fragments of acidic rocks (granite). The variability in these subfabrics is represented mildly by composition and clay preparation.

### Subfabric A1-DL: Weakly bimodal-to-polymodal.

DL-4-01, DL-0036, DL-0092, DL-0767, DL-0395, DL-0499, DL-0774, DL-1224, DL-1018, DL-1031, DL-1066, DL-1015, DL-1041, DL-1063, DL-1458

**Inclusions**

30%; <2.80 mm; eq.-el.; a.-r.; close to double spaced; weakly bimodal; poorly sorted; randomly oriented.

**Coarse Fraction**

60% (0.24-2.80 mm)

*Dominant*: feldspars often weathered; el.-eq. a. <2.80 mm, mode=0.64 mm. K-feldspar, plagioclase, perthite, myrmekite.

*Common:* quartz; el.-eq. a. <1.28 mm, mode=0.40 mm.

*Common:* acidic igneous plutonic coarse-grained rock fragments; eq. a.-sa. <2.80 mm, mode=0.64 mm. Composed of quartz and feldspars and a minor quantity of biotite (granite).

*Few:* biotite; el. a. <0.72 mm, mode=0.56 mm.

*Few:* amphibole; el. a. <0.56 mm, mode=0.48 mm.

*Few:* sericite; eq. a. <0.48 mm, mode=0.48 mm.

*Very rare-absent:* muscovite; el.-eq. a. <0.56 mm, mode=0.48 mm.

*Very rare-absent:* micrite; eq. r. <0.64 mm, mode=0.32 mm.

*Very rare-absent:* epidote; eq. r. <0.24 mm, mode=0.24 mm.

**Fine Fraction**

40% (0.02-0.24 mm)

*Dominant:* feldspars

*Dominant: quartz*

**Matrix**

50% Non-calcareous matrix ranging from brown to dark brown; moderate optical activity

**Voids**

20% Vughs, with some channels and planar voids (meso to mega); randomly oriented

**Comments**

The overall weakly bimodal to polymodal grain size distribution suggests that the clay was either uncleaned or only lightly cleaned before tempering.

| 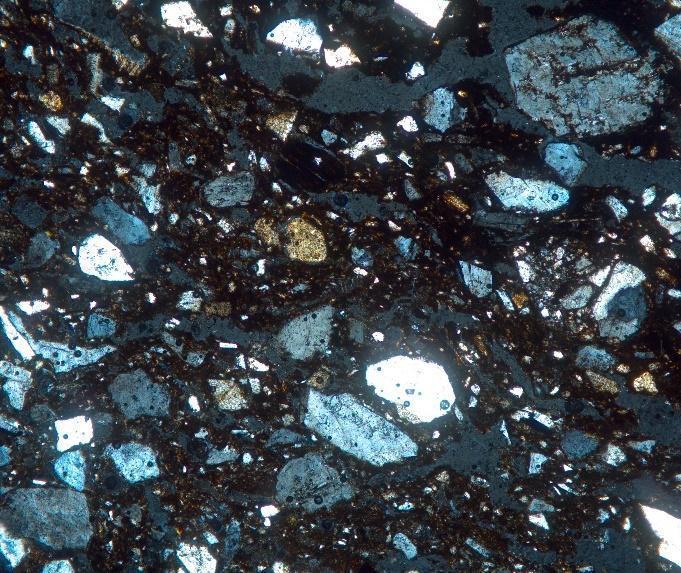 | 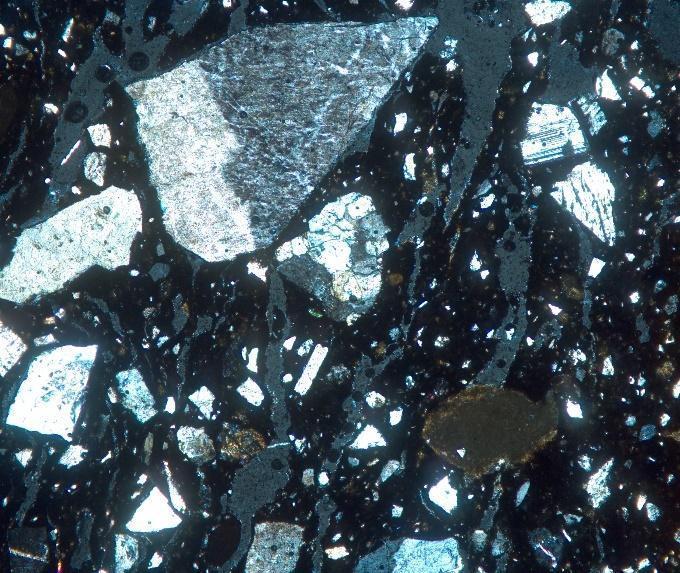 |
| --- | --- |
| DL-1458, 3 mm field of view | DL-774, 3 mm field of view |

### Subfabric A2-DL: Strongly bimodal with less abundant fine fraction.

DL-0271, DL-0449, DL-0593, DL-0595, DL-0789/0790, DL-0926, DL-1154, DL-1252, DL-1026, DL-0973, DL-1009

**Inclusions**

40%; <4 mm; eq.-el.; a.-sr.; single to double spaced; strongly bimodal; moderately sorted; randomly oriented.

**Coarse Fraction**

60% (0.24-4.00 mm)

*Dominant*: feldspars often weathered; eq.-el. a. <2.32 mm, mode=0.80 mm. K-feldspar, plagioclase, perthite, myrmekite.

*Common:* quartz; eq.-el. a. <3.20 mm, mode=0.56 mm.

*Common:* acidic igneous plutonic coarse-grained rock fragments; eq. a.-sa. <2.32 mm, mode=0.80 mm. Composed of quartz and feldspars (granite).

*Few:* biotite; el. a. <1.12 mm, mode=0.40 mm.

*Few:* amphibole; el. a. <0.96 mm, mode=0.48 mm.

*Few*: sericite; eq. sr. <0.72 mm, mode=0.72 mm.

*Rare:* epidote; eq. sr. <0.24 mm, mode=0.24 mm.

*Very rare:* mudstone fragment; el. sr. <0.96 mm, mode=0.96 mm.

*Very rare-absent:* acidic igneous medium-grained rock fragments; eq. sr.-a. <4.00 mm, mode=4.00 mm. Composed of quartz and feldspars.

**Fine Fraction**

40% (0.02-0.24 mm)

*Dominant:* quartz

**Matrix**

40% Non-calcareous matrix ranging from brown to dark brown; low optical activity.

**Voids**

20% Vughs (meso to mega), often connected by channels (meso to mega); sometimes parallel to the walls.

**Comments**

The low abundance and coarseness of the fine fraction could suggest that the clay was moderately cleaned prior to the addition of coarse temper.

| 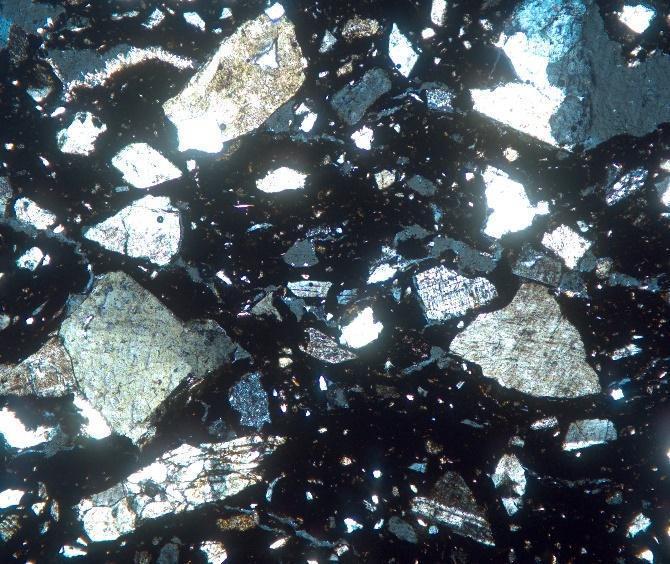 | 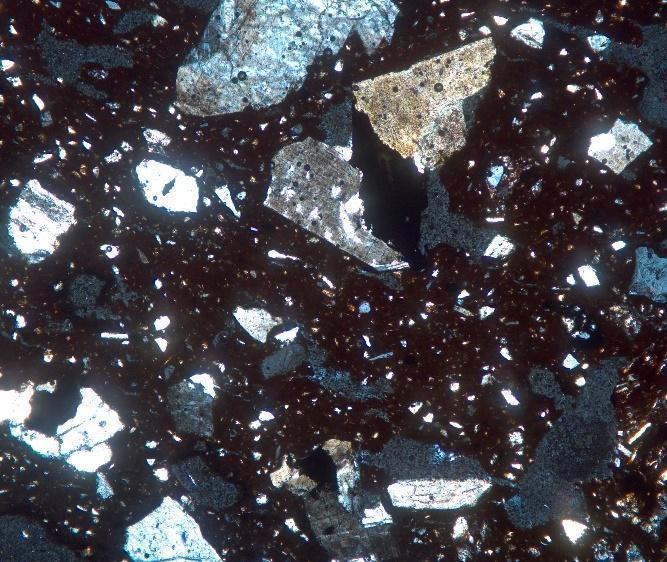 |
| --- | --- |
| DL-1026, 3 mm field of view | DL-0449, 3 mm field of view |

### Subfabric A3-DL: Strongly bimodal with well sorted fine fraction.

DL-4-05, DL-0542

**Inclusions**

40%; <2.80 mm; eq.-el.; a.-sr.; single spaced; strongly bimodal; moderately sorted; randomly oriented.

**Coarse Fraction**

65% (0.24-2.80 mm)

*Dominant*: feldspars often weathered; eq.-el. a. <2.32 mm, mode=0.56 mm. K-feldspar, plagioclase, perthite and myrmekite.

*Common:* quartz; eq.-el. sa. <2.80 mm, mode=0.72 mm.

*Common:* acidic igneous plutonic coarse-grained rock fragments; eq. a.-sa. <2.32 mm, mode=0.56 mm. Composed of quartz and feldspars (granite).

*Few:* biotite; el. a. <1.12 mm, mode=0.40 mm.

*Few:* amphibole; el. a. <0.96 mm, mode=0.40 mm.

*Few*: sericite; eq. sr. <0.64 mm, mode=0.40 mm.

*Rare:* epidote; eq. sr. <0.24 mm, mode=0.24 mm.

*Very rare:* grog fragments (containing well sorted feldspars and quartz); eq. r. <0.48 mm, mode=0.48 mm. The grog particles have neutral optical density, sharp boundaries, and discordance with the rest of the subfabric.

**Fine Fraction**

35% (0.01-0.16 mm)

*Dominant:* quartz

**Matrix**

40% Non-calcareous black matrix; low optical activity

**Voids**

20% Vughs with some channels (meso to mega); randomly oriented.

**Comments**

The strong bimodal grain-size distribution that marks this subfabric could suggest that the clay was well cleaned before tempering.

| 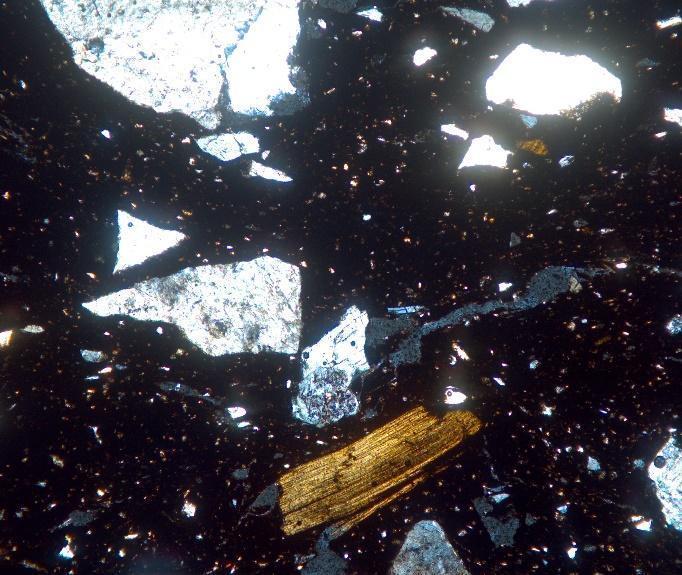 | 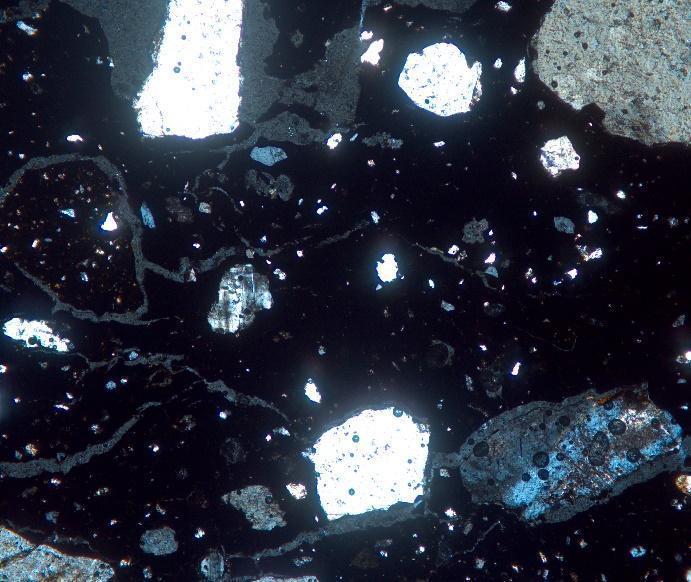 |
| --- | --- |
| DL-0542, 3 mm field of view | DL-4-05, 3 mm field of view |

## Petrographic Fabric B: Granite tempered with abundant calcite

DL-1017, DL-1446

**Comments**

This fabric represents ceramic that was tempered with coarse fragments of acidic igneous plutonic rocks (granite), but there is also abundant calcite.

### Subfabric B1-DL: Carbonate rock fragments

DL-1017

**Inclusions**

40%; <2.56 mm; eq.-el.; a.-r.; close to double spaced; weakly bimodal; poorly sorted; randomly oriented.

**Coarse Fraction**

60% (0.24-2.56 mm)

*Dominant*: feldspars often weathered; eq.-el.-eq. a. <1.68 mm, mode=0.88 mm. K-feldspar, plagioclase and perthite.

*Common:* quartz; el.-eq. sa. <1.12 mm, mode=0.40 mm.

*Common:* acidic igneous plutonic coarse-grained rock fragments; eq. a.-sa. <1.68 mm, mode=0.88 mm. Composed of quartz and feldspars (granite).

*Common:* carbonate sedimentary rock fragments; eq. sr.-sa. <2.56 mm, mode=1.28 mm. Composed of quartz, feldspars and calcite

*Few:* micrite; eq. r. <0.48 mm, mode=0.24 mm.

**Fine Fraction**

40% (0.02-0.16 mm)

*Dominant:* quartz

*Common:* feldspars

*Few:* micas

**Matrix**

40% Non-calcareous dark brown matrix; moderate optical activity.

**Voids**

20% Vughs with some channels and elongate planar voids (meso to macro); randomly oriented.

**Comments**

This subfabric contains common fragments of carbonatic sedimentary rocks.

| **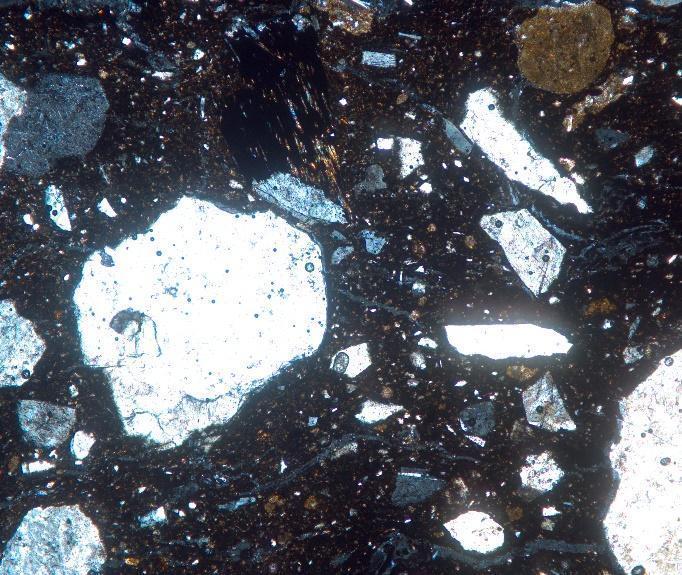** |  |
| --- | --- |
| DL-1017, 3 mm field of view |  |

### Subfabric B2-DL: Abundant micrite

DL-1446

**Inclusions**

30%; <2.64mm; eq.-el.; a.-sr.; close to open spaced; weakly bimodal; poorly sorted; randomly oriented.

**Coarse Fraction**

60% (0.24-2.64 mm)

*Frequent*: feldspars often weathered; eq.-el. a. <2.16 mm, mode=1.52 mm. K-feldspar, plagioclase and perthite.

*Frequent:* quartz; eq.-el. sa. <1.36 mm, mode=0.72 mm.

*Common:* acidic igneous plutonic coarse-grained rock fragments; eq. a.-sa. <2.16 mm, mode=1.52 mm. Composed of quartz and feldspars (granite).

*Common:* micrite; eq. sr.-sa. <2.64 mm, mode=0.56 mm.

*Few:* sparitic calcite; eq. a. <0.64 mm, mode=0.56 mm.

**Fine Fraction**

40% (0.02-0.16 mm)

*Dominant:* quartz

*Common:* feldspars

**Matrix**

40% Non-calcareous light brown matrix; moderate optical activity.

**Voids**

30% Vughs connected by channels (meso to mega); randomly oriented.

**Comments**

This subfabric contains large fragments of micrite.

| 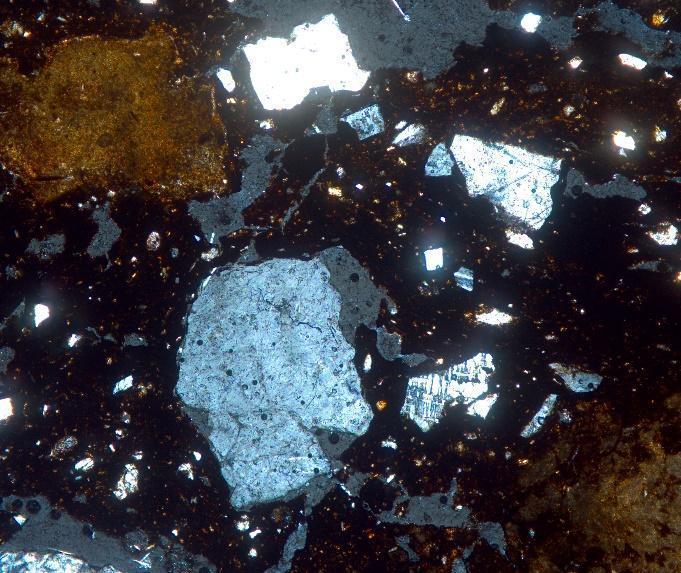 |  |
| --- | --- |
| DL-1446, 3 mm field of view |  |

## Petrographic Fabric C-DL: Polymodal with granite

DL-4-23, DL-0429, DL-1194, DL-1060

**Inclusions**

30% (0.24-1.28 mm); eq.-el.; a.-sr.; single to double spaced; polymodal; poorly sorted; randomly oriented.

*Dominant*: feldspars often weathered; eq.-el. a. <1.28 mm, mode=0.48 mm. K-feldspar, plagioclase and perthite.

*Common:* quartz; eq.-el. sa. <0.80 mm, mode=0.72 mm.

*Common:* acidic igneous plutonic coarse-grained rock fragments; eq. a.-sa. <1.28 mm, mode=0.48 mm. Composed of quartz and feldspars (granite).

*Few:* chert; eq. sr.-sa. <0.88 mm, mode=0.32 mm.

*Few:* amphibole; eq. a. <0.56 mm, mode=0.48 mm.

*Few:* micrite; el. sr. <0.72 mm, mode=0.56 mm.

**Matrix**

50% Non-calcareous light brown matrix; moderate optical activity.

**Voids**

20% predominantly vughs (micro to macro); randomly oriented.

**Comments**

This fabric is characterized by a polymodal grain size distribution and it is marked by acidic igneous plutonic rock fragments and derived minerals. The polymodal grain size distribution could indicate that the inclusions may be naturally occurring.

| 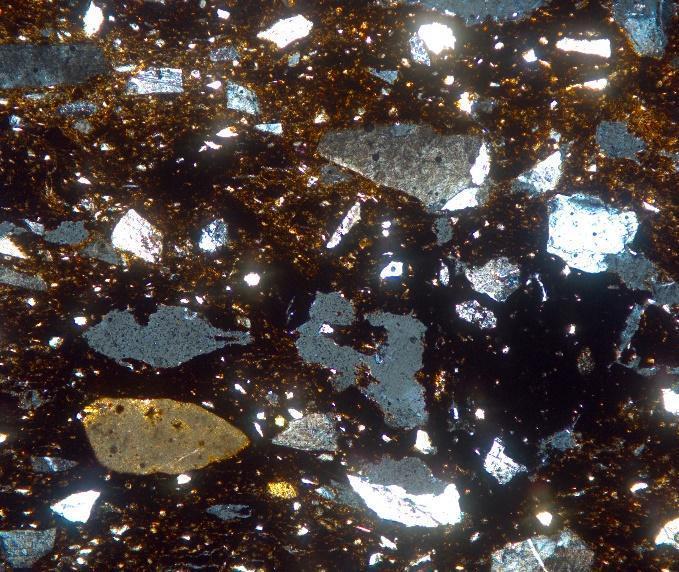 | 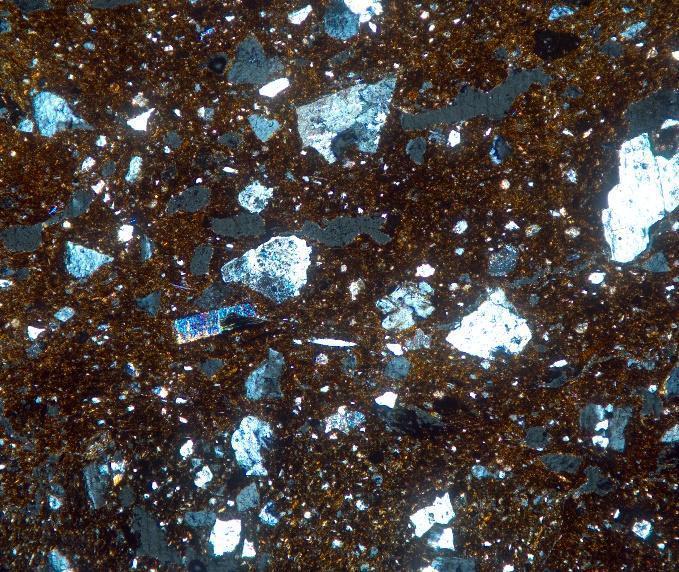 |
| --- | --- |
| DL 4-23, 3 mm field of view | DL 0429, 3 mm field of view |

## Petrographic Fabric D-DL: Tempered with granite and sedimentary rock fragments

DL-0797, DL-0891, DL-0280, DL-1238

**Inclusions**

30%; <3.52 mm; eq.-el.; a.-sr.; close to double spaced; weakly bimodal; poorly sorted; randomly oriented.

**Coarse Fraction**

60% (0.24-3.52 mm)

*Dominant*: feldspars often weathered; eq.-el. a. <3.52 mm, mode=0.88 mm. K-feldspar, plagioclase and perthite.

*Common:* quartz; eq.-el. sa. <1.20 mm, mode=0.24 mm.

*Common:* acidic igneous plutonic coarse-grained rock fragments; eq. a.-sa. <3.52 mm, mode=0.88 mm. Composed of quartz, feldspars, biotite and amphibole (granite).

*Common:* clastic sedimentary rock fragments; sandstones (arkosic arenite, wackes); eq. sr.-sa. <2.88 mm, mode=1.12 mm. Composed of quartz, feldspars and rarely muscovite.

*Few:* sparitic calcite; eq. a. <0.64 mm, mode=0.56 mm.

*Few:* chert; eq.-el. sa. <1.36 mm, mode=0.56 mm.

*Few:* micrite; eq. sr. <0.64 mm, mode=0.32 mm.

*Few:* biotite; eq.-el.-eq. a. <0.48 mm, mode=0.24 mm.

Very Few *:* amphibole; eq.-el. a. <0.56 mm, mode=0.32 mm.

*Very rare:* epidote; eq. sa. <0.24 mm, mode=0.24 mm.

**Fine Fraction**

40% (0.02-0.16 mm)

*Dominant:* quartz

**Matrix**

50% Non-calcareous light brown-to-very-dark-brown matrix; moderate optical activity.

**Voids**

20% Vughs connected by channels (meso to mega); randomly oriented.

**Comments**

This fabric contains large fragments of clastic sedimentary rocks of diverse nature, often composed of quartz. The bimodal distribution suggests that both granite and sedimentary rocks could have been added as temper.

| 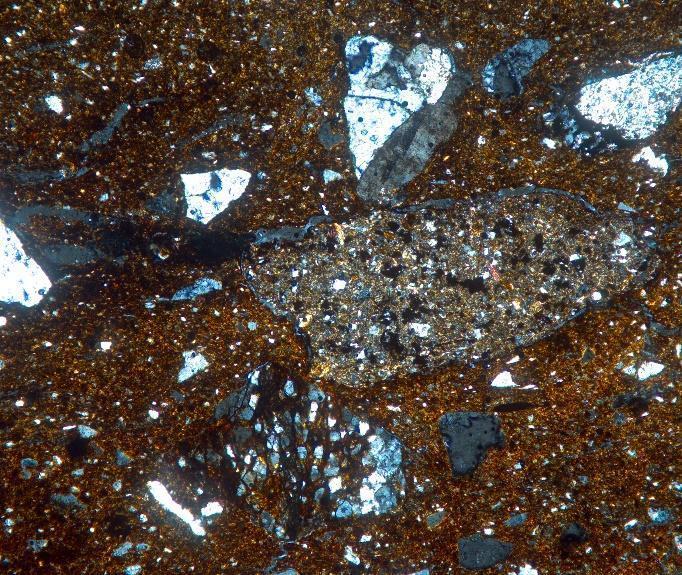 | 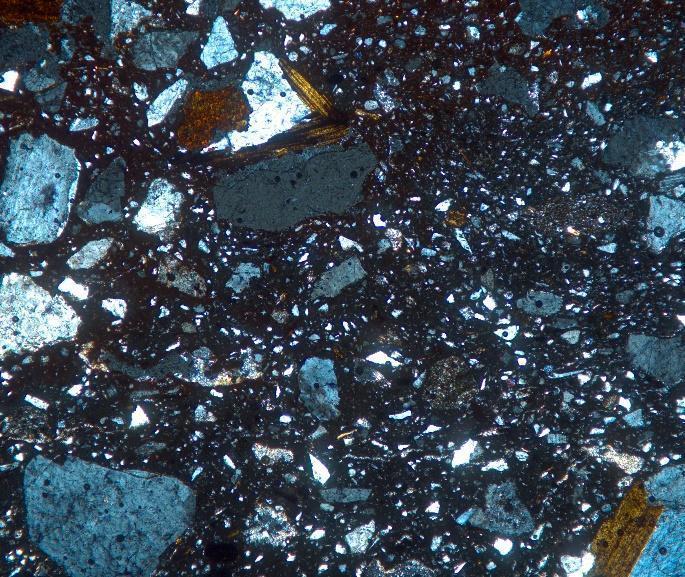 |
| --- | --- |
| DL-1238, 3 mm field of view | DL-0797, 3 mm field of view |

## Loners:

### Petrographic Fabric E-DL: Tempered with granite and metamorphic rock fragments.

DL-0895

**Inclusions**

20-30%; <4.08mm; eq.-el.; a.-r.; close to single spaced; weakly bimodal; poorly sorted; randomly oriented.

**Coarse Fraction**

50% (0.24-4.08 mm)

*Dominant*: feldspars often weathered; eq.-el. a. <4.08 mm, mode=1.52 mm. K-feldspar, plagioclase and perthite.

*Common:* quartz; eq.-el. sa. <0.80 mm, mode=0.32 mm.

*Common:* foliated metamorphic rock fragments; el. a. <2.72 mm, mode=0.56 mm. Composed of quartz, feldspars and muscovite.

Few: acidic igneous plutonic coarse-grained rock fragments; eq. a.-sa. <3.5 mm, mode=1.20 mm. Composed of quartz and feldspars (granite).

*Very rare:* grog fragment (containing poorly sorted quartz, feldspars and muscovite); eq. r. <0.96 mm, mode=0.40 mm. Low optical density, clear boundaries, and discordance within the fabric.

**Fine Fraction**

50% (0.02-0.24 mm)

*Dominant:* quartz

**Matrix**

50% Non-calcareous black matrix; low optical activity.

**Voids**

20-30% Vughs and some channels (meso to mega); randomly oriented.

**Comments**

This bimodal fabric represents a not well-cleaned clay with temper consisting of some fragments of acidic igneous plutonic and metamorphic rock fragments.

| 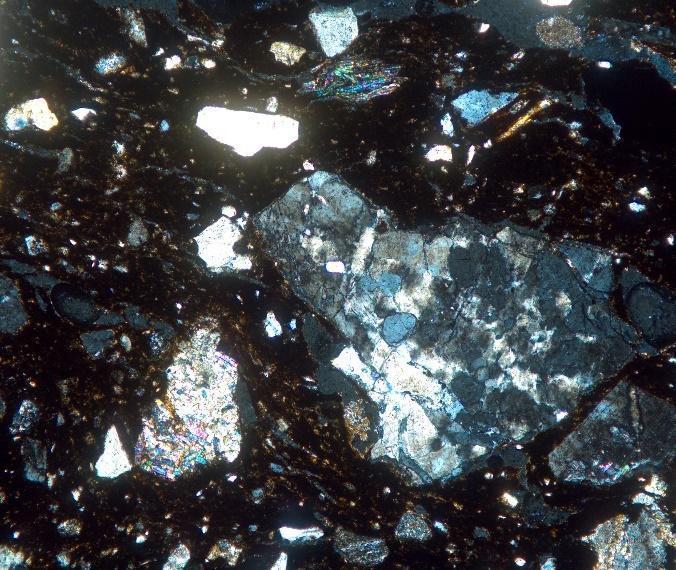 |  |
| --- | --- |
| DL-0895, 3 mm field of view |  |

# Tasbas

## Petrographic Fabric A-TB: Granite tempered

TB-126B16-n/a7, TB-0134, TB-0151, TB-0160, TB-102C16-n/a1, TB-0040, TB-0021, TB-0081, TB-0132, TB-0143, TB-0145, TB-0136, TB-0152/0153, TB-0156, TB-102C16-n/a2, TB-0113, TB-0061, TB-0076, TB-0110, TB-0242, TB-0129

### Subfabric A1-TB: Strongly bimodal with abundant fine fraction

TB-126B16-n/a7, TB-0113, TB-0134, TB-0151, TB-0160, TB-102C16-n/a1, TB-0040, TB-0021

**Inclusions**

20-30%; <4.56 mm; eq.-el.; a.-r.; single to open spaced; strongly bimodal; moderately sorted; randomly oriented.

**Coarse Fraction**

30% (0.32-4.56 mm)

*Dominant*: feldspars often weathered;eq.-el. a.<4.56 mm, mode=1.20 mm. K-feldspar, plagioclases, myrmekite, perthite.

*Common*: quartz; eq.-el. a.-sa. <1.44 mm, mode=0.48 mm.

*Common:* acidic igneous plutonic coarse-grained rock fragments; eq. a.-sa. <4.56 mm, mode=1.20 mm. Composed of quartz and feldspars (granite).

*Common:* biotite; eq.-el. a.-sa. <1.12 mm, mode=0.64 mm.

*Few:* sparitic calcite; eq.-el. a.-r. <1.36 mm, mode=0.56 mm. Most notable in TB 021

*Very rare-absent:* epidote; eq. sr. <0.32 mm, mode=0.32 mm.

*Very rare-absent:* clastic sedimentary rock fragments (sandstone); eq. r. <1.04 mm, mode=1.0 mm. Composed of quartz (possible quartz arenite).

*Very rare-absent:* mudstone fragments; el. sa. <1.12 mm, mode=1.12 mm.

*Very rare-absent:* foliated metamorphic rock fragments; eq. r. <1.04 mm, mode=1.0 mm. Composed of muscovite and quartz.

**Fine Fraction**

70% (0.02-0.24 mm)

*Dominant*: quartz

*Common*: feldspars

*Few:* micas

*Few:* epidote

**Matrix**

50% Non-calcareous matrix ranging from very dark brown to brown; moderate optical activity.

**Voids**

20% Predominantly elongated vughs (meso to mega); randomly oriented.

**Comments**

The strong bimodality of this subfabric indicates tempering with fragments of acidic igneous plutonic rocks. The fine fraction is abundant, but well sorted.

| 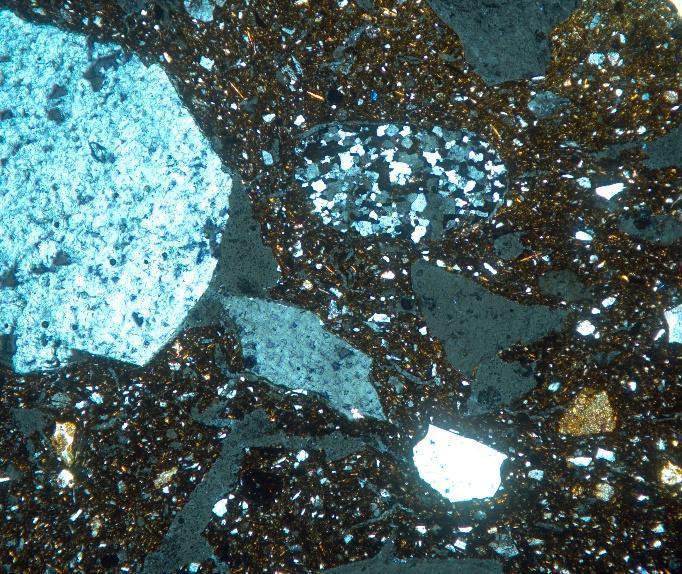 | 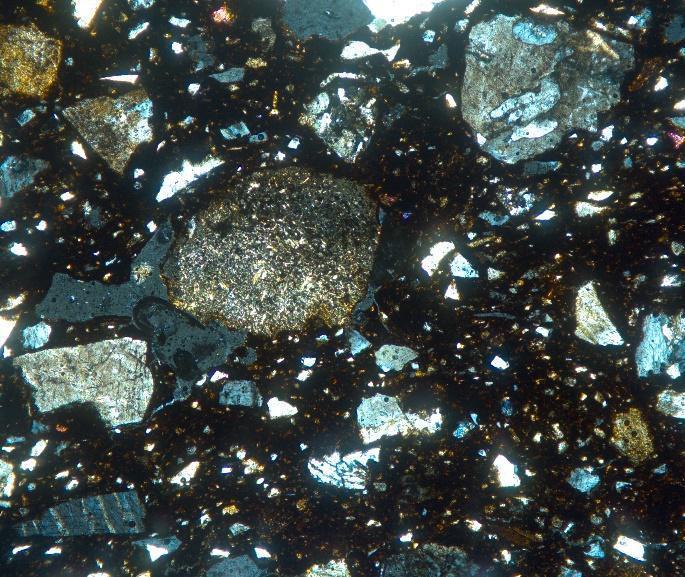 |
| --- | --- |
| TB-0021, 3 mm field of view | TB-0040, 3 mm field of view |

### Subfabric A2-TB: Strongly bimodal with moderately abundant fine fraction

TB-0081, TB-0132, TB-0143, TB-0145

Inclusion

30%; <2.40mn; eq.-el.; a.-r.; single to open spaced; strongly bimodal; poorly sorted; randomly oriented.

**Coarse Fraction**

40% (0.16-2.40 mm)

*Dominant*: feldspars, often weathered; eq.-el. a. <2.08 mm, mode=1.12 mm. K-feldspar, plagioclase and perthite.

*Common:* quartz; eq. a. <1.52 mm, mode=0.56 mm. *Common:* biotite; el. a. <0.88 mm, mode=0.32 mm.

*Common:* acidic igneous plutonic coarse-grained rock fragments; eq. a.-sa. <1.84 mm, mode=1.36 mm. Composed of quartz and feldspars and sometimes biotite (granite).

*Few:* micrite; eq. sr. <2.40 mm, mode=0.64 mm.

Few: amphibole; el. a. <0.64 mm, mode=0.40 mm

*Rare:* epidote; eq. r. <0.16 mm, mode=0.16 mm.

*Very rare-absent:* grog (containing well-sorted quartz, feldspars and muscovite); eq. sr. <1.84 mm, mode=1.84 mm. Represented by only a single fragment in TB 143.High optical density, clear to diffuse boundaries and discordant with the rest of the fabric.

*Very rare-absent:* possible mudstone fragments; eq.-el. a. <1.28 mm, mode=1.28 mm.

**Fine Fraction**

60% (0.02-0.16 mm)

*Dominant*: quartz

*Common*: feldspars

*Few*: muscovite

**Matrix**

50% Non-calcareous matrix ranging from very dark brown to brown; moderate optical activity.

**Voids**

20% Predominantly elongated vughs (meso to mega); randomly oriented.

**Comments**

The strong bimodality of this subfabric indicates a tempering with fragments of acidic igneous plutonic rocks. The fine fraction is moderately abundant TB 143 contains a singular ceramic fragment, which may have been included by accident.

| 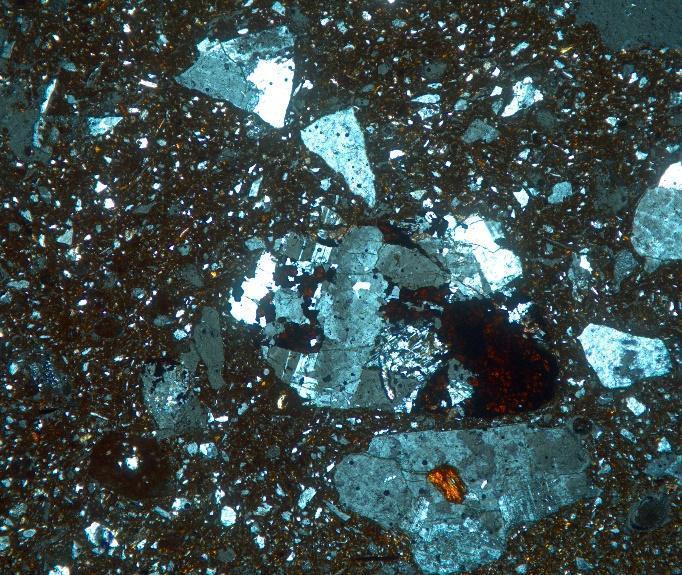 | 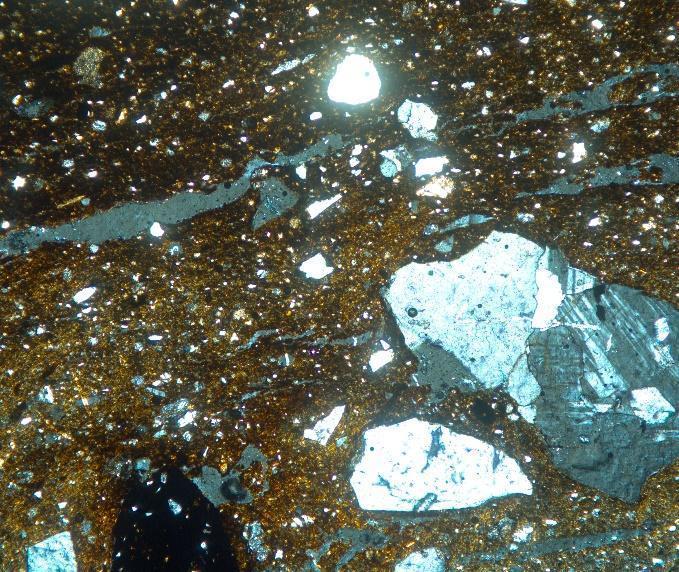 |
| --- | --- |
| TB-0132, 3 mm field of view | TB-0143, 3 mm field of view |

### Subfabric A3-TB: Bimodal with scarce fine fraction

TB-0136, TB-0152/0153, TB-0156, TB-102C16-n/a2

**Inclusions**

40%; <3.36 mm; eq.-el.; a.-r.; single to open spaced; weakly bimodal; poorly sorted; randomly oriented.

**Coarse Fraction**

50% (0.24-3.36 mm)

*Dominant:* feldspars, very weathered; eq.-el. a. <3.36 mm, mode=1.36 mm. K-feldspar, plagioclase and perthite.

*Common:* quartz; eq.-el. a. <1.76 mm, mode=0.88 mm. Very weathered and angular grains.

*Common:* acidic igneous plutonic coarse-grained rock fragments; eq. a. <3.28 mm, mode=1.36 mm. Composed of quartz and feldspars and sometimes biotite and amphiboles (granite).

Few: biotite; el. a. <1.28 mm, mode=0.72 mm.

*Few:* amphibole; eq.-el. a. <0.88 mm, mode=0.40 mm. *Few:* micrite; eq.-el. r. <1.52 mm, mode=0.56 mm.

*Very rare-absent:* sparitic calcite; eq.-el. sr. <2.00 mm, mode=2.00 mm.

*Very rare-absent: clastic* sedimentary rock fragments; claystone; el. sr. <1 mm. Only found in TB 156.

*Very rare-absent:* foliated metamorphic rock fragments; el. sr. <1 mm. Composed of quartz.

**Fine Fraction**

50% (0.02-0.16 mm)

*Dominant*: quartz

*Common*: feldspars

**Matrix**

40% Non-calcareous matrix ranging from light brown to black; low optical activity.

**Voids**

20% Predominantly elongated vughs (meso to mega) and channels (meso to macro); randomly oriented.

**Comments**

Samples in this subfabric seem to be tempered with acidic igneous rocks that contain more biotite than amphibole.

| 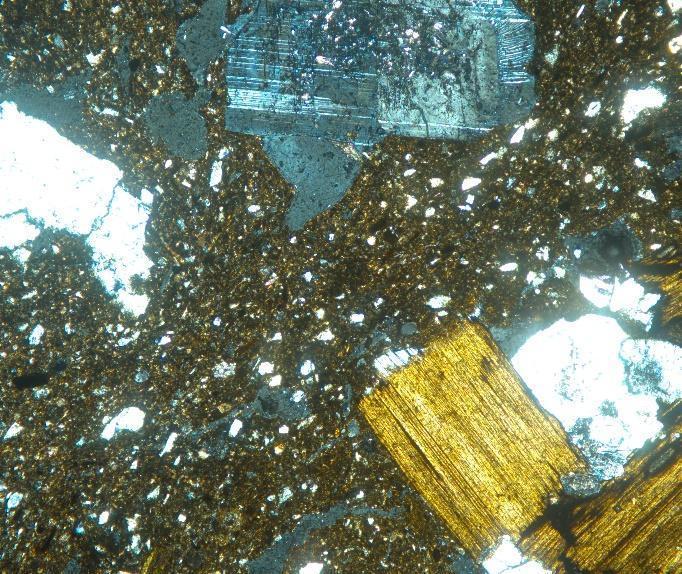 | 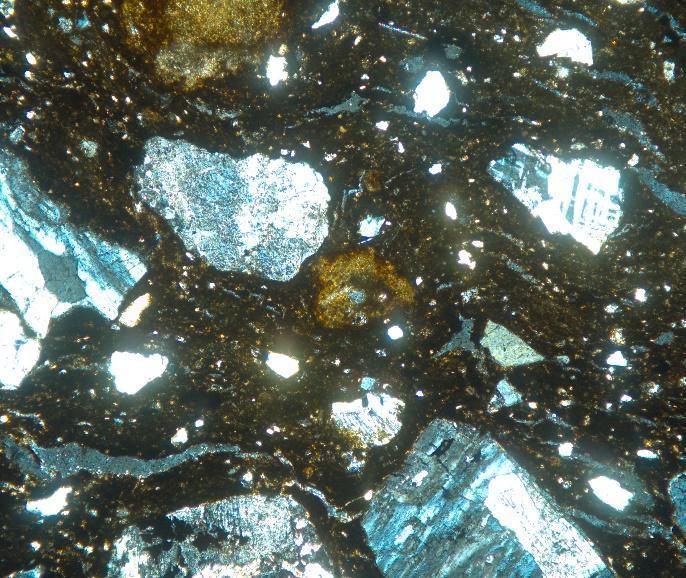 |
| --- | --- |
| TB-0136, 3 mm field of view | TB-102C16-n/a2, 3 mm field of view |

### Subfabric A4-TB: Bimodal with very scarce fine fraction

TB-0061, TB-0076, TB-0110, TB-0129

**Inclusions**

30%; <3.36 mm; eq.-el.; a.-sr.; close to open spaced; weakly bimodal; poorly sorted; randomly oriented.

**Coarse Fraction**

60% (0.24-3.36 mm)

*Dominant:* feldspars; eq.-el. a. <1.44 mm, mode=1.20 mm. K-feldspar, plagioclase, perthite.

*Common:* quartz; eq.-el. a. <2.72 mm, mode=0.72 mm.

*Common:* acidic igneous plutonic coarse-grained rock fragments; eq. a. <2.72 mm, mode=1.20 mm. Composed of quartz and feldspars (granite).

*Few:* sparitic calcite; eq. sr. <0.40 mm, mode=0.04 mm.

*Very rare-absent:* foliated metamorphic rock fragments; el. a. <1.52 mm. Composed of quartz.

*Very rare*: one fragment of grog (only in sample TB061, containing moderately sorted monocrystalline and polycrystalline quartz, feldspars and one fragment of unfoliated metamorphic rock); el. a. <2 mm. It has high optical density, clear to sharp boundaries, and discordance within the fabric.

**Fine Fraction**

40% (0.02-0.16 mm)

*Dominant*: quartz

*Common*: feldspars

**Matrix**

50% Non-calcareous matrix ranging from light brown to black, low optical activity.

**Voids**

20% Predominantly elongated vughs and channels (meso to mega), and organic voids (TB110 and TB129); randomly oriented.

**Comments**

Overall, the samples in this subfabric notably lack the presence of biotite and amphibole which characterize other samples. This may be due to the use of a more acidic igneous plutonic rock source as a temper of a very fine or well cleaned clay. TB-0076 contains abundant micrite. TB-0110 contains a finer coarse fraction than other samples in this subfabric, which may indicate the use of finer tempering material. TB-0061 contains a single fragment of ceramic.

| 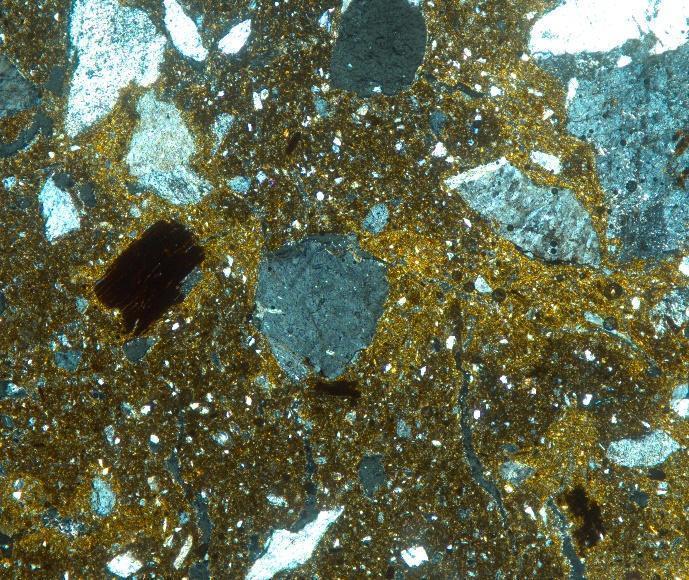 | 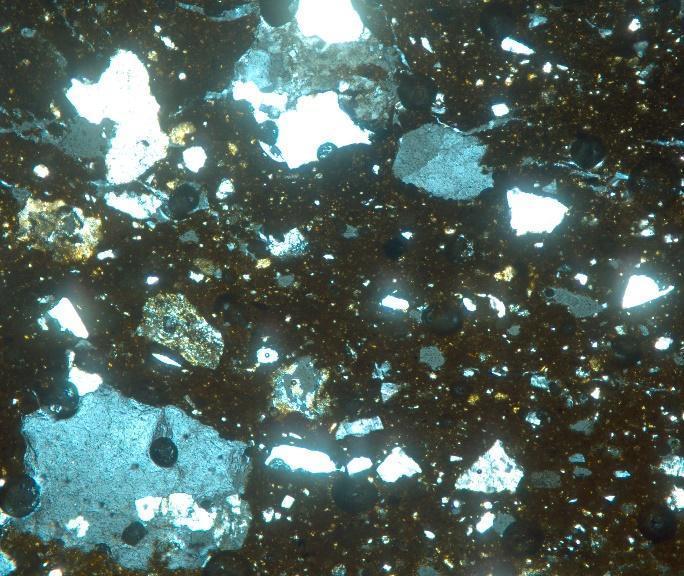 |
| --- | --- |
| TB 0061, 3 mm field of view | TB 0129, 3 mm field of view |

### Subfabric A5-TB: Abundant biotite

TB-0242

**Inclusions**

40%; <1.36 mm; eq.-el.; a.; close to double spaced; moderately bimodal; moderately sorted; randomly oriented.

**Coarse Fraction**

60% (0.32-1.36 mm)

*Dominant:* feldspars; eq.-el. a. <1.36 mm, mode=0.64 mm. K-feldspar, plagioclase, perthite.

*Common:* quartz; eq.-el. a. <0.96 mm, mode=0.64 mm.

*Common:* biotite; el. a. <0.72 mm, mode=0.48 mm.

*Common:* acidic igneous plutonic coarse-grained rock fragments; eq. a. <1.36 mm, mode=0.64 mm. Composed of quartz and feldspars (granite).

Few: amphibole; el. a. <0.88 mm, mode=0.48 mm.

*Few:* foliated metamorphic rock fragments; el. a. <1.52 mm, mode=0.64 mm. Composed of quartz and muscovite.

**Fine Fraction**

40% (0.02-0.16 mm)

*Dominant*: quartz

*Dominant*: feldspars

**Matrix**

40% Non-calcareous matrix, dark brown; moderate optical activity.

**Voids**

20% Predominantly vughs, both elongate and equant and channels (meso to macro); randomly oriented.

**Comments**

More significantly, unlike other samples from Tabash, the tempering of subfabric 5 is intentionally crushed and homogenized and then subsequently added to a fine or well-cleaned clay.

| 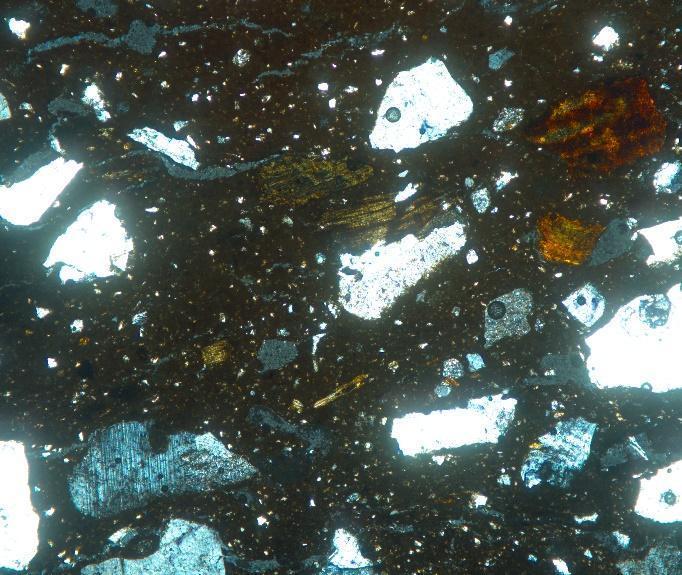 |  |
| --- | --- |
| TB-0242, 3 mm field of view |  |

## Petrographic Fabric B-TB: Grog tempered.

TB-17A13-n/a3, TB-0220, TB-09C16-n/a6, TB-0127, TB-0059, TB-0083; TB-0149

### Subfabric B1-TB: With light matrix tempered only with grog.

TB-17A13-n/a(3), TB-0220

**Inclusions**

40%; <1.36 mm; eq.-el.; a.-sr.; single to open spaced; strongly bimodal; well- sorted; randomly oriented.

**Coarse Fraction**

40% (0.32-1.36 mm)

*Dominant:* grog (containing well-sorted monocrystalline and polycrystalline quartz, feldspars, and few muscovite); eq.-el. sa.-a. <1.36 mm, mode=0.88 mm. Often very dark brown to black, fired under reducing conditions. Neutral optical density, clear boundaries, and discordance with the rest of the fabric.

*Common:* feldspars, weathered; eq.-el. a. <1.20 mm, mode=0.88 mm. K-feldspar and plagioclase.

*Common:* quartz; eq. a. <1.12 mm, mode=0.32 mm.

*Rare-absent:* amphibole; el. a. <0.72 mm, mode=0.32 mm.

*Rare-absent:* sparitic calcite; eq. sr. <0.48 mm.

*Rare-absent:* foliated metamorphic rocks fragments; eq. sr. <0.5 mm. Composed of quartz and feldspars.

*Rare-absent:* acidic igneous plutonic coarse-grained rock fragments; eq. sr. <1.6 mm. Composed of quartz and feldspars.

**Fine Fraction**

60% (0.02-0.16 mm)

*Dominant:* quartz

*Common:* feldspars

*Common:* micas

**Matrix**

50% Non-calcareous matrix, very light brown and shows mild optical activity.

**Voids**

10% Predominantly vughs (meso to macro); randomly oriented.

**Comments**

Clay could have been mildly processed before the addition of well processed grog (crushed and sieved).

| 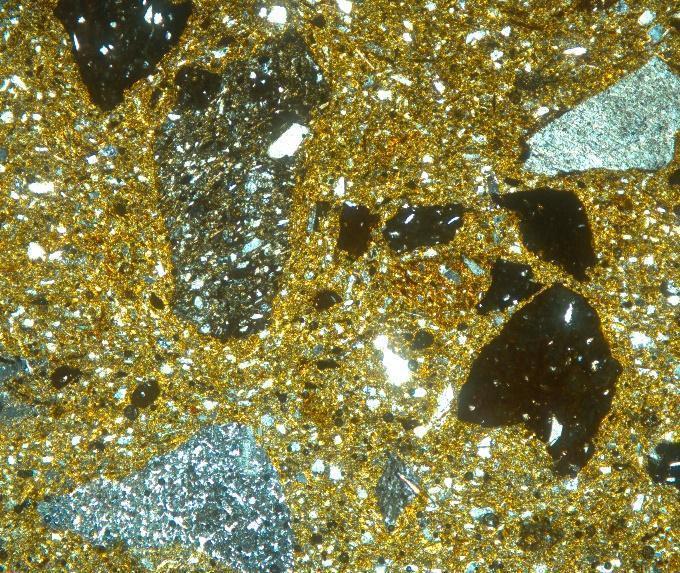 | 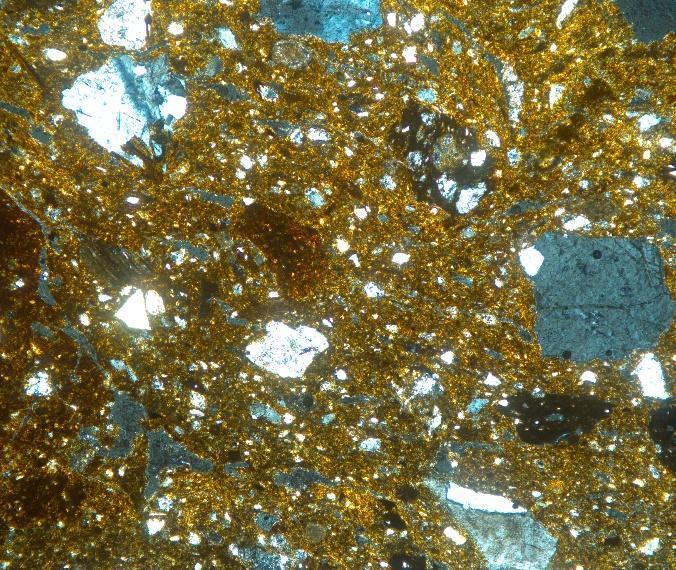 |
| --- | --- |
| TB-17A13-n/a3, 3 mm field of view | TB-0220, 3 mm field of view |

### Subfabric B2-TB: Granite tempered, with well sorted fine fraction.

TB-09C16-n/a6

**Inclusions**

40%; <2.48 mm; eq.-el.; a.-sr.; close to open spaced; strongly bimodal; moderately sorted; randomly oriented.

**Coarse Fraction**

40% (0.32-2.48 mm)

*Dominant:* grog (containing moderately sorted quartz, feldspars, and rarely biotite and pyroxenes; eq.-el. sa.-a. <2.48 mm, mode=1.36 mm.. Low optical density, clear to merging boundaries, discordance within the fabric.

*Common:* feldspars, often very weathered; eq.-el. a. <2.08 mm, mode=0.48 mm. Includes k-feldspar, plagioclase and perthite.

*Common:* quartz; eq. a. <1.92 mm, mode=0.56 mm.

*Common*: acidic igneous plutonic coarse-grained rock fragments; eq. sr. <2.8 mm, mode=0.48 mm. Composed of quartz and feldspars.

*Few:* biotite; el. a. <0.32 mm, mode=0.24 mm.

*Rare-absent:* igneous acidic medium-grained rock fragments; eq. a. <0.72 mm, mode=0.72 mm. Composed of quartz and feldspars.

**Fine Fraction**

60% (0.02-0.16 mm)

*Dominant:* quartz

*Common*: feldspars

**Matrix**

50% Non-calcareous matrix, very dark brown-to-black with low optical activity.

**Voids**

10% Predominantly vughs and channels (meso to mega); randomly oriented.

**Comments**

This subfabric exhibits moderately-to-well cleaned clay with the addition of coarse grog and coarse acidic igneous plutonic rock fragments (granite). Grog seems to be less well sorted in comparison to subfabric1. The matrix is a dark brown color, possibly indicating its firing under reducing conditions.

| 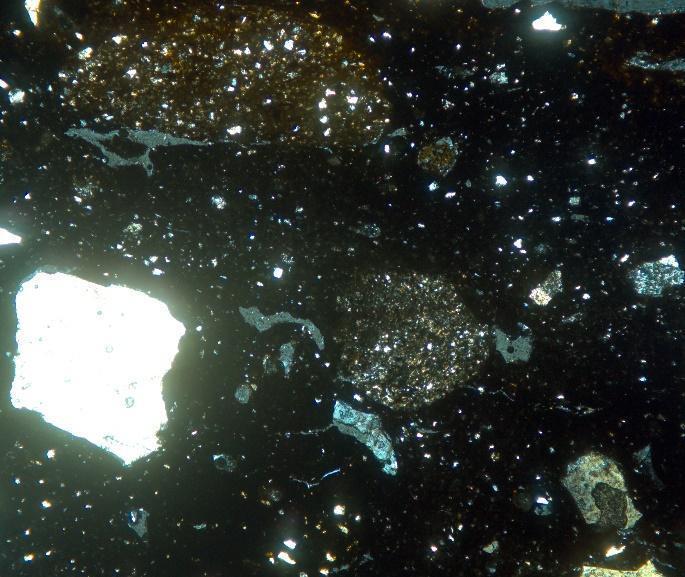 |  |
| --- | --- |
| TB-096C16-n/a6, 3 mm field of view |  |

### Subfabric B3-TB: Granite tempered with moderately sorted fine fraction.

(TB-059, TB-083, TB-127)

**Inclusions**

40%; <2.56 mm; eq.-el.; a.-r.; single to double spaced; strongly bimodal; well-sorted; randomly oriented.

**Coarse Fraction**

35% (0.32-2.56 mm)

*Frequent:* grog (containing moderately sorted quartz, feldspars, muscovite and biotite); q.-el. sa.-a. <2.40 mm, mode=1.20 mm. Grog particles have low to neutral optical density, clear boundaries and discordant with the rest of the fabric.

*Frequent:* feldspars, often very weathered; eq.-el. a. <2.56 mm, mode=0.88 mm. K-feldspar, plagioclase, perthite, mymekite.

*Common*: acidic igneous plutonic coarse-grained rock fragments; eq. sr. <2.56 mm, mode=0.88 mm. Composed of quartz and feldspars.

*Few:* quartz, very weathered; eq.-el. a. <0.96 mm, mode=0.56 mm.

*Few:* biotite; el. a. <0.48 mm, mode=0.32 mm.

*Very Few:* amphibole; el. a. <0.24 mm, mode=0.24 mm.

*Very few:* foliated metamorphic rock fragments; el. a. <0.88 mm, mode=0.32 mm. Composed of quartz and minor quantity of biotite and epidote.

*Very few:* micrite; eq. r. <1.92 mm, mode=0.72 mm.

**Fine Fraction**

65% (0.02-0.16 mm)

*Dominant*: quartz

*Common:* feldspars

*Few:* micas.

**Matrix**

40% Non-calcareous matrix, very dark brown-to-black with low optical activity.

**Voids**

20% Predominantly large and elongated vughs and channels (meso to mega); randomly oriented.

**Comments**

This subfabric could represents a moderately-to-uncleaned clay with a somewhat heterogeneous coarse fraction. The tempering agents seem to be acidic igneous plutonic rock fragments (granite) and grog.

| 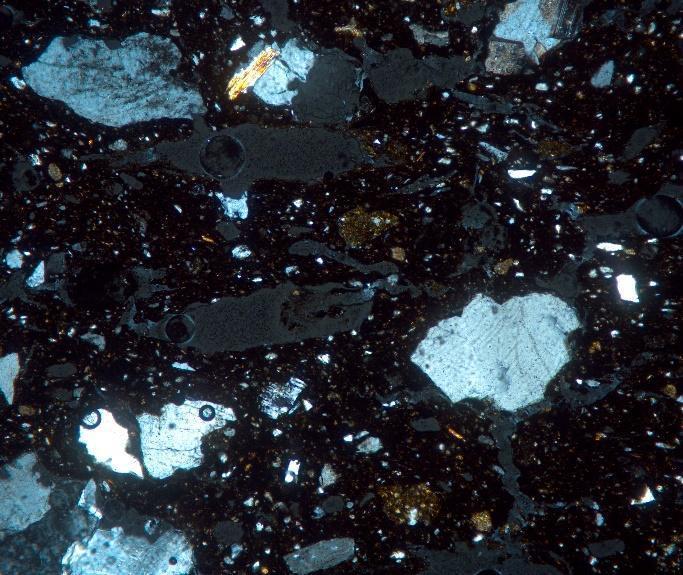 | 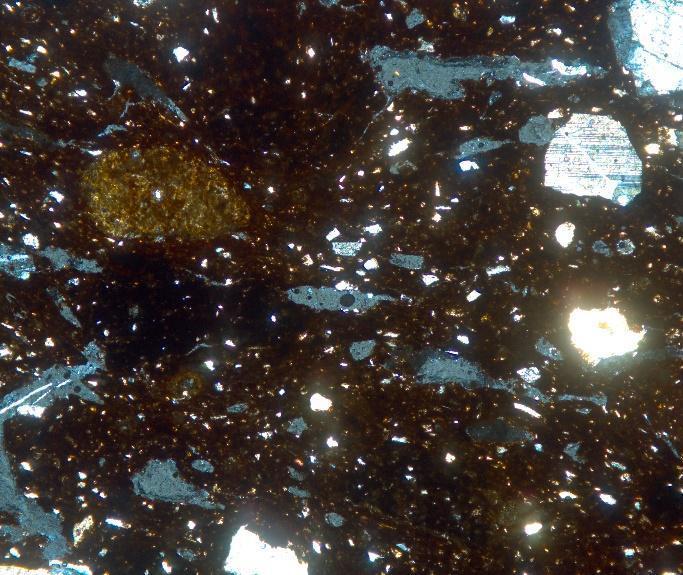 |
| --- | --- |
| TB-0083, 3 mm field of view | TB-0127, 3 mm field of view |

### Subfabric B4-TB With fragments of metamorphic rocks

TB-0149

**Inclusions**

40%; <3.68 mm; eq.-el.; a.-r.; single to double spaced; strongly bimodal; well-sorted; randomly oriented.

**Coarse Fraction**

20% (0.32-3.68 mm)

*Frequent:* grog (containing poorly sorted quartz, feldspars, muscovite); eq.-el. sa.-a. <3.04 mm, mode=1.60 mm. High optical density, diffuse to merging boundaries and discordance within the fabric.

*Frequent:* foliated metamorphic rock fragments; el. a. <3.60 mm, mode=1.12 mm. Composed of quartz.

*Few:* feldspars, often very weathered; eq.-el. a. <1.20 mm, mode=0.80 mm. K-feldspar and plagioclase.

*Few*: acidic igneous plutonic coarse-grained rock fragments; eq. sr. <1.20 mm, mode=0.80 mm. Composed of quartz and feldspars.

*Few:* clastic sedimentary rock fragments; el. a. <1.40 mm, mode=0.16 mm.

*Few:* quartz, very weathered; eq.-el. a. <1.52 mm, mode=0.64 mm.

*Very few:* micrite; eq. r. <0.72 mm, mode=0.72 mm.

**Fine Fraction**

80% (0.02-0.16 mm)

*Dominant*: quartz

*Common:* feldspars

**Matrix**

40% Non-calcareous matrix, very dark brown-to-black with low optical activity.

**Voids**

10% Predominantly vughs (meso to mega); randomly oriented.

**Comments**

This subfabric could represent not well cleaned clay that features metamorphic rock fragments, alongside sedimentary and acidic igneous plutonic rock fragments as well as grog. The tempering agent seems to be only grog, while the rock fragments could be naturally occurring.

| 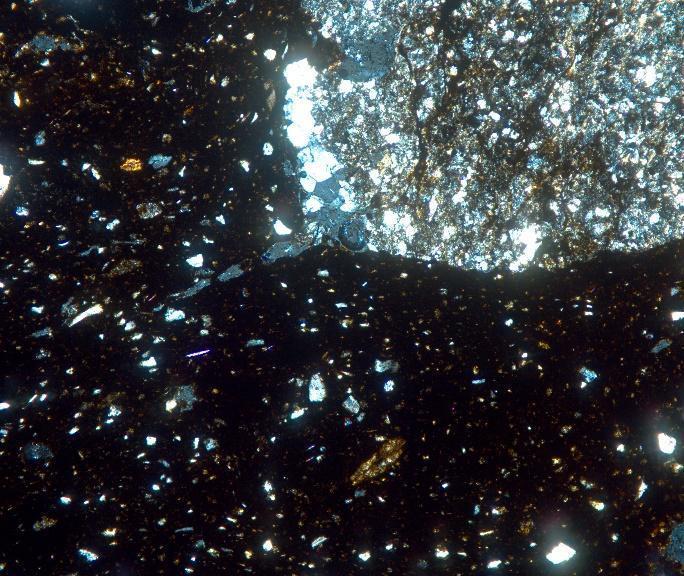 |  |
| --- | --- |
| TB-0149, 3 mm field of view |  |

## Petrographic Fabric C-TB: Polymodal fabric

TB-0038, TB-0122, TB-0214

**Inclusions**

50%; <3.20 mm; eq.-el.; a.-sr.; close to single spaced; polymodal; poorly sorted; randomly oriented.

*Frequent:* feldspars, very weathered. eq.-el. a. <3.20 mm, mode=0.56 mm. K-feldspar, plagioclase, perthite and sericite.

*Frequent:* quartz; eq.-el. a. <1.36 mm, mode=0.32 mm.

*Few:* micrite; eq. sr. <2.48 mm, mode=0.64 mm.

*Few:* acidic igneous plutonic fine-grained rock fragments; el. a. <2.08 mm, mode=2.08 mm. Composed of quartz and feldspars.

*Very few:* amphibole; eq. sr. <0.40 mm, mode=0.16 mm.

*Rare*: foliated metamorphic rock fragments; el. sr. <1.5 mm. Composed of quartz.

*Rare:* biotite; eq.-el. sa. <0.32 mm, mode=0.16 mm.

*Rare:* epidote; eq. sa. <0.24 mm, mode=0.16 mm.

*Very rare-absent:* grog (containing poorly sorted feldspars- K-feldspars and perthite-, quartz, muscovite and rare amphibole); eq.-el. sa. <2.16 mm, mode=2.16 mm. High optical density, clear to merging boundaries, and discordance.

**Matrix**

40% Non-calcareous matrix, very dark brown to black; moderate optical activity.

**Voids**

10% Predominantly vughs (meso to macro); randomly oriented.

**Comments**

The polymodal grain size distribution of inclusions deriving from acidic plutonic rocks (granite) leaves no clear evidence for tempering.

| 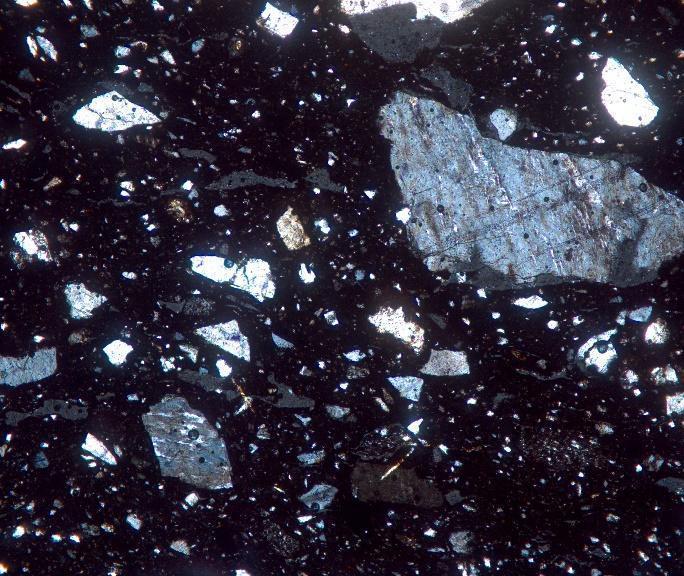 | 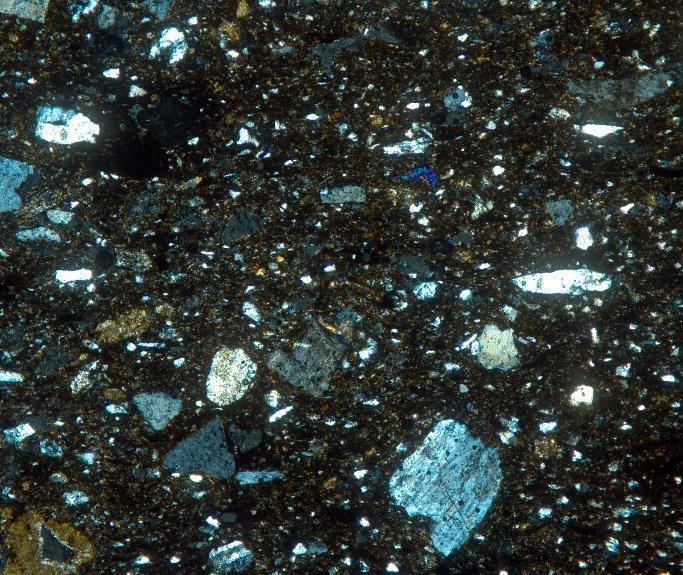 |
| --- | --- |
| TB-0038, 3 mm field of view | TB-0214, 3 mm field of view |

## Petrographic Fabric D-TB: Tempered with volcanic rock fragments

TB-0025, TB-0105

**Inclusions**

50%; <1.52 mm; eq.-el.; a.-r.; close to single spaced; weakly bimodal; poorly sorted; randomly oriented.

**Coarse Fraction**

60% (0.32-1.52 mm)

*Dominant:* basic fine-grained volcanic rock fragments; eq. sa.-a. <1.52 mm, mode=1.12 mm. Composed predominantly of fine feldspars.

*Frequent:* feldspars, often weathered; el. a. <1.44 mm, mode=0.56 mm. K-feldspar, plagioclase.*Few:* quartz, very weathered; eq.-el. a. <0.88 mm, mode=0.56 mm.

*Rare:* foliated metamorphic rock fragments; el. a. <0.64 mm, mode=0.64 mm. Composed of quartz.

*Very rare:* micrite; eq. r. <0.48 mm, mode=0.48 mm.

**Fine Fraction**

40% (0.02-0.16 mm)

*Dominant*: quartz

*Common:* feldspars

**Matrix**

40% Non-calcareous matrix, very dark brown with low optical activity.

**Voids**

10% Predominantly vughs (meso to macro); oriented parallelly to the walls.

**Comments**

This fabric is marked by rounded volcanic rocks composed of fine feldspars, namely plagioclase and some possible biotite, amphibole and epidote. The coarse fraction also, but less frequently, fragments of metamorphic rocks and minerals that could derive from acidic igneous plutonic rocks. This may indicate the use of a polymict tempering material.

| 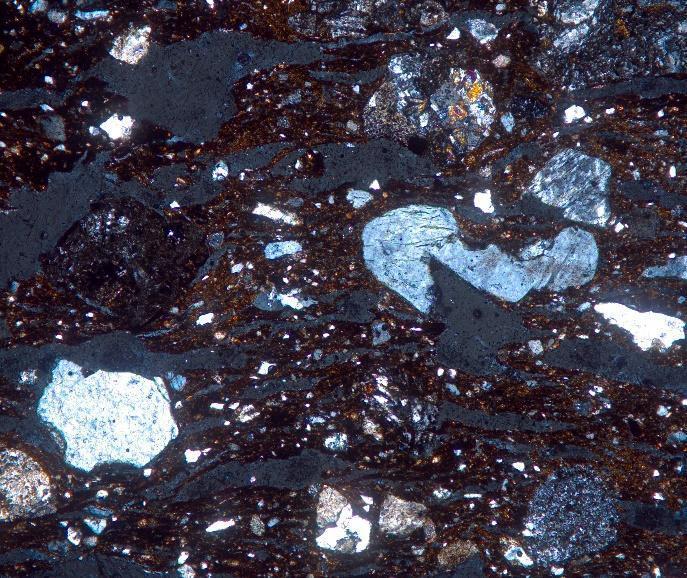 | 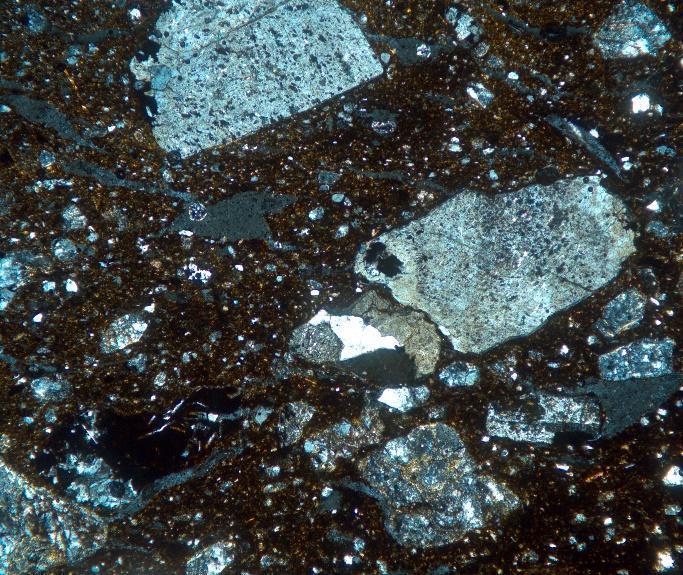 |
| --- | --- |
| TB-0025, 3 mm field of view | TB-0105, 3 mm field of view |

## Loners:

### Petrographic Fabric E-TB: Tempered with polymict sand.

TB-0060

**Inclusions**

30%; <2.08 mm; eq.-el.; a.-sr.; single to open spaced; strongly bimodal; well-sorted; randomly oriented.

**Coarse Fraction**

85% (0.20-2.08 mm)

*Frequent:* mudstone fragments; el. sr.-a. <2.08 mm, mode =1.44 mm.

*Frequent:* feldspars; eq.-el. a. <1.28 mm, mode=1.04. K-feldspar and perthite

*Few:* clastic sedimentary rock fragments; eq. sr. <1.68 mm, mode=1.04 mm. Composed primarily of quartz fragments. Possibly arenite.

*Very few:* sparitic calcite; eq. a. <0.48 mm, mode=0.48 mm.

**Fine Fraction**

15% (0.02-0.1 mm)

*Predominant:* feldspars

*Rare:* quartz

**Matrix**

50% light brown to beige, calcareous; surface areas darker brown; moderate optical activity.

**Voids**

20% planar voids (meso to macro); randomly oriented.

| 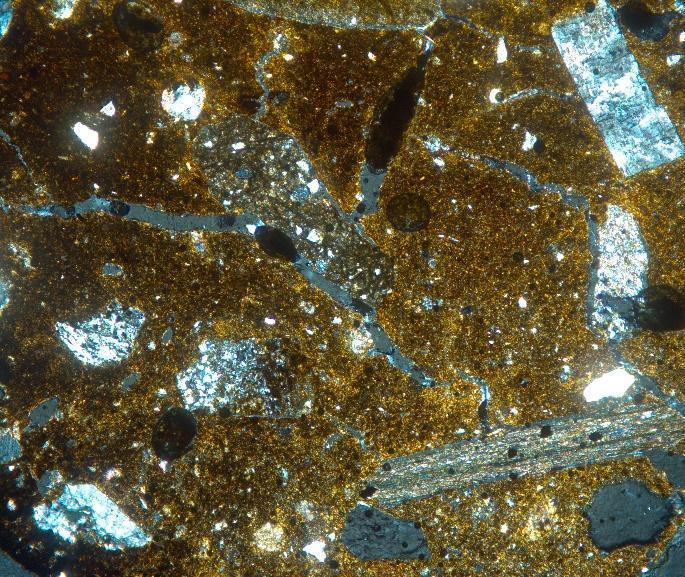 |  |
| --- | --- |
| TB-0060, 3 mm field of view |  |

**Comments**

This sample is characterized by strong bimodal distribution and with a coarse fraction with polymict composition. Maybe the tempering agent is fluvial sand.

### Petrographic Fabric F-TB: Tempered with metamorphic rocks fragments.

TB-0148

**Inclusions**

40%; <6.40 mm; eq.-el.; a.-sr.; single to double spaced; moderate bimodal; moderately sorted; randomly oriented.

**Coarse Fraction**

50% (0.32-6.40 mm)

*Dominant:* foliated metamorphic rock fragments; el. sr.-a. <6.40 mm, mode=0.72 mm. Composed of quartz and mica (biotite and muscovite).

*Common:* feldspars; eq.-el. a. <1.28 mm, mode=0.64 mm, K-feldspar, plagioclase and perthite.

*Common:* quartz; eq.-el. a. <0.64 mm, mode=0.24 mm.

*Very few:* biotite; el. a. <0.64 mm, mode=0.16 mm.

**Fine Fraction**

50% (0.02-0.16 mm)

*Frequent:* feldspars

*Frequent:* quartz

*Few:* opaques

*Very Few:* muscovite

**Matrix**

50% Very dark brown-to-black non-calcareous matrix, low optical activity.

**Voids**

10% predominantly vughs (micro to meso); randomly oriented.

**Comments**

This subfabric is bimodal and characterized by the presence of poorly sorted fragments of metamorphic rocks used as tempered. The fine fraction is moderately well sorted indicating that the clay has been fine or cleaned prior tempering.

| 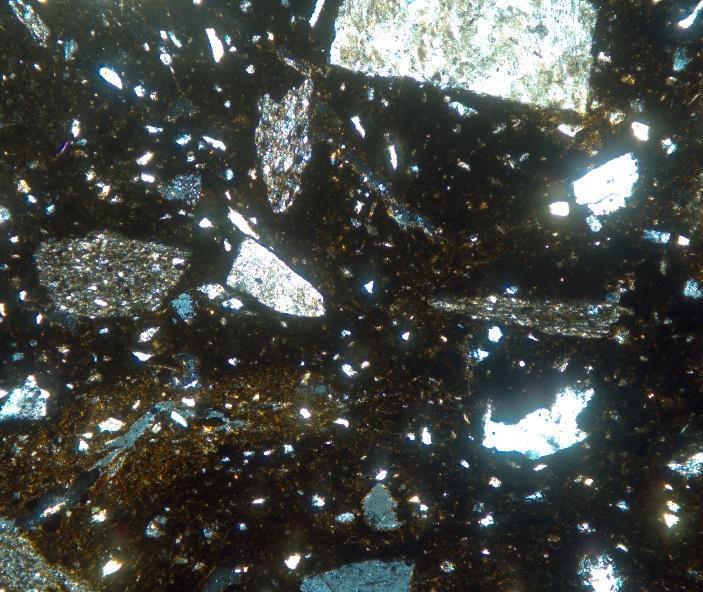 |  |
| --- | --- |
| TB-0148, 3 mm field of view |  |

# Begash

## Petrographic Fabric A-BG: Granite tempered.

BG-0506, BG-0544, BG-0566, BG-0782, BG-0881, BG-1035, BG-1052, BG-1067, BG-0899

**Inclusions**

30%; <4 mm; eq.-el.; a.-r.; single to open spaced; strongly bimodal; moderately sorted; randomly oriented.

**Coarse Fraction**

70% (0.24-4.00 mm)

*Dominant*: feldspars often weathered; eq.-el. a. <2.40 mm, mode=1.80 mm. K-feldspar, plagioclases, myrmekite, perthite.

*Common*: quartz; eq.-el. a.-sa. <2.00 mm, mode=0.48 mm.

*Common:* acidic igneous plutonic coarse-grained rock fragments; eq. a.-sa. <4.00 mm, mode=1.28 mm. Composed of quartz and feldspars (granite).

*Few:* micrite; eq.-el. sa.-r. <4.00 mm, mode = 0.48 mm (Frequent in BG 544 and BG 566)

*Rare:* epidote; eq. sa. <0.24 mm, mode = 0.16 mm.

*Very rare-absent:* sparitic calcite; eq. r. <0.80 mm, mode = 0.80 mm.

*Very Rare-absent:* biotite; eq.-el. a. <0.48 mm, mode=0.48 mm.

**Fine Fraction**

30% (0.02-0.08 mm)

*Dominant:* feldspars

*Dominant-Frequent:* quartz

*Few:* sparitic calcite

**Matrix**

50% non-calcareous, ranging from very dark brown to dark brown; moderate optical activity.

**Voids**

20 % predominantly channels and vesicle (micro to mega); generally oriented parallel to the walls.

**Comments**

Fabric group A exhibits strongly bimodal grain size distribution with a relatively well-sorted coarse fraction that consists primarily of rocks fragments composed of feldspars and quartz that display significant weathering into sericite. The distribution of grain sizes may indicate tempering using acidic plutonic rocks fragments (granite). The angularity and sorting of the coarse fraction in these samples may indicate that the rocks used as source of aplastic material were crushed and sieved prior to their use as tempers. The fine fraction is relatively well sorted and not very abundant, this could indicate that the clay used to produce this fabric could have fine or cleaned prior its use. In BG 566 the coarse fraction is less abundant and together with BG 544 contains abundant well-rounded inclusions of micrite in the coarse fraction, possibly indicating a different source of clay.

| 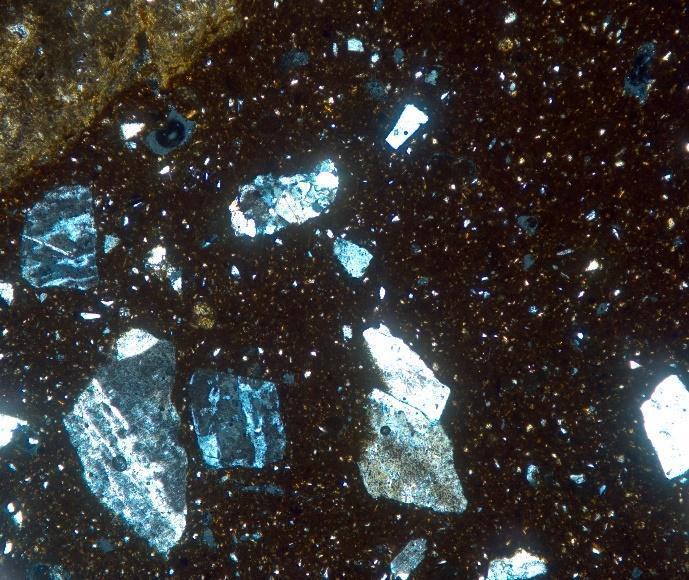 | 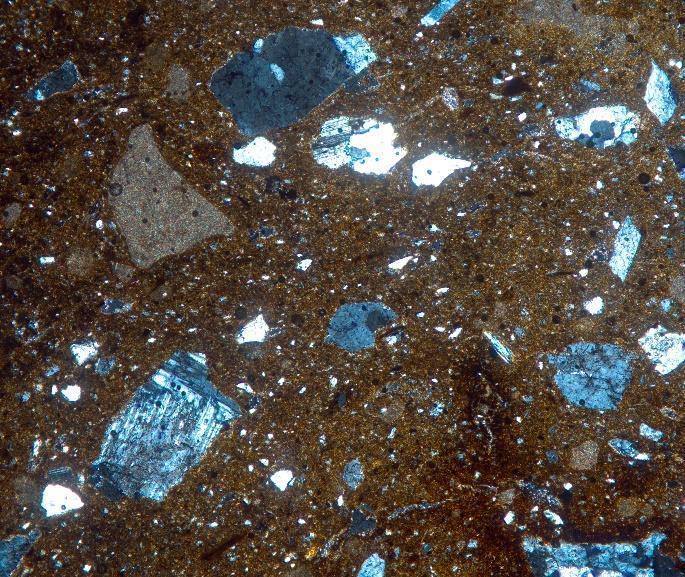 |
| --- | --- |
| BG-1052, 3 mm field of view | BG-0544, 3 mm field of view |

## Petrographic Fabric B-BG: Tempered with acidic-intermediate plutonic rock fragments.

BG-0071, BG-0843, BG-1015, BG-0779, BG-0068, BG-0786, BG-0731, BG-0711, BG-0773, BG-0045, BG-0776, BG-0963, BG-0722

**Comments**

Fabric B-BG shows a strong bimodal grain size distribution that is an indication of tempering. This fabric is marked by the presence of rock fragments composed of feldspars, quartz and a minor quantity of biotite and amphibole. The presence of biotite and other ferromagnesian minerals indicates that the rocks fragments and the minerals inclusions could derive from an igneous acidic to intermediate plutonic source.

### Subfabric B1-BG: Well sorted fine fraction

BG-0071, BG-0731, BG-0786, BG-0843, BG-1015, BG-0779, BG-0068

**Inclusions**

30-40%; <2.72 mm; eq.-el.; a.-sr.; close to open spaced; strongly bimodal; well-sorted; randomly oriented.

**Coarse Fraction**

70% (0.16-2.72mm)

*Dominant*: feldspars; eq.-el. a. <2.72 mm, mode=1.12 mm. K-feldspar, plagioclases, perthite. Feldspars sometimes show weathering into sericite.

*Common*: quartz; eq.-el. a.-sa. <1.52 mm, mode=0.32mm.

*Common:* acidic-intermediate igneous plutonic coarse-grained rock fragments; eq. a.-sa. <2.40 mm, mode=1.28 mm. Composed of quartz and feldspars, with some biotite and amphibole.

*Few:* biotite; eq.-el. a.-sa. <1.04 mm, mode=0.96 mm.

*Few:* micrite; eq.-el. sa.-r. <1.28 mm, mode=0.88 mm. (Abundant in BG 071 and BG 1015)

*Few-Rare:* amphibole; eq. sa. <0.80 mm, mode = 0.56 mm.

*Few-Absent:* sparitic calcite; eq. sr. <1.60 mm, mode=0.64 mm.

*Rare:* epidote; eq. a. <0.48 mm, mode=0.24 mm.

*Rare:* foliated metamorphic rock fragments; eq. a.-sa. <1.00 mm, mode=1 mm. Composed mainly of quartz.

**Fine Fraction**

30% (0.02-0.16 mm)

*Dominant:* feldspars

*Dominant-Frequent:* quartz

*Frequent:* micrite (abundant in BG 1015)

**Matrix**

50-60% non-calcareous matrix, ranging in color from dark brown to very dark brown; moderate optical activity.

**Voids**

10% mostly channels, planar voids and vughs (meso to macro); randomly oriented.

**Comments**

Subfabric 1 is marked by the presence of well-sorted fragments of acidic-intermediate igneous rocks composed of feldspars and minor quantities of biotite and amphibole. The fine fraction is not very abundant, and it is well sorted. This could indicate that clays could have been finer or cleaned before adding well sorted rock fragments as temper.

BG-0779: traces of combusted organic materials

BG-0068: more abundant amphiboles, [sparitic calcite](https://www.google.com/search?client=firefox-b-d&sca_esv=563665333&sxsrf=AB5stBjvq3Jwb1G5tN0tXODZqQdr6RSmWw:1694160986153&q=sparitic+calcite&spell=1&sa=X&ved=2ahUKEwj25pW-yZqBAxWShP0HHW0ACgwQkeECKAB6BAgIEAE) very weathered plagioclases

BG-0899: the fine fraction is less abundant

BG-0731 is similar to the rest of Fabric B but includes textural features (unhydrated lumps of clays)

BG-0786 exhibits a much darker matrix than the rest of Fabric B, possibly resulting from firing under reducing conditions. Additionally, this sample does not show the presence of calcite.

BG-0843: very weathered plagioclase

| 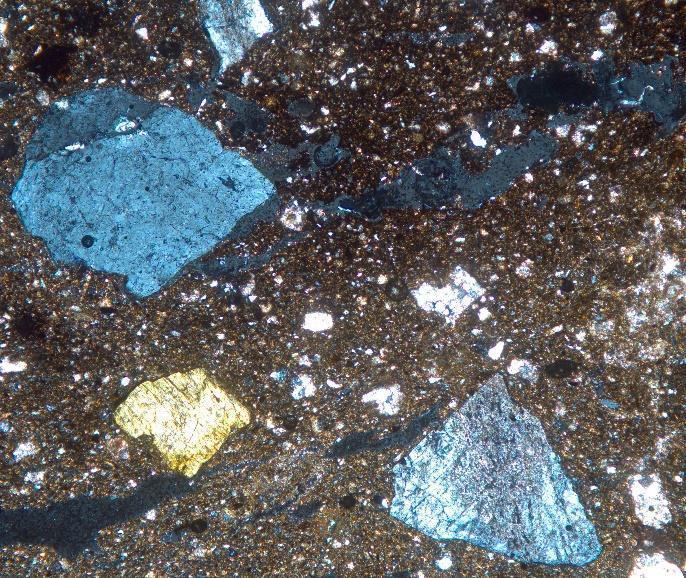 | 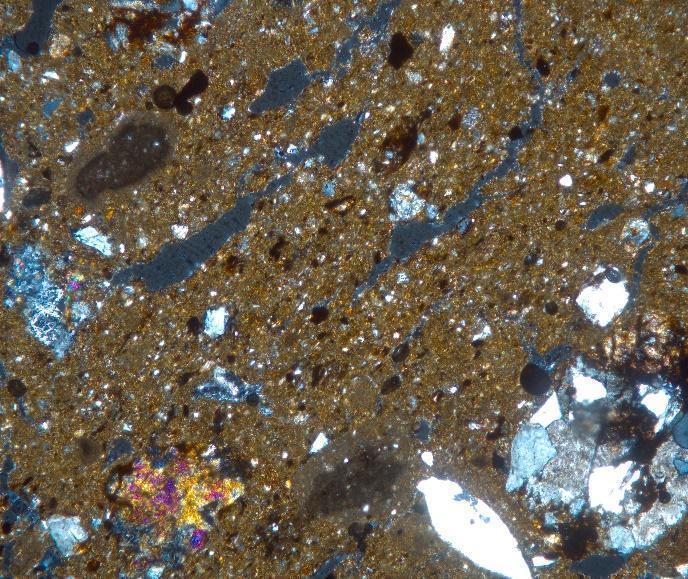 |
| --- | --- |
| BG-1015, 3 mm field of view | BG-0071, 3 mm field of view |
| 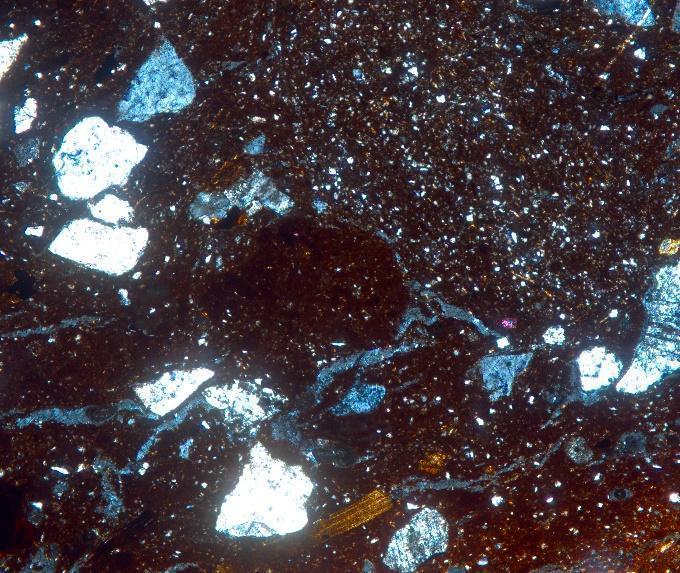 | 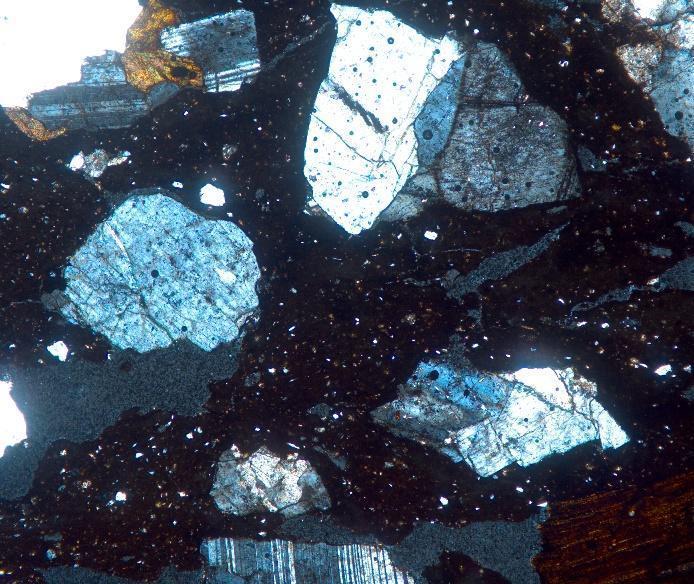 |
| BG-0731, 3 mm field of view | BG-0786, 3 mm field of view |

### Subfabric B2-BG: Poorly sorted fine fraction.

BG-0539, BG-0666, BG-0711, BG-0773, BG-0781; BG-0045, BG-0777, BG-0776, BG-0963, BG-0021, BG-0722

**Inclusions**

40%; <2.40 mm; eq.-el.; a.-r.; close to double spaced; weakly bimodal; poorly sorted; randomly oriented.

**Coarse Fraction**

60% (0.16-2.40 mm)

*Frequent:* feldspars; eq.-el. a.-sa. <2.24 mm, mode=0.88 mm. K-feldspar, plagioclases, perthite. Feldspars shows sometimes weathering into sericite.

*Common:* quartz; eq.-el. a.-sa. <2.32 mm, mode=0.40 mm.

*Few:* acidic-intermediate igneous plutonic coarse-grained rock fragments; eq. a.-sa. <2.40 mm, mode=1.60 mm. Composed of quartz and feldspars, with some biotite and amphibole.

*Few:* biotite; eq.-el. a.-sa. <0.8 mm, mode=0.24 mm.

*Few:* amphibole; eq. a.-sa. <1.68 mm, mode=0.40 mm.

*Very Few:* micrite; eq. r. <1.60 mm, mode=0.80 mm.

*Very Few:* sparitic calcite; eq.-el. sa. <1.76 mm, mode=0.88 mm.

*Very Few:* epidote; eq. sa. <0.48 mm, mode=0.16 mm.

*Rare:* foliated metamorphic rock fragments; eq. a.-sa. <1.00 mm, mode=1 mm. Composed mainly of quartz.

**Fine Fraction**

40% (0.02-0.16 mm)

*Dominant:* quartz

*Frequent:* biotite

*Frequent:* feldspar

*Few:* muscovite

**Matrix**

50% non-calcareous matrix, dark-brown in color; moderate optical activity.

**Voids**

10% channels and vughs (meso to macro); randomly oriented.

**Comments**

Subfabric 2 exhibits a coarser and less well sorted fine fraction. This may indicate the clay used to produce this fabric was coarser or less intensely cleaned than other examples of Fabric B.

BG-0776: sparitic calcite, common amphibole, weathered feldspars

BG-0963: zoned plagioclase

BG-0773: more abundant amphibole and epidote, more weathered feldspar (becoming sericite)

| 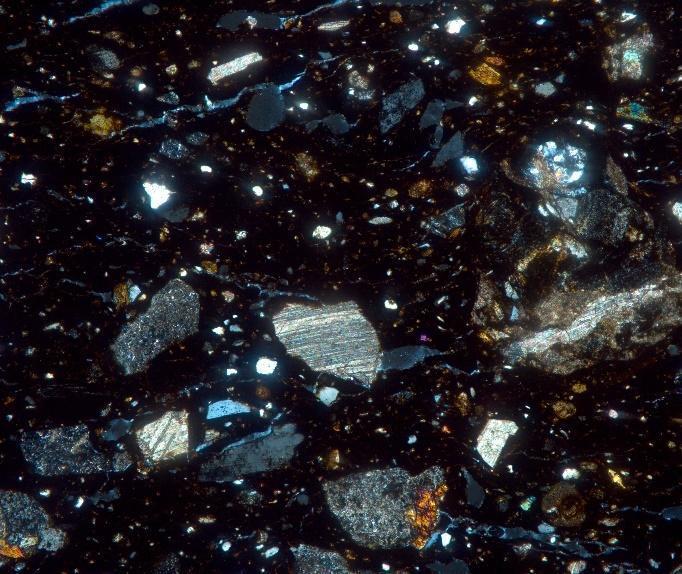 | 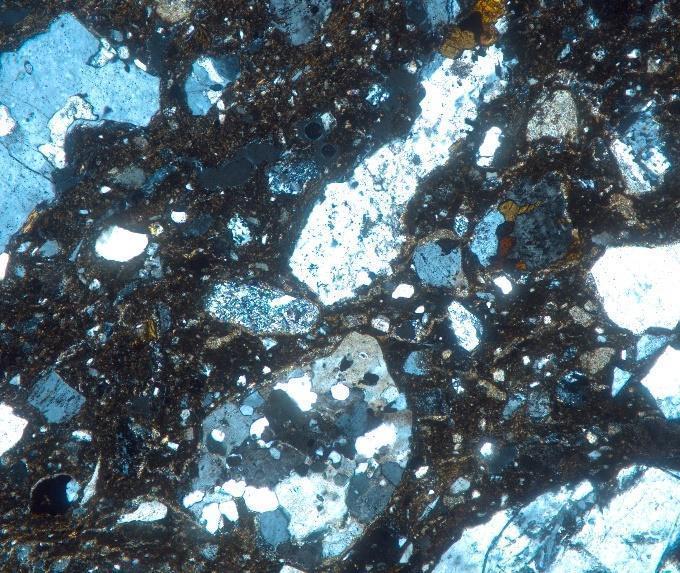 |
| --- | --- |
| BG-0776, 3 mm field of view | BG-0045, 3 mm field of view |

## Petrographic Fabric C-BG: Dark matrix and weakly bimodal.

BG-0769, BG-0012, BG-0833

**Inclusions**

30-40%; <1.84 mm; eq.-el.; a.-sr.; closed to single spaced; weakly bimodal; poorly sorted; randomly oriented.

**Coarse Fraction**

40% (0.32-1.84 mm)

*Dominant:* feldspars; eq.-el. sa. <0.88 mm, mode=2.00 mm. K-feldspar, plagioclases, perthite. Feldspars sometimes show weathering into sericite.

*Frequent:* quartz; eq. sa. <0.40 mm, mode=0.88 mm.

Few*:* amphibole; eq. sa. <0.88 mm, mode=0.24.

*Few:* micrite; eq. sr. <1.44 mm, mode=0.64 mm.

*Few:* biotite; eq.-el. a.-sa. <0.24 mm, mode=0.24 mm.

*Few to rare:* foliated metamorphic rock fragments; eq. a.-sa. <1.00 mm, mode=1 mm. Composed mainly of quartz

*Rare:* sparitic calcite; eq. sr. <0.72 mm, mode=0.48 mm.

*Rare:* chert; eq. sa.-sr. <1.20 mm, mode=0.92 mm

**Fine Fraction**

60% (0.04-0.16 mm)

*Dominant*: feldspar

*Common:* quartz

**Matrix**

50-60% non-calcareous matrix, very dark-brown in color; moderate optical activity.

**Voids**

10% channels with some planar voids and vughs (micro to macro); randomly oriented

**Comments**

This fabric is compositionally similar to fabric A-BG but stands out by a weaker bimodal grain size distribution and very dark matrix. Moreover, in this fabric we cannot observe the occurrence of plutonic rock fragments, but rare foliated metamorphic rock fragments composed of quartz have been observed. The coarse fraction is finer in comparison to Fabric A-BG and B-BG and less well sorted. The finer fraction is more abundant and coarser.

| 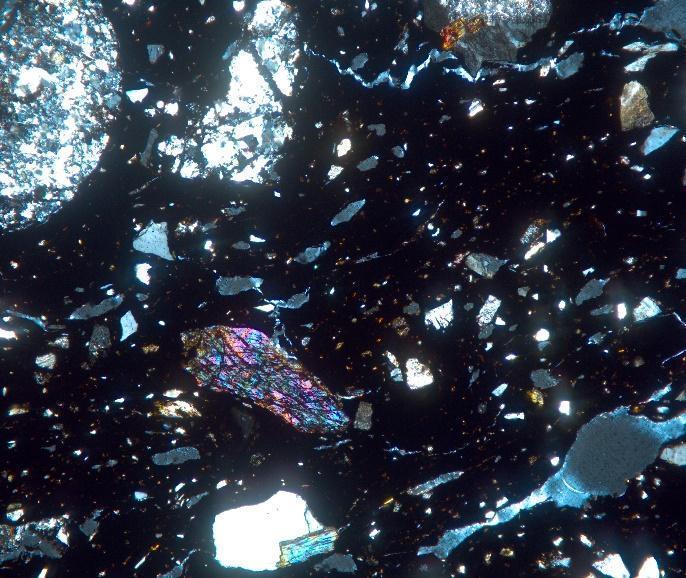 | 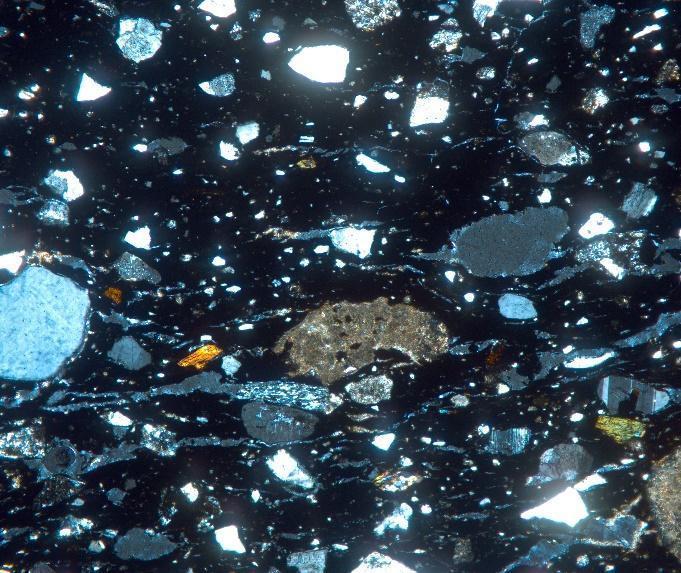 |
| --- | --- |
| BG-0012, 3 mm field of view | BG-0833, 3 mm field of view |

## Petrographic Fabric D-BG: Very weakly bimodal-to-polymodal fabric.

BG-0642, BG-0708, BG-1031

**Inclusions**

30%; -<1.68 mm; eq.-el.; a.-sr.; single to open spaced; weakly bimodal; moderately sorted; randomly oriented.

*Frequent:* feldspars; eq. sa. <1.84 mm, mode=0.96 mm. K-feldspar, plagioclases, perthite. Feldspars sometimes show weathering into sericite.

*Common:* quartz; eq. sa. <0.88 mm, mode=0.48 mm.

*Few:* intermediate-acidic igneous plutonic coarse-grained rock fragments; eq. sa. <1.68 mm, mode=0.80 mm. Composed of quartz and feldspars.

*Few:* sparitic calcite; eq. sa.-sr. <1.76 mm, mode=0.56 mm.

*Few:* amphibole; eq. sa. <0.80 mm, mode=0.48 mm.

*Few:* biotite; eq. sa. <0.80 mm, mode=0.48 mm.

*Very few:* micrite; eq.-el. sr. <1.60 mm, mode=0.64 mm. Often post-depositional.

*Very few:* sparitic calcite; eq. a. <1.84 mm, mode=0.96 mm.

*Few to rare:* foliated metamorphic rock fragments; eq. a.-sa. <1.00 mm, mode=1 mm. Composed mainly of quartz

**Matrix**

60% non-calcareous matrix, light brown to dark brown in color; strong optical activity.

**Voids**

10-20% predominantly vesicular voids with some planar voids (micro to macro); randomly oriented.

**Comments**

This fabric is compositionally similar to fabric B-BG but shows polymodal distribution that suggests that the inclusions might be naturally occurring or that clay was not well cleaned and the tempers not well sorted.

| 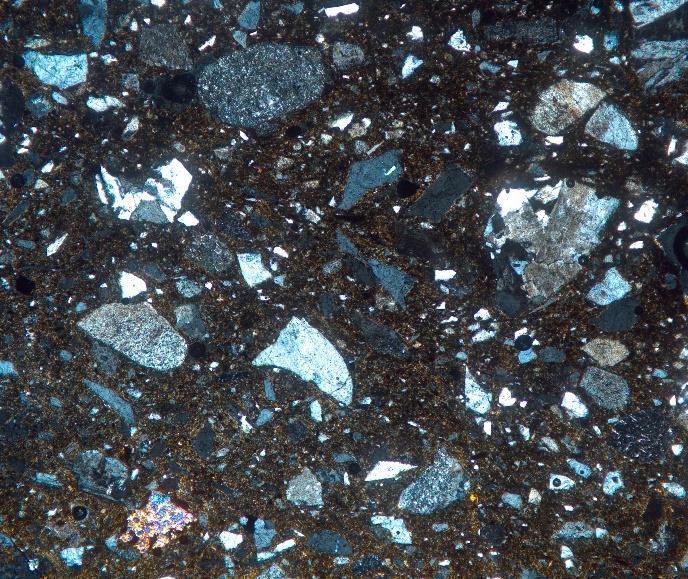 | 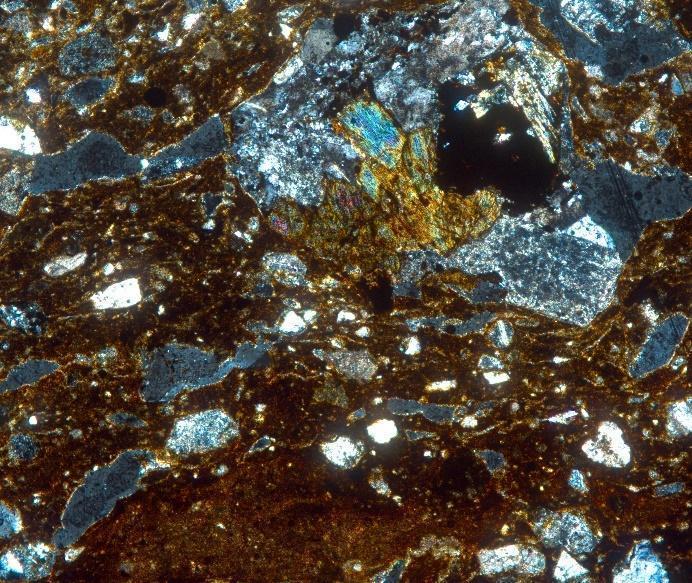 |
| --- | --- |
| BG-0642, 3 mm field of view | BG-1031, 3 mm field of view |

## Loners

### Petrographic Fabric E: Well-sorted granite tempered.

BG-0018

**Inclusions**

35%; <1.36 mm; eq.-el.; sa.-sr.; close to single spaced; strongly bimodal; well-sorted; randomly oriented.

**Coarse Fraction**

50% (0.17-1.36 mm)

*Dominant:* feldspars; eq.-el. sa. <1.36 mm, mode=0.96 mm. K-feldspar, plagioclases, perthite. Feldspars sometimes show weathering into sericite.

*Frequent:* quartz; eq. sa. <0.88 mm, mode=0.48 mm.

*Rare:* biotite; eq. sa.-sr. <0.24 mm, mode=0.16 mm.

**Fine Fraction**

50%(0.016-0.16 mm)

*Dominant*: quartz

*Common:* feldspars

*Rare:* amphibole

**Matrix**

55% non-calcareous dark brown matrix; moderate optical activity.

**Voids**

15% vughs (micro to macro); relatively parallel to the walls.

**Comments**

BG-0018 is compositionally very similar to fabric BG-A, but its coarse fraction is better sorted, and it is significantly smaller and more rounded in comparison to fabric BG-A. This might suggest a difference source for the tempering material.

| 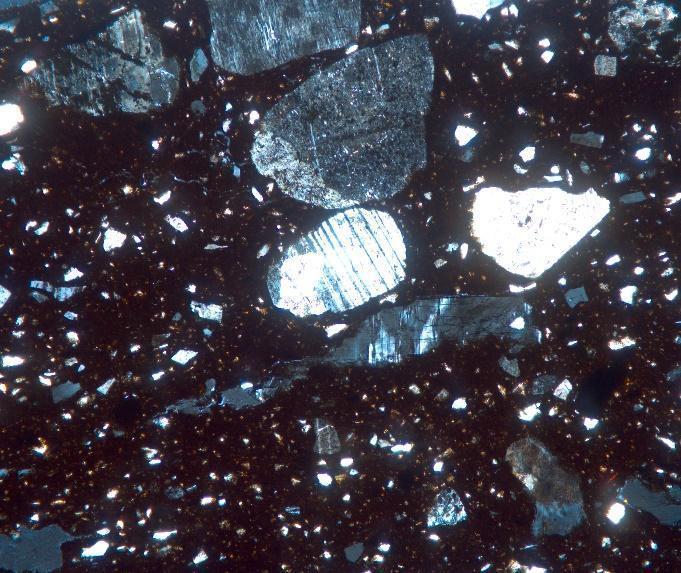 |  |
| --- | --- |
| BG-0018, 3 mm field of view |  |

### Petrographic Fabric F: Tempered with sand containing granite and metamorphic rock fragments.

BG-0990

**Inclusions**

30%; <2.24 mm; eq.-el.; sa.-r.; close to double spaced; strongly bimodal; well-sorted; randomly oriented.

**Coarse Fraction**

70% (0.1-2.24 mm)

*Dominant:* feldspars; eq.-el. sa.-sr. <0.80 mm, mode=0.56 mm. Includes k-feldspar and plagioclase.

*Common:* foliated metamorphic rock fragments composed of quartz and biotite; eq.-el.sa.-r. <0.56 mm, mode=0.40 mm

*Common:* quartz; eq. sa. <0.64 mm, 0.32 mm.

*Few:* acidic igneous plutonic fine-grained rock fragments; eq. sa. <1.52 mm, mode=0.48. Composed of quartz and biotite.

*Very few:* chert; eq. sa. <0.56 mm, mode=0.32 mm.

*Rare*: biotite; eq. sa. <0.24 mm, mode=0.16 mm.

**Fine Fraction**

30% (0.01-0.04 mm)

*Dominant:* quartz

*Frequent:* feldspars

**Matrix**

50% non-calcareous light brown matrix; strong optical activity.

**Voids**

20% channels and elongated vughs (micro to mega); loosely parallel to the walls.

**Comments**

BG-0990 exhibits a very fine and well-sorted fine fraction, that could indicate the use of a fine clay or cleaning of a clay. The coarse fraction is well sorted and consists of rounded acidic plutonic and metamorphic rock fragments that could have been added as temper. The roundness of the inclusions together with their polymict nature of the coarse fraction might suggest the use of a fluvial sediment for tempering.

| 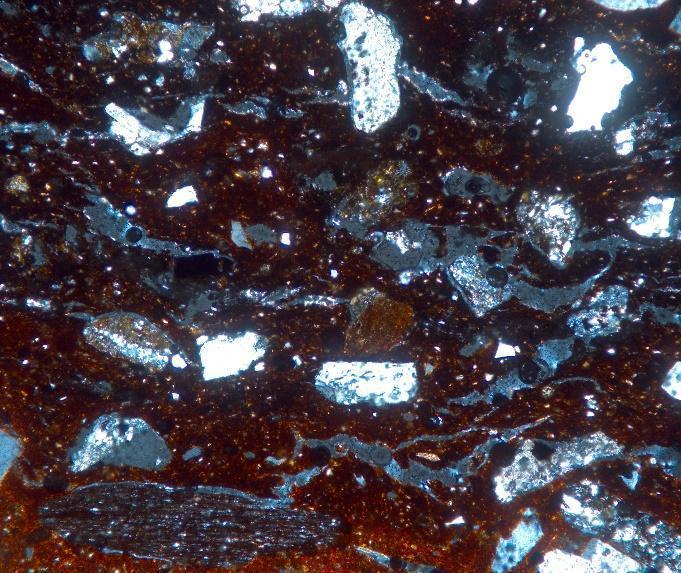 |  |
| --- | --- |
| BG-0990, 3 mm field of view |  |

### Petrographic Fabric G: Tempered with sand containing metamorphic and volcanic rock fragments.

BG-0067

**Inclusions**

20%; <1.36 mm; eq.-el.; a.-sa.; single to open spaced; strongly bimodal; well- sorted; randomly oriented.

**Coarse fraction**

70 % (0.08-1.36 mm)

*Dominant:* feldspars; eq.-el. a.-sa. <1.28 mm, mode=0.40 mm. Includes heavily weathered plagioclase and k-feldspar.

*Common:* foliated metamorphic rock fragments; eq. sa. <0.56 mm, mode=0.48 mm. Composed of quartz.

*Few:* igneous volcanic basic/intermediate fine-grained rock fragments; eq. sa. <0.56 mm, mode=0.48 mm. Composed of feldspars and pyroxenes with hyalopilitic groundmass.

*Few:* mudstone fragments; eq. sa. <0.64 mm, mode=0.56 mm.

*Rare:* sparitic calcite; el. sa. <0.56 mm, mode=0.24 mm.

*Very Rare:* biotite; eq. sa. <0.48 mm, mode=0.48 mm.

**Fine fraction**

30% (0.01-0.08 mm)

*Dominant*: quartz

*Frequent*: feldspars

*Common*: opaques

*Very few*: muscovite

**Matrix**

70%; non-calcareous base clay, light brown in color with high to medium optical activity.

**Voids**

10% vughs (micro to macro); randomly oriented.

**Comments**

BG-0067 is strongly bimodal with a very well-sorted fine and coarse fraction. This implies an accurate cleaning of clay and preparation of tempering material. The presence of metamorphic and volcanic rock fragments makes this fabric different in comparison to the others.

| 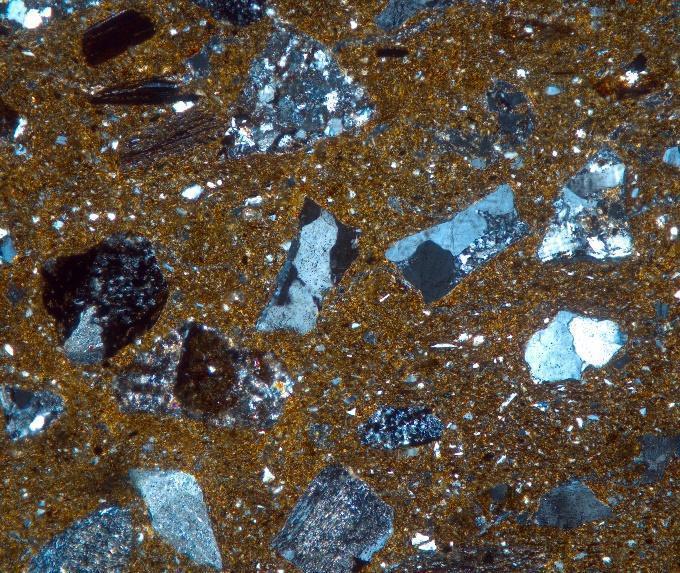 |  |
| --- | --- |
| BG-0067, 3 mm field of view |  |

### Petrographic Fabric H: Tempered with flint and metamorphic rock fragments

BG-1049

Inclusions

40%; <2.80 mm; eq.-el.; a.-r.; single to double spaced; weakly bimodal; poorly sorted; randomly oriented.

**Coarse Fraction**

50% (0.24-2.80 mm)

*Dominant:* feldspars; eq.-el. a.-sa. <1.36 mm, mode=0.72 mm. Weathered plagioclase and k-feldspars, and perthite.

*Frequent*: quartz; eq.-el. a.-sa. <2.80 mm, mode=1.28.

*Common:* flint; el. r. <2.24 mm, mode=1.60 mm.

Few*:* foliated metamorphic rock fragments; el. r. <2.24 mm, mode=1.60 mm. Composed of quartz.

*Few:* acidic igneous plutonic medium-grained rock fragments. el. sa.-sr. <1.76 mm, mode=1.76mm. Composed primarily of quartz and feldspars and a minor quantity of amphibole.

*Rare:* sparitic calcite; el. a.-sa. <1.28 mm, mode=1.28 mm.

*Very Rare:* micrite; eq. r.-sr. <0.88 mm, mode=0.88 mm.

**Fine Fraction**

50% (0.04-0.24 mm)

*Dominant:* quartz

*Frequent:* feldspars

*Few:* biotite

**Matrix**

50% non-calcareous matrix; strong optical activity.

**Voids**

10% vughs (micro to macro); randomly oriented.

**Comments**

BG-1049 is weakly bimodal and marked by big and not well sorted fragments of metamorphic rocks alongside flint and few fragments of acidic plutonic rocks. This suggests that the clay was not well cleaned and the tempering agented were not well sorted.

| 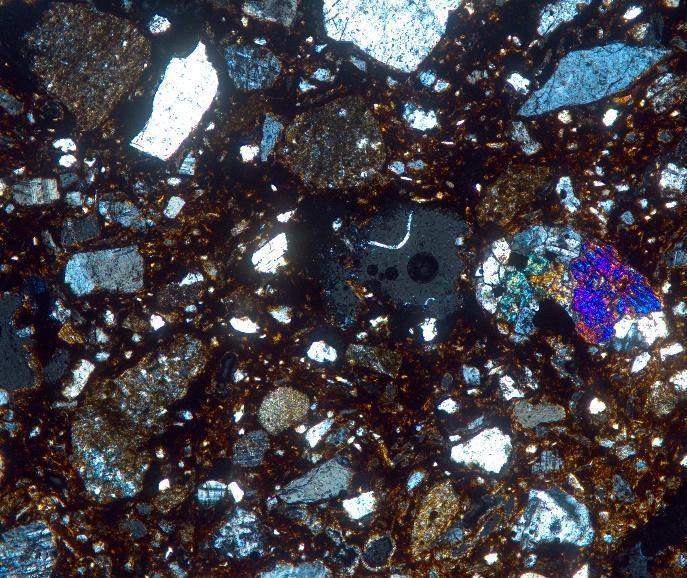 |  |
| --- | --- |
| BG-1049, 3 mm field of view |  |

### Petrographic Fabric I: Tempered with grog

BG-3001

**Inclusions**

40%; <2.08 mm; eq.-el.; a.-sa.; single to double space; polymodal; poorly sorted; randomly oriented.

*Dominant:* feldspars; eq. sa. <2.08 mm, mode=0.40 mm. Plagioclases, k-feldspar, perthite, very weathered.

*Frequent:* quartz; eq. sa. <0.48 mm, mode=0.32 mm.

Common: grog (containing moderately sorted quartz and feldspars,); eq. sa. <1.68 mm, mode=0.80 mm. Characterised by clear boundaries, high optical density, and discordance within the fabric.

Few*:* amphibole; eq. sa. <0.80 mm, mode=0.48 mm.

Few: amphibole; eq. sa. <0.80 mm, mode=0.48 mm.

*Few:* biotite; el.-eq. a. <0.88 mm, mode=0.64 mm.

*Few:* sparitic calcite; eq. a. <0.88 mm, mode=0.48 mm.

**Matrix**

50% non-calcareous matrix; moderate optical activity.

**Voids**

10% vughs and planar voids (micro to mega); randomly oriented.

**Comments**

The polymodal grain size distribution points towards the addition of grog to an uncleaned clay.

| 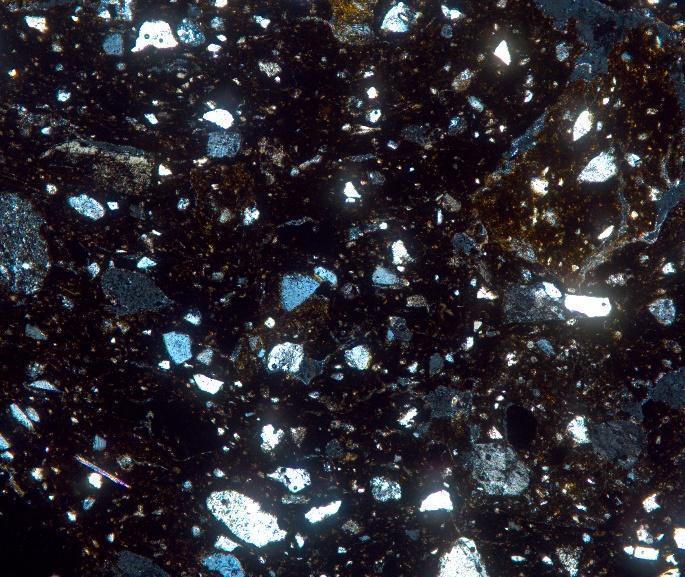 |  |
| --- | --- |
| BG-3001, 3 mm field of view |  |

# Kuigan

## Petrographic fabric A-KG: Granite tempered

KG-1, KG-2, KG-4, KG-7, KG-3, KG-5, KG-9, KG-10

### Subfabric A1-KG: Bimodal

KG-1, KG-2, KG-3, KG-4, KG-5, KG-7, KG-9

**Inclusions**

30%; <2.3 mm; eq.-el.; a.-sr.; close to open spaced; weakly bimodal; poorly sorted; randomly oriented.

**Coarse Fraction**

60% (0.24-2.32mm)

*Common:* acidic plutonic coarse-grained rock fragments; el. a.-sa. < 2.32 mm, mode=0.96 mm. Composed of plagioclase, k-feldspars, perthite, and sometimes with amphibole, mica and opaque minerals (granite).

*Few:* feldspars; el. a. < 0.80 mm, mode=0.40 mm.

*Few:* quartz; eq. a. < 0.16 mm, mode=0.08 mm.

*Rare:* amphibole; el. sa.-sr. < 0.96 mm, mode=0.32 mm.

*Rare:* calcite; eq.-el. sa.-sr. < 1.20 mm, mode=0.32 mm.

*Very rare:* epidote; eq. sa.-sr. < 0.40 mm, mode=0.24 mm,

**Fine Fraction**

(40% 0.05-0.1 mm)

*Frequent:* feldspars

*Common:* biotite

*Few:* pyroxenes

**Matrix**

60% non-calcareous matrix. Reddish/brownish to yellowish/brownish in XP and PPL. Medium high to high optical activity visible.

**Voids**

10% meso and macro channels, as well as meso vughs. Channels parallel to the edges. < 3.20 mm.

**Comments**

This subfabric contains acidic igneous plutonic rock fragments (granite). In addition, feldspar, quartz, amphibole, calcite, and rarely epidote can be observed. The bimodality and the general moderately well sorted grain size distribution of the inclusions could indicate that the clay was cleaned prior to its usage and tempered with granite. This subfabric is coarser in comparison to subfabric A1KG.

| 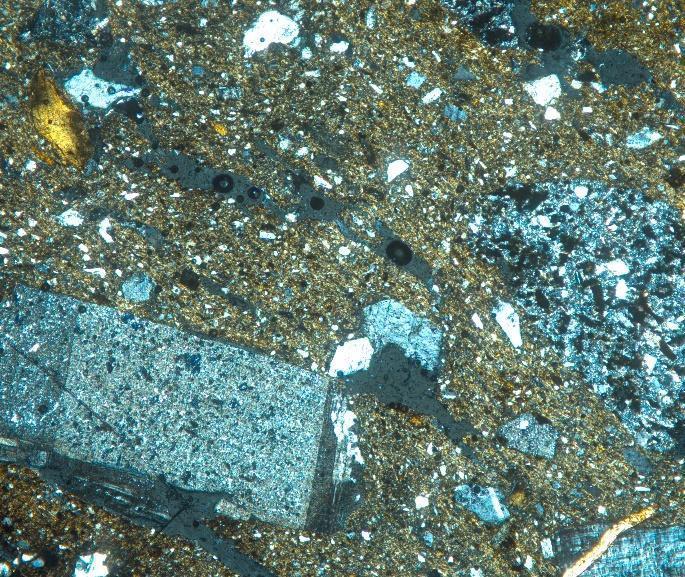 | 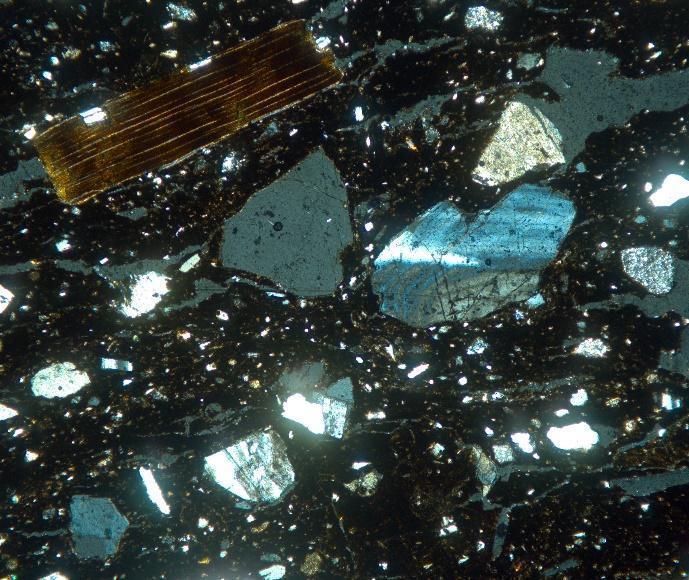 |
| --- | --- |
| KG-2, 3 mm field of view | KG-9, 3 mm field of view |

### Subfabric A2-KG: Strongly bimodal

KG-10

**Inclusions**

30-40%; <3.60 mm; eq.-el.; a.-sr.; strongly bimodal; single to double spaced; randomly oriented.

**Coarse Fraction**

50 % (-0.40-3.60 mm)

*Frequent:* feldspars (Orthoclase intergrown with perthite, plagioclase); el. sa. < 3.60 mm, mode=0.64 mm

*Very few:* acidic intrusive coarse-grained rock; el. sa.-sr. < 2.80 mm, mode=2.76 mm. Plagioclase, k-feldspars, perthite, and sometimes with amphibole, biotite and opaques (granite).

*Very few:* quartz; eq. a. < 0.16 mm, mode=0.08 mm.

*Rare:* amphibole; el. sr. < 0.96 mm, mode=0.40 mm,

*Rare:* opaque minerals; el. sa.-sr. < 1.44 mm, mode=0.56 mm.

*Very rare:* calcite; el. sa.-sr.< 2.00 mm, mode=0.32 mm.

**Fine Fraction** 50% (0.16-0.32 mm)

*Dominant*: quartz

*Common*: feldspars

*Few*: muscovite

**Matrix**

50% non-calcareous matrix. Yellowish/brownish in XP and PLL. High optical activity visible.

**Voids**

10–20% meso and macro vughs, some secondary calcite in these vughs; randomly oriented.

**Textural features**

no argillaceous inclusions identifiable

**Comments**

This subfabric contains acidic igneous plutonic rock fragments (granite). In addition, feldspars, quartz, and amphibole can be observed. The bimodality and the general moderately well sorted grain size distribution of the inclusions could indicate that the clay was cleaned prior to its usage and tempered with coarse fragments of granite.

| 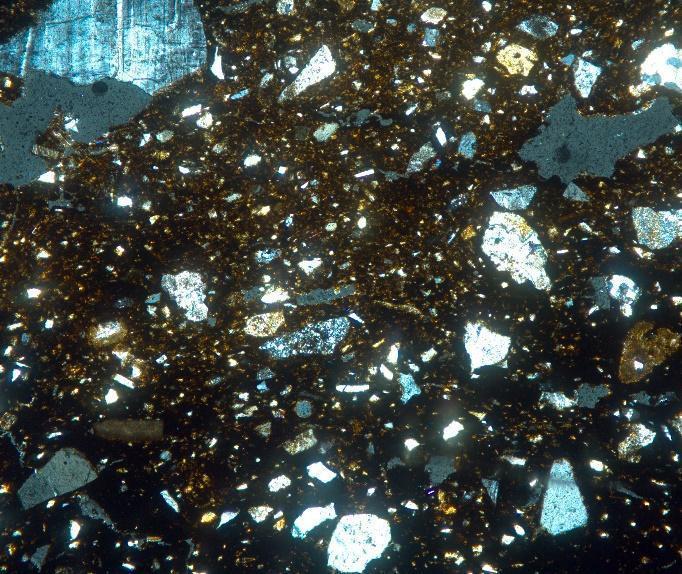 |  |
| --- | --- |
| KG-10, 3 mm field of view |  |

## Loners:

### Petrographic fabric B-KG: With polymict sand

KG-8

**Inclusions**

45%; <2.08 mm; eq.-el.; a.-r.; close to single spaced; weakly bimodal; poorly sorted; randomly oriented.

**Coarse Fraction**

60 % (0.1-2.08 mm)

Dominant*:* quartz; eq. a. < 0.16 mm, mode=0.08 mm.

*Frequent:* feldspars; el. a.-sr. < 0.96 mm, mode=0.40 mm. Plagioclase.

*Few:* muscovite; el. a.-sa. < 0.32 mm, mode=0.16 mm.

*Very few:* chert; el. r. < 2.08 mm, mode=0.96 mm.

*Very few:* acidic igneous volcanic fine-grained rock fragments with a porphyritic microstructure and phenocrystals of plagioclase (rhyolite?); eq. sr.-r.< 0.96 mm, mode=0.56 mm.

*Very few:* acidic igneous plutonic coarse-grained rock fragments; el. sa.-sr. < 1.20 mm, mode=0.56 mm. Composed of (plagioclase, k-feldspars, perthite, and sometimes with amphibole mica and opaque minerals (granite).

*Rare:* foliated metamorphic rockseq. sr.-r.< 0.96 mm, mode=0.56 mm. Composed of quartz.

*Rare:* opaque minerals; el. a.-sr.< 1.20 mm, mode=0.64 mm.

*Rare:* calcite; el. sa.-sr. < 0.64 mm, mode=0.32 mm.

**Fine fraction**

40% (0.01-0.1 mm)

Dominant: quartz/feldspars

Common: muscovite

**Matrix**

50% non-calcareous matrix. In XP and PPL blackish/brownish in the core and reddish/brownish at one side of the edges. Medium high optical activity visible.

**Voids**

5% Vughs (meso); randomly oriented.

**Comments**

The polymodality and diverse inclusion assemblage with rounded grains might suggest that a fluvial sediment might be the raw material to produce this sample.

| 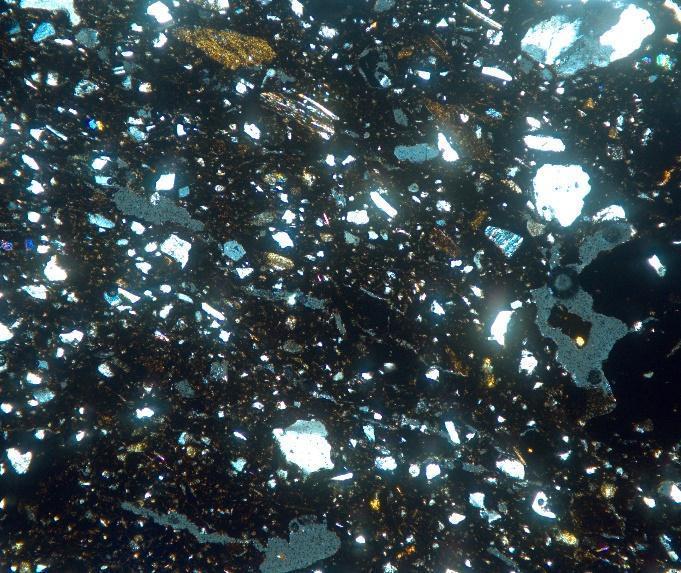 |  |
| --- | --- |
| KG-8, 3 mm field of view |  |

### Petrographic fabric C-KG: Polymodal fabric

KG-6

**Inclusions**

30%;<1.44 mm; eq.-el.; a.-sa.; single to open spaced; polymodal; poorly sorted; randomly oriented.

*Frequent:* feldspars; el. a.-sa. (< 1.44 mm), mode=0.32 mm. Microcline, orthoclase.

*Very few:* quartz; eq. a. < 0.16 mm, mode=0.08 mm.

*Rare:* muscovite; el. a.-sa.< 0.32 mm, mode=0.16 mm.

*Rare:* biotite; eq.-el. sa.< 0.24 mm, mode=0.08 mm.

*Very rare:* acidic igneous plutonic coarse-grained rock fragments; el. sa. < 0.48 mm, mode=0.24 mm Composed of plagioclase, k-feldspars, perthite, and sometimes with amphibole (hornblende), mica and opaque minerals (granite).

**Matrix**

60% non-calcareous matrix. Blackish/brownish in XP and PPL. Medium high to low optical activity visible.

**Voids**

10% meso and macro elongated vughs; some vughs with secondary calcite.

**Comments**

This fabric is characterized by feldspar inclusions. Additionally, quartz, muscovite, biotite and acidic igneous plutonic rock fragments originating (granite) can be observed. The polymodal distribution suggest the inclusion might be naturally occurring.

| 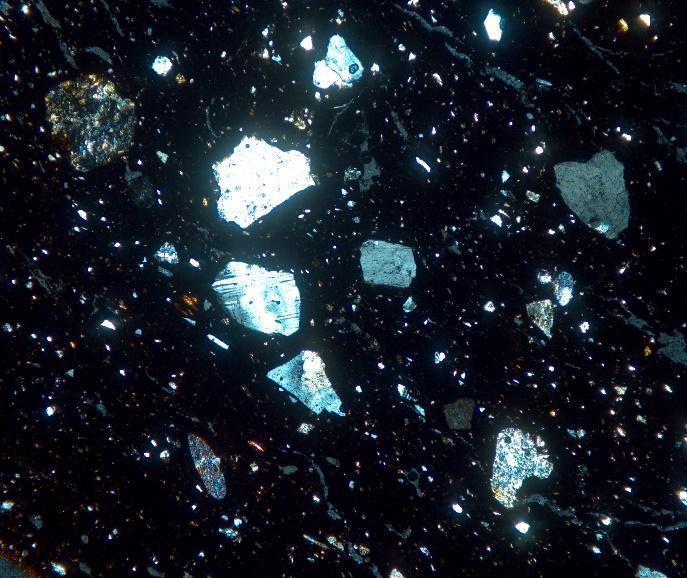 |  |
| --- | --- |
| KG-6, 3 mm field of view |  |

# Talapty

## Petrographic fabric A-TL: Granite tempered

TL-1, TL-4, TL-5

**Inclusions**

30-40%; <3.13 mm; eq.-el.; a.-r.; single to double spaced; strongly bimodal; moderately sorted; randomly oriented.

**Coarse Fraction**

50%, (0.24-3.12 mm)

*Dominant:* feldspars < 1.40 mm, mode=0.64 mm. el. a.-sa. Plagioclase, perthite, k-feldspars.

*Few:* quartz; eq. a.-sa. < 0.16 mm, mode=0.08 mm.

*Few:* acidic igneous plutonic coarse-grained rock fragments; el. sa. < 3.12 mm, mode=1.12 mm. Composed of plagioclase, k-feldspars, perthite, muscovite, amphibole (granite).

*Very few:* muscovite; el. sa. < 0.16 mm, mode=0.08 mm.

*Very few:* biotite; sa.-sr. < 0.16 mm, mode=0.08 mm.

*Rare:* amphibole; el. sa. < 1.04 mm, mode=0.48 mm (in TL 1 more present).

*Rare:* chert; el. sr. < 0.56 mm, mode=0.32 mm (in TL 1 more present).

*Very rare:* calcite; el. sa.-r. < 1.04 mm, mode=0.24 mm, (in TL 1 more present).

**Fine Fraction**

50% (0.01-0.16 mm)

*Dominant*: quartz

*Frequent*: feldspars (Plagioclase)

*Few*: muscovite

**Matrix**

40-50% non-calcareous matrix. Brownish/blackish in XP, Brownish/blackish to yellowish/brownish and reddish/brownish in PPL. Low optical activity visible.

**Voids**

20% meso and macro vughs. Parallel to the edges of the pottery fragments.

**Comments**

The bimodal grain size distribution and the fine fraction indicate that clay might have been cleaned before being tempered.

| 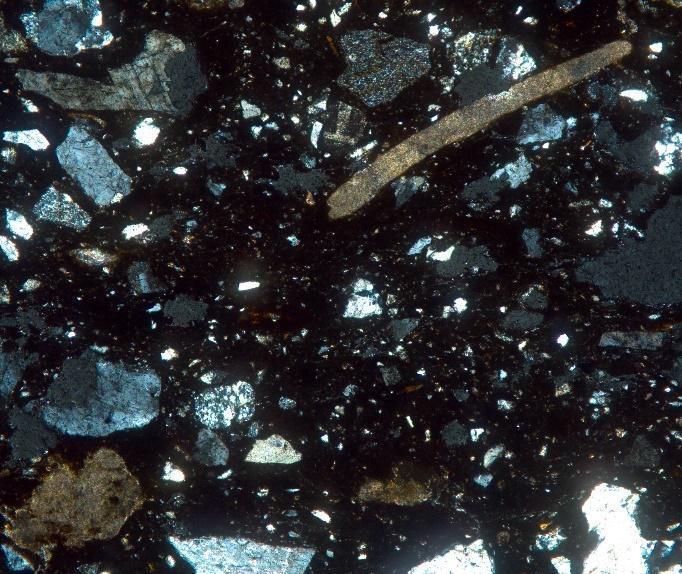 | 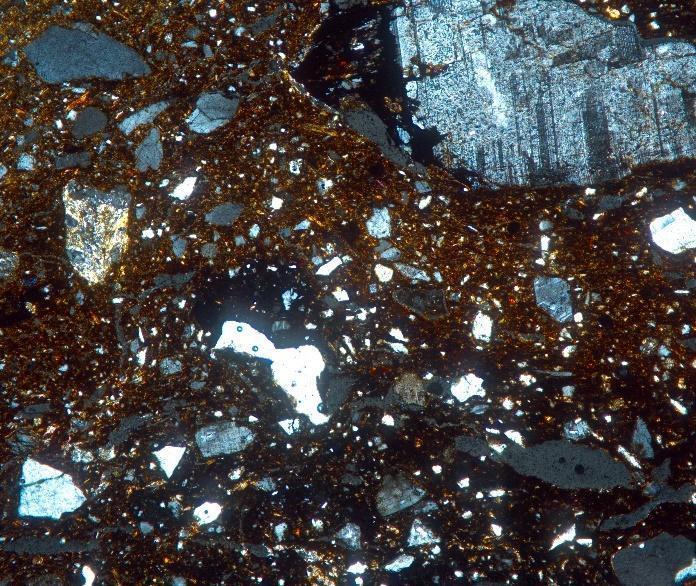 |
| --- | --- |
| TL-1, 3 mm field of view | TL-4, 3 mm field of view |

## Loners:

### Petrographic fabric B-TL: Grog tempered

TL-2

**Inclusions**

30-40%; <1.68 mm; eq.-el.; a.-sa.; single to open spaced; weakly bimodal; poorly sorted; randomly oriented.

**Coarse Fraction**

40% (0.08-1.68 mm)

*Frequent:* feldspars; el. a. < 1.12 mm, mode=0.64 mm. Plagioclase, perthite, k-feldspars).

*Common:* grog (containing moderated soted feldpars and quartz); eq.-el. sa.-a. <1.36 mm, mode=0.88 mm. Often very dark brown to black, fired under reducing conditions. Grog particles have low optical density, clear to merging boundaries and discordance.

*Few:* acidic igneous plutonic coarse-grained rock fragments; el. a.-sa. < 1.68 mm, mode=0.88 mm. Composed of plagioclase, k-feldspars, perthite, amphibole, biotite (granite).

*Few:* quartz; eq. a.-sa. < 0.16 mm, mode=0.04 mm.

*Rare:* calcite; el-eq. sa. < 0.64 mm, mode=0.08 mm.

*Rare:* amphibole; el. a. < 0.56 mm, mode=0.24 mm.

*Very rare:* chert; el. sa.< 0.56 mm, mode: 0.32 mm.

**Fine fraction**

60% (0.01-0.08 mm)

Dominant: quartz

Frequent: feldspars

Rare: muscovite

**Matrix**

50% non-calcareous matrix. Brownish to blackish in XP and PPL. Low to medium high optical activity.

**Voids**10-20% Meso and macro vughs; randomly oriented.

**Comments**

TL-2 is compositionally similar to fabric group A-TL, the distinguishing factor is the addition of grog. The poor sorting of the inclusions might indicate that the base clay was not well cleaned before adding grog.

| 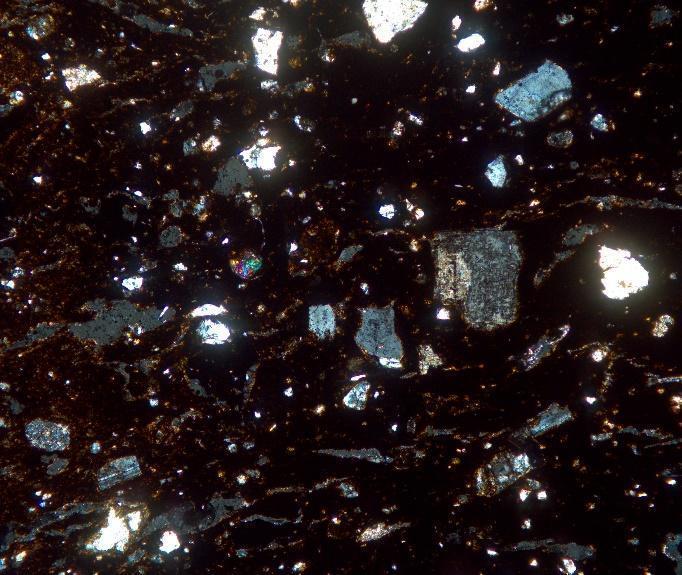 |  |
| --- | --- |
| TL-2, 3 mm field of view |  |

### Petrographic fabric C-TL: Tempered with polymict sand

TL-3

Inclusions

30%; <1.84 mm; eq.-el.; a.-r.; close to open spaced; bimodal; moderately sorted; randomly oriented.

**Coarse Fraction**

70% (0.56-1.84 mm)

*Frequent:* feldspars; eq.-el. a.-sa. < 0.96 mm, mode=0.56 mm. Plagioclase, microcline, perthite.

*Few:* foliated metamorphic rock fragments; el. a.-sa. < 1.2 mm, mode=0.48 mm. Composed of quartz and muscovite.

*Few:* quartz; eq. a.-sa. < 0.96 mm, mode=0.56 mm.

*Few:* acidic igneous plutonic coarse-grained rock fragments; eq.-el. a.-sa. < 1.44 mm, mode=0.88 mm. Composed of plagioclase, k-feldspar, perthite, biotite (granite).

*Few:* basic igneous volcanic fine-grained rock fragments with a porphyritic microstructure and phenocrystals of plagioclase (probably basalt); el. sa. < 1.6 mm, mode=0.9 mm.

*Rare:* chert; eq.-el. sa.-sr. < 1.84 mm, mode=0.56 mm.

*Rare:* calcite; eq.-el. sr.-r. < 0.80 mm, mode=0.48 mm.

**Fine Fraction**

30% (0.1-0.24 mm)

*Dominant*: quartz

*Common*: feldspars

**Matrix**

50% non-calcareous matrix. Reddish/brownish to blackish brownish in XP and reddish/brownish to yellowish/brownish in PPL. Height to medium high optical activity visible.

**Voids**

20% vughs (meso to macro), and channels (micro). Orientation mostly parallel to the edges of the fragment.

**Comments**

The bimodal grain size distribution indicates that a polymict sand was added as temper.

| 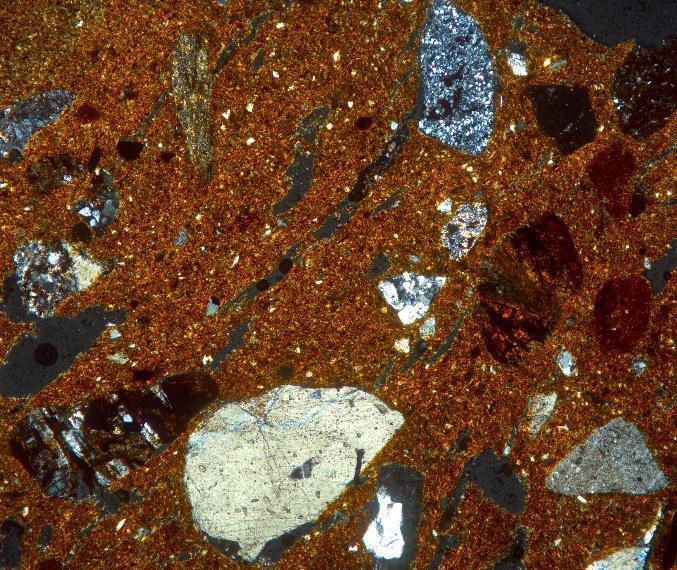 |  |
| --- | --- |
| TL-3, 3 mm field of view |  |

# Tamgaly

## Petrographic fabric A-TM: Granite tempered

TM-4, TM-6

### Subfabric A1-TM: Weakly bimodal

TM-4

**Inclusions**

40%; <2.00 mm; eq.-el.; a.-sr.; close to single spaced; weakly bimodal; moderately sorted; randomly oriented.

**Coarse Fraction**

30% (0.24-2.00 mm)

*Dominant:* quartz; eq. a. < 0.16 mm, mode=0.08 mm.

*Frequent:* acidic igneous coarse-grained rock fragments; el. sa.-sr. <2.00 mm, mode=0.88 mm. Composed of plagioclase, k-feldspars, perthite and sometimes biotite (granite).

*Common:* feldspars; el. a.-sa. < 0.48 mm, mode=0.24 mm. Orthoclase.

*Rare:* chert; eq.-el. sa.-sr. < 1.44 mm, mode=0.40 mm.

*Very rare:* calcite; eq.-el.. sr. < 0.72 mm, mode=0.32 mm.

**Fine Fraction**

70% (0.04-0.24 mm)

*Dominant*: quartz

*Frequent*: feldspars

*Common*: muscovite

**Matrix**

50% non-calcareous matrix. Blackish to brownish in XP and PPL. Medium high optical activity visible.

**Voids**

10%, meso and macro vughs; elongated to equant. Some show secondary calcite randomly oriented

**Comments**

The fine fraction is abundant, but well sorted, this could indicate that the clay was cleaned prior to being tempered with fragments of an acidic igneous plutonic rock (granite).

| 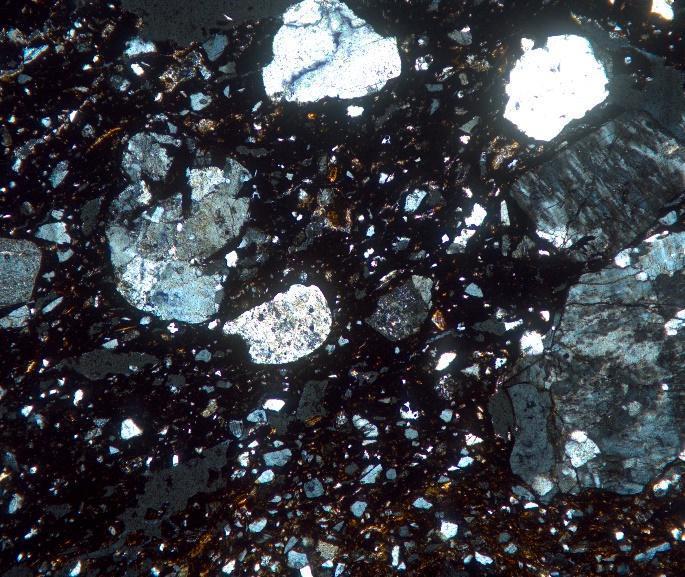 |  |
| --- | --- |
| TM-4, 3 mm field of view |  |

### Subfabric A2-TM: Strongly bimodal

TM-6

**Inclusions**

30%; <3.20 mm; eq.-el.; a.-sr.; close to open spaced; strongly bimodal; moderately sorted; randomly oriented.

**Coarse Fraction**

60% (0.8-3.20 mm)

*Dominant:* acidic igneous plutonic coarse-grained rock fragments; el. a.-sa. < 3.20 mm, mode=1.20 mm. Composed of plagioclase, k-feldspars, perthite, and sometimes amphibole, biotite and opaque (granite).

*Common:* feldspars; eq. a.< 1.20 mm, mode=0.8 mm.

*Common:* quartz; eq. a.< 1.20 mm, mode=0.8 mm.

*Rare:* calcite; el. sr.< 0.32 mm, mode=0.24 mm.

*Rare:* opaque minerals; el. a.< 0.24 mm, mode=0.16 mm.

**Fine Fraction**

40% (0.01-0.04 mm)

*Frequent:* quartz

*Common*: feldspars

**Matrix**

50% non-calcareous matrix. Reddish/brownish to yellowish/brownish in XP and PPL. High optical activity visible.

**Voids**

20–30% macro vughs and channels. Some full of secondary calcite; randomly oriented.

**Comments**

The fine fraction is not abundant and very fine, the coarse fraction is coarse and well sorted. This indicates that clay has been well cleaned prior to being tempered with fragments of acidic igneous intrusive rock fragments (granite).

| 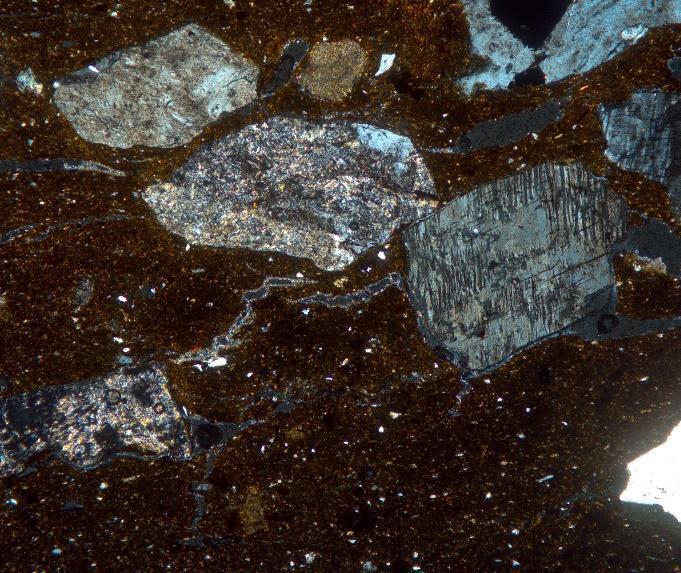 |  |
| --- | --- |
| TM-6, 3 mm field of view |  |

## Petrographic fabric B-TM: Tempered with metamorphic rock fragments and flit.

TM-3, TM-8, TM-10, TM-2, TM-9

**Inclusions**

40%; <3.04 mm; eq.-el.; a.-r.; close to open spaced; weakly bimodal; poorly sorted; randomly oriented.

**Coarse Fraction**

70% < (0.56-3.04 mm)

*Dominant:* foliated metamorphic rock; el. sa.-sr.< 2.00 mm, mode=0.96 mm. Composed of quartz and muscovite and sometimes amphiboles.

*Common:* quartz; eq. a. < 0.6 mm, mode=0.08 mm.

*Common to few:* flint; el. sa.-sr. < 1.76 mm, mode=0.64 mm.

*Few:* calcite; el. sr.-r.< 2.32 mm, mode=0.56 mm.

*Very few:* clastic sedimentary rocks (sandstones); el. a.-sa. < 1.52 mm, mode=0.88 mm.

**Fine Fraction**

30% (0.01-0.24 mm)

*Dominant*: quartz

*Common*: muscovite

**Matrix**

50% non-calcareous matrix. Brownish/yellowish in XP and PPL. High optical activity visible.

**Voids**

10% meso and macro vughs. elongated. Mostly parallel to the edges of the fragments.

**Comments**

The fine fraction is not abundant and well sorted, the coarse fraction is moderately sorted. This indicates that clay has been well cleaned prior to being tempered with sand rich with metamorphic rocks.

| 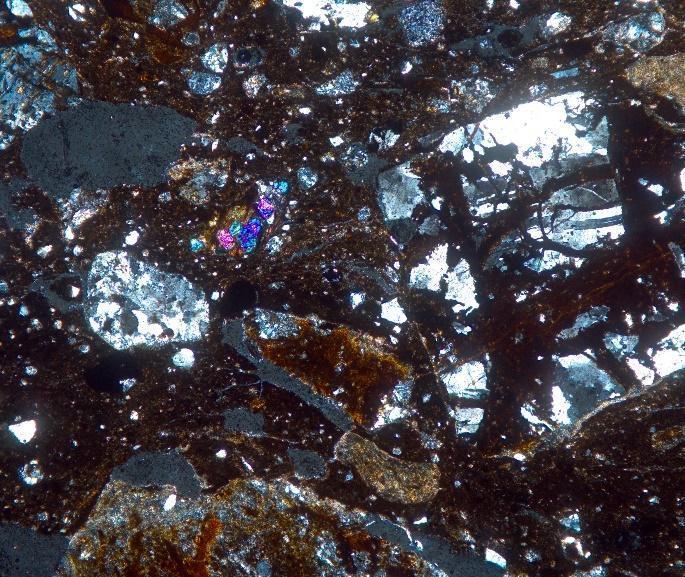 | 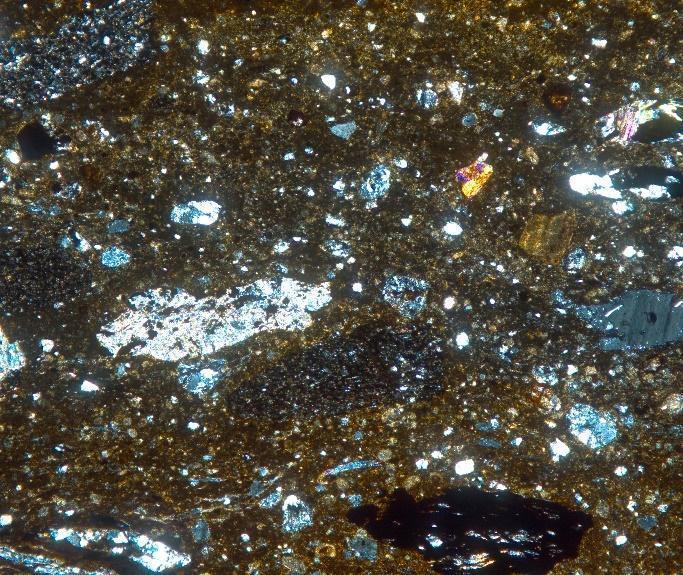 |
| --- | --- |
| TM-10, 3 mm field of view | TM-8, 3 mm field of view |

## Loners:

### Petrographic fabric C-TM: Tempered with flint

TM-7

**Inclusions**

40-50%; <5.04 mm; eq.-el.; a.-r.; close to single spaced; weakly bimodal; poorly sorted; randomly oriented.

**Coarse Fraction**

65%,(0.08-5.04 mm)

*Frequent:* flint; el. sr.-r. < 5.04 mm, mode=2.80 mm.

*Few:* quartz; eq. a.< 0.16 mm, mode=0.08 mm.

*Few:* feldspars; el. a.-sa. < 1.12 mm, mode=0.32 mm.

*Very few:* foliated metamorphic rock; el. sa.-sr. < 4.32 mm, mode=0.88mm. Composed of quartz and muscovite.

*Rare:* calcite; el. sr.< 1.8 mm, mode=0.96 mm

*Very rare to absent:* basic igneous volcanic fine-grained rock fragments with a porphyritic microstructure and phenocrystals of plagioclase and biotite matrix; el. a. < 2.64 mm, mode=2.24 mm.

*Very rare to absent:* chert; el. sa. < 0.30 mm, mode=0.20 mm.

**Fine Fraction**

35% (0.08-0.32 mm)

*Frequent*: quartz and feldspars

*Few*: micrite

**Matrix**

30-40% non-calcareous matrix. Reddish brownish in XP and PPL. Low optical activity visible.

**Voids**

20%; meso and macro vughs; equant and elongate randomly oriented.

**Comments**

The bimodality suggests tempering with sand rich in flint.

| 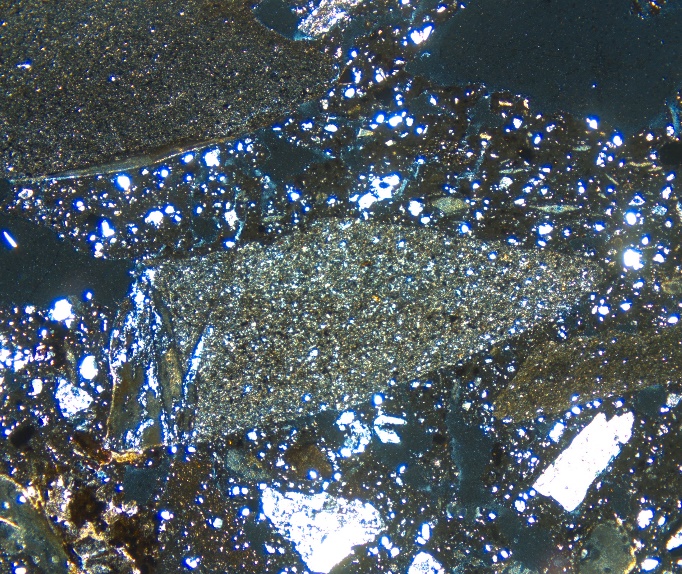 |  |
| --- | --- |
| TM-7, 3 mm field of view |  |

### Petrographic fabric D-TM: Polymodal with calcite

TM-1

**Inclusions**

30%; <1.36 mm; eq.-el.; a.-sr.; single to open spaced; polymodal; poorly sorted; randomly oriented.

40%, (0.08-1.36 mm)

*Frequent:* feldspars; eq.-el. a.-sa. (< 0.88 mm, mode=0.48 mm). Plagioclase, k-feldspars, perthite.

*Few:* quartz; eq. a.-sa. < 0.40, mode=0.08 mm.

*Few:* calcite; eq.-el. a.-sr. < 0.96 mm, mode=0.32 mm.

*Very few:* acidic igneous plutonic coarse-grained rock fragments; el. a.-sa. < 1.36 mm, mode=0.80 mm. Plagioclase, k-feldspars, perthite (granite).

*Rare:* muscovite; el. sa. < 0.16 mm, mode=0.08 mm.

*Rare:* opaque minerals; eq.-el. a.-sa. < 0.56 mm, mode=0.32 mm.

*Very rare:* amphibole; el. a.-sa.< 0.16 mm, mode=0.08 mm.

*Very rare to absent:* foliated metamorphic rock fragment; el. sr. <0.48 mm. Composed of quartz.

**Matrix**

60% non-calcareous matrix. Reddish/brownish in XP and brownish in PPL. High optical activity visible.

**Voids**

10% micro and meso vughs and planar voids; randomly oriented.

**Comments**

The grain size distribution is polymodal, suggesting the inclusions are naturally occurring.

| 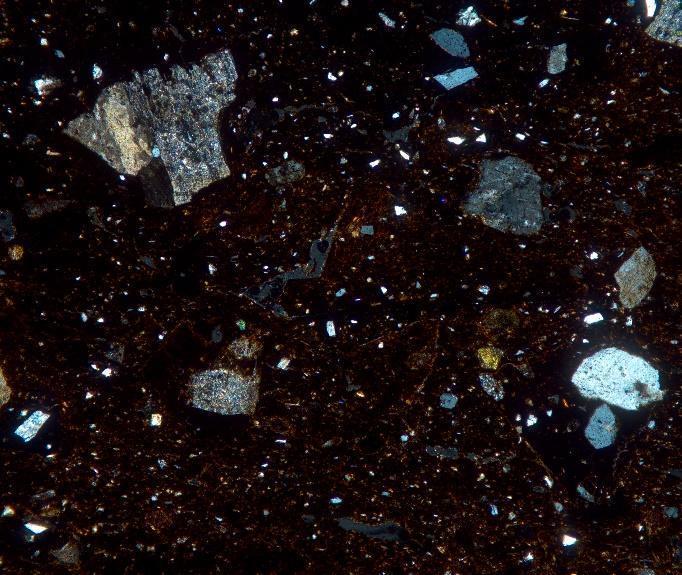 |  |
| --- | --- |
| TM-1, 3 mm field of view |  |

### Petrographic fabric E-TM: Tempered with intermediate igneous volcanic rocks fragments

TM-5

**Inclusions**

30-40%; <2.54 mm; eq.-el.; a.-r.; single to open spaced; weakly bimodal; poorly sorted; randomly oriented.

**Coarse Fraction**

60 %, (0.24-2.56 mm)

*Frequent:* intermediate igneous volcanic fine-grained s rock fragments with a porphyritic microstructure and phenocrystals of plagioclase and amphibole (andesite); el. a.-sa.< 2.56 mm, mode=1.60 mm.

*Few:* feldspars; eq.-el. a. < 0.24 mm, mode=0.08 mm. Plagioclase.

*Few:* quartz; eq. a. < 0.16 mm, mode=0.08 mm.

*Very rare:* calcite; el. sr. < 1.20 mm, mode=0.48 mm.

*Very rare:* opaque minerals; el. r.< 0.48 mm, mode=0.24 mm.

*Very rare:* epidote; eq. r.< 0.1 mm.

**Fine Fraction**

40% (0.01-0.20 mm)

*Dominant*: quartz

*Common*: feldspars Common: calcite

**Matrix**

55-60% non-calcareous matrix. Yellowish/brownish in XP and PPL. High optical activity visible.

**Voids**

5–10% meso vughs and macro channels, with secondary calcite at the edges; randomly oriented.

**Comments**

The bimodality suggests tempering with intermediate igneous volcanic rocks fragments

| 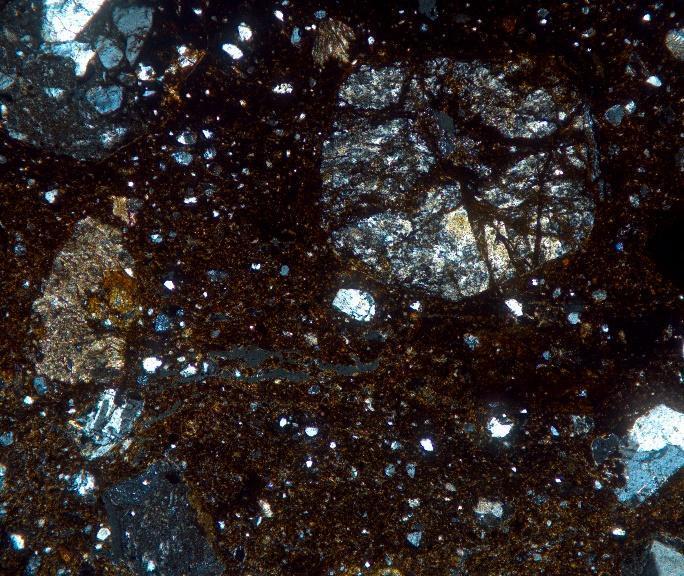 |  |
| --- | --- |
| TM-5, 3 mm field of view |  |

# Kulzhabacy

## Petrographic fabric A-KL: Granite tempered

KL-1, KL-2, KL-3, KL-4

### Subfabric A1-KL: Weakly bimodal

KL-1, KL-2

**Inclusions**

40%; <1.60 mm; eq.-el.; a.-sr.; close to double spaced; weakly bimodal; poorly sorted; randomly oriented.

**Coarse Fraction**

40%, (0.16-1.60 mm)

*Frequent:* feldspars;eq.-el. sa. < 0.64 mm, mode=0.16 mm. Plagioclase, k-feldspar, perthite.

*Frequent:* quartz; eq.-el. sa. < 0.40 mm, mode=0.16 mm.

*Few:* acidic igneous plutonic coarse-grained rock fragments; el. sa. < 1.44 mm, mode=1.04 mm. Composed of plagioclase, k-feldspar, quartz, perthite, amphibole, opaque minerals, biotite (granite).

*Rare:* amphibole; el. a.-sa. < 1.12 mm, mode=0.40 mm (only in KL 2)

*Rare:* calcite; el. sr. < 0.88 mm, mode=0.40 mm

*Very rare:* chert; el. sa.-sr. < 0.46 mm, mode=0.32 mm (only in KL 1)

**Fine Fraction**

60% (0.01-0.16 mm)

*Dominant*: quartz

*Common*: feldspars

**Matrix**

50% non-calcareous matrix. Brownish to blackish in XP and PPL. Low to medium high optical activity visible.

**Voids**

10% meso and macro vughs; randomly oriented.

**Comments**

This weakly bimodal, poorly sorted fabric is marked by the presence of few fragments of igneous plutonic rock fragments, possibly. Inclusion of feldspars and quartz are frequent. Additionally, biotite, amphiboles, calcite and chert can be observed. The fine fraction is composed only of quartz and feldspars. The weak bimodal distribution suggests tempering with fragments of granite.

| 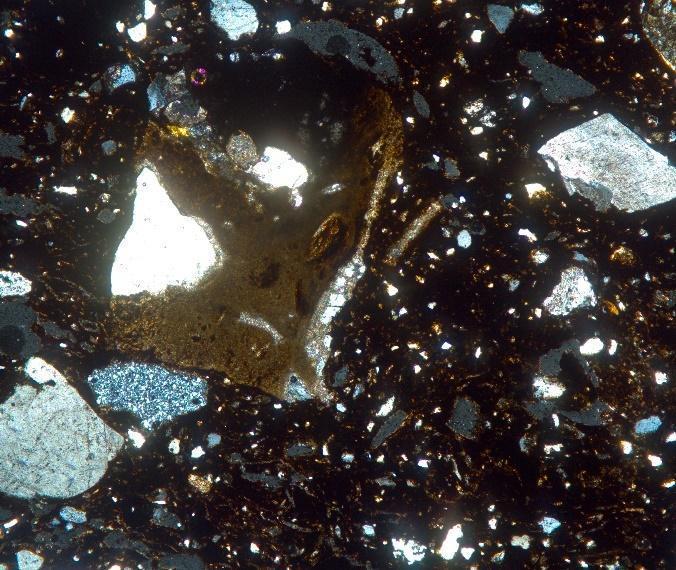 | 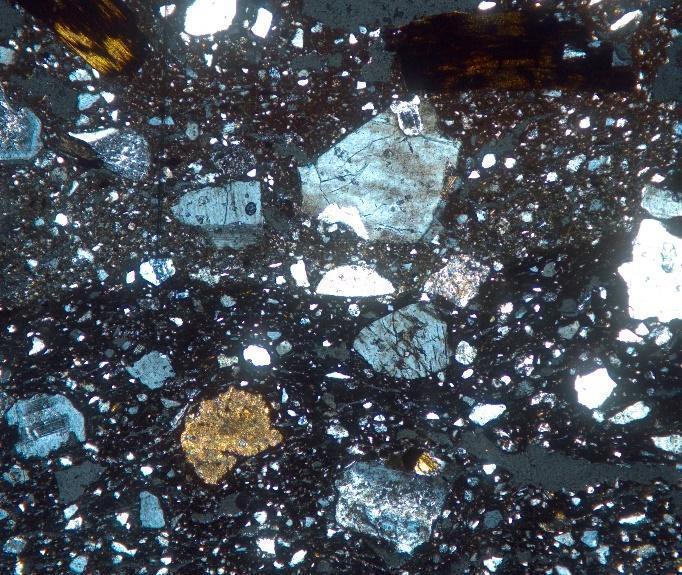 |
| --- | --- |
| KL-1, 3 mm field of view | KL-2, 3 mm field of view |

### Subfabric A2-KL: Strongly bimodal

KL-3, KL-4

**Inclusions**

50%; <3.20 mm; eq.-el.; a.-sr.; close to double spaced; strongly bimodal; moderately sorted; randomly oriented.

**Coarse Fraction**

60-70% (0.20-3.20 mm)

*Frequent:* feldspars; eq.-el. a.-sa. < 1.04 mm, mode=0.2mm. Plagioclase and microcline.

*Common:* acidic igneous plutonic coarse-grained rock fragments; el. sa. < 3.20 mm, mode=1.68 mm. Composed of plagioclase, perthite, k-feldspar, amphibole (granite).

*Few:* quartz; eq. a.-sa. < 0.5mm, mode: 0.2 mm,

Very few: basic igneous volcanic fine-grained rock; el. sa. < 1.5 mm, mode=1.4 mm. Composed of feldspars in plagioclase laths (basalt). Only in KL 3.

*Rare:* calcite (weathered); el. sr.< 0.48 mm, mode=0.24 mm.

*Rare:* amphibole; el. a.-sa. < 1.60 mm, mode=0.48 mm.

*Very rare to absent:* limestone; eq. sa. <1.52 mm (only in KL 3)

**Fine Fraction**

30-40% (0.01-0.16 mm)

*Dominant*: quartz

*Common*: feldspars

**Matrix**

40% non-calcareous matrix. Brownish to yellowish in PPL and brownish to blackish in XP. Medium high optical activity visible.

**Voids**

10% meso and macro vughs. In some cases, secondary calcite at the rims visible; randomly oriented.

**Comments**

This fabric is marked by the common occurrence of coarse acidic igneous plutonic rock fragments, possibly granite. In addition, feldspars (plagioclase and few microcline), quartz, calcite, amphibole, and fragments of a basic extrusive rock, probably basalt, can be observed. One fragment of limestone is visible in sample KL-3. The strong bimodality and relatively good sorting of the inclusions fine fraction suggest that this the clay was cleaned prior to tempering with granite fragments that are coarse in comparison to subfabric A1-KL.

| 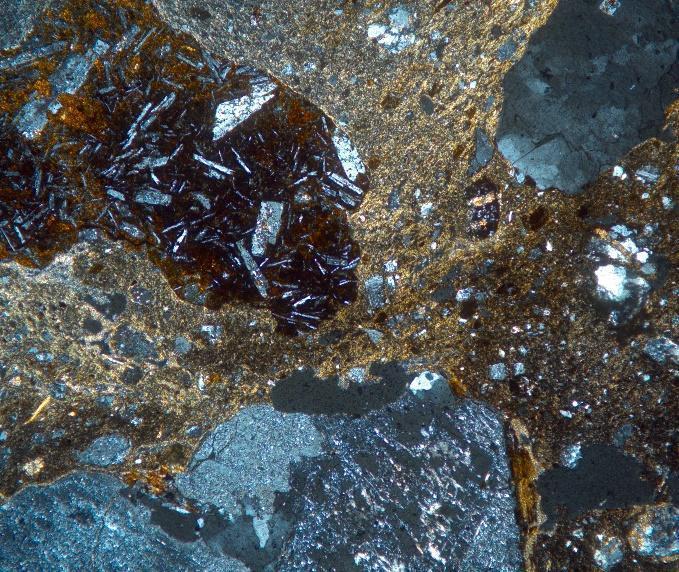 | 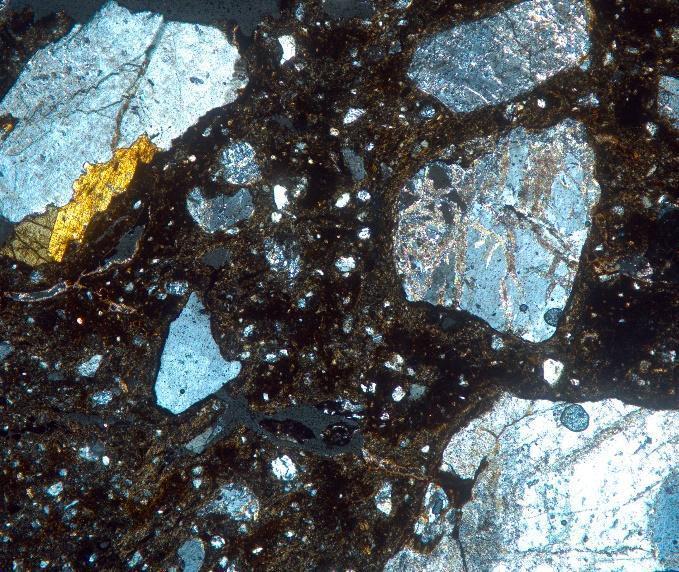 |
| --- | --- |
| KL-3, 3 mm field of view | KL-4, 3 mm field of view |
